# Efficient Green Light Acclimation of the Green Algae *Picochlorum sp.* Triggering Geranylgeranylated Chlorophylls

**DOI:** 10.3389/fbioe.2022.885977

**Published:** 2022-04-28

**Authors:** Michael Paper, Matthias Glemser, Martina Haack, Jan Lorenzen, Norbert Mehlmer, Tobias Fuchs, Gerhard Schenk, Daniel Garbe, Dirk Weuster-Botz, Wolfgang Eisenreich, Michael Lakatos, Thomas B. Brück

**Affiliations:** ^1^ Werner Siemens-Chair of Synthetic Biotechnology, Department of Chemistry, Technical University of Munich, Garching, Germany; ^2^ TUM AlgaeTec Center, Ludwig Bölkow Campus, Department of Aerospace and Geodesy, Taufkirchen, Germany; ^3^ School of Chemistry and Molecular Biosciences, The University of Queensland, Brisbane, QLD, Australia; ^4^ Sustainable Minerals Institute, The University of Queensland, Brisbane, QLD, Australia; ^5^ Institute of Biochemical Engineering, Faculty of Mechanical Engineering, Technical University of Munich, Garching, Germany; ^6^ Chair of Biochemistry, Department of Chemistry, Technical University of Munich, Garching, Germany; ^7^ Integrative Biotechnology, University of Applied Sciences Kaiserslautern, Pirmasens, Germany

**Keywords:** green light, photosynthesis, chlorophyll derivatives, light adaption mechanism, geranylgeranylated, eukaryotic microalgae

## Abstract

In analogy to higher plants, eukaryotic microalgae are thought to be incapable of utilizing green light for growth, due to the “green gap” in the absorbance profiles of their photosynthetic pigments. This study demonstrates, that the marine chlorophyte *Picochlorum sp*. is able to grow efficiently under green light emitting diode (LED) illumination. *Picochlorum* sp. growth and pigment profiles under blue, red, green and white LED illumination (light intensity: 50–200 μmol m^−2^ s^−1^) in bottom-lightened shake flask cultures were evaluated. Green light-treated cultures showed a prolonged initial growth lag phase of one to 2 days, which was subsequently compensated to obtain comparable biomass yields to red and white light controls (approx. 0.8 g_DW_ L^−1^). Interestingly, growth and final biomass yields of the green light-treated sample were higher than under blue light with equivalent illumination energies. Further, pigment analysis indicated, that during green light illumination, *Picochlorum sp*. formed unknown pigments (X1-X4). Pigment concentrations increased with illumination intensity and were most abundant during the exponential growth phase. Mass spectrometry and nuclear magnetic resonance data indicated, that pigments X1-X2 and X3-X4 are derivatives of chlorophyll *b* and *a*, which harbor C=C bonds in the phytol side chain similar to geranylgeranylated chlorophylls. Thus, for the first time, the natural accumulation of large pools (approx. 12 mg g_DW_^−1^) of chlorophyll intermediates with incomplete hydrogenation of their phytyl chains is demonstrated for algae under monochromatic green light (Peak *λ* 510 nm, full width at half maximum 91 nm). The ability to utilize green light offers competitive advantages for enhancing biomass production, particularly under conditions of dense cultures, long light pathways and high light intensity. Green light acclimation for an eukaryotic microalgae in conjunction with the formation of new aberrant geranylgeranylated chlorophylls and high efficiency of growth rates are novel for eukaryotic microalgae. Illumination with green light could enhance productivity in industrial processes and trigger the formation of new metabolites–thus, underlying mechanisms require further investigation.

## Introduction

Microalgae are a diverse group of photoautotrophic pro- and eukaryotic microorganisms, having the capacity of light-dependent CO_2_ fixation to generate value-adding biomass. Over the last decades, advanced cultivation methods that allow for rapid biomass formation without land use change have led to ever more industrial applications for these third-generation cell factories. In this context, microalgae can generate e.g. high concentrations of intracellular proteins (Bleakley and Hayes 2017), or polyunsaturated long-chain fatty acids (Nakamura and Li-Beisson 2016) used in nutraceutical and cosmetic applications, respectively. Particularly, algae-based pigments, such as β-carotenes and astaxanthin, have high-value applications in the pharmaceutical, cosmetic, and food industries (Del Campo, Moreno et al., 2000; Lorenz and Cysewski 2000; Mata, Martins et al., 2010; Liu, Chen et al., 2016; An, Gao et al., 2017). Therefore, the biotechnological production of pigments and identification of new derivatives thereof are of high interest to offset the costs of biomass production and product extraction (Olivieri, Salatino et al., 2014). Recent studies have shown that the addition of specific chemicals to the growth medium can enhance total biomass production and oil content in certain algae strains (Franz, Danielewicz et al., 2013; Paliwal and Jutur 2021). Alternatively, the optimization of illumination during algae cultivation can lead to increased productivity. Microalgal pigments comprise the three major classes of chlorophylls, carotenoids, and phycobilins. The latter are being restricted to prokaryotic algae (cyanobacteria) as well as specific eukaryotic groups such as glaucophytes, red algae, and some cryptomonads (Toole and Allnutt 2003). The most important light-harvesting pigment class detected in all microalgae are the chlorophylls. Comprehensively, chlorophylls constitute a central protoporphyrin IX-type scaffold. Selective insertion of a Mg^2+^ ion into the tetrapyrrole ring system by magnesium chelatase generates the photon-harvesting chlorophyll chromophore (Fiedor, Kania et al., 2008). The subsequent addition of a phytol sidechain to the chromophore constitutes the terminal step in chlorophyll biosynthesis (Von Wettstein, Gough et al., 1995; Brzezowski, Richter et al., 2015). The phytol moiety comprises up to one third of the molecule’s mass (however missing in Chl *c*) and thus enhances its lipophilic character, which facilitates chlorophyll anchoring on the thylakoid membrane and on lipophilic protein cavities (Chaves, Amorim et al., 2015; Cao, Li et al., 2021). The chemical nature of the phytol side chain modulates the light absorption properties (Fiedor, Stasiek et al., 2003). The two major chlorophyll variants in chlorophytes Chl *a* and *b* only differ in the functional group at the C7 position of the protoporphyrin IX scaffold. While Chl *a* features a methyl group in that position, Chl *b* is oxidized to an aldehyde group. The structural identification of chlorophylls was first described by Hans Fischer (Fischer 1937; Woodward 1960), while the corresponding nomenclature was later updated in accordance with IUPAC rules (Merritt and Loening 1980) (see Supplementary Figure S1). Specifically, Chl *a* is part of the photosynthetic pigment-protein reaction center in all oxygenic photoautotrophs. Further, the accessory antennae pigments Chl *b*, Chl *c* (mainly in diatoms, dinophyta and brown algae) and Chl *d* (red algae) diversify the range of light absorption in the light-harvesting complex (Masojıdek, Koblızek et al., 2004). Moreover, the Chl *f* found in some cyanobacteria is a type of chlorophyll that absorbs further in the infrared radiation (Nürnberg, Morton et al., 2018). However, all chlorophylls have absorption bands mainly in the blue (430–475 nm) and the red light region (630–696 nm), resulting in their characteristic green color by transmission. The extensive taxonomic and ecologic diversity of pro- and eukaryotic microalgae demonstrates their adaptation potential to different environmental and particular light challenges. Due to the main light absorption in the blue and red region, most photosynthetic organisms such as plants and microalgae use the blue and red spectrum of the photosynthetic active radiation (PAR). While green light is less effectively utilized for growth because of the improperly named “green gap” between approximately 500 and 600 nm of their light-harvesting machinery. The efficient use of green light for growth has been reported for prokaryotic cyanobacteria, such as *Arthrospira platensis* (Walter, Carvalho et al., 2011; Markou 2014; Smith, McAusland et al., 2017). Specifically, cyanobacteria harbor the phycobilisome-associated photosynthetic pigments, phycocyanin, and phycoerythrin, which extend the light-harvesting complex of these organisms, facilitating the utilization of green light and the rapid adaptation to low light environments (Kehoe and Grossman 1994; Chukhutsina, Bersanini et al., 2015; Singh, Sonani et al., 2015). In analogy to higher plants, green algae lack these phycobilisome-associated pigments. Hence, it is currently thought that most eukaryotic algae do not have sufficient mechanisms to utilize green light for efficient growth (Yan, Zhang et al., 2013; Vadiveloo, Moheimani et al., 2015). Nevertheless, few species-specific exceptions are reported where green light was effectively used by the green algae *Ettlia sp*. out-competing *Chlorella vulgaris* by better biomass growth (Lee, Seo et al., 2019). Also, the Haptophyta *Isochrysis galbana* showed an increase in photosynthetic efficiency at green light, which was associated with a light absorption close to that for cells cultivated at white light (Li and Liu 2020). The penetration of the light is physically influenced by cell density, the length of the light path, and light intensity leading to more efficient absorption and conversion into biomass production under green light at high cell density, long light path and high light intensities (Ooms, Dinh et al., 2016). The fact that cultures under green light and at high cell density have higher biomass production was demonstrated in *C. vulgaris* (Mohsenpour and Willoughby 2013), *Scenedesmus bijuga* (Mattos, Singh et al., 2015) and *Ettlia* sp. (Sarrafzadeh, La et al., 2015) as well as in the cyanobacteria *Synechococcus elongatus* (Ooms, Graham et al., 2017). Apart from facilitating the utilization of green light by phycobilisomes in cyanobacteria glaucophytes, red algae, and some cryptomonads, no other physiological mechanisms increasing the photosynthesis for microalgae are currently known. Recently, novel pigment accumulations of chlorophyll derivates at green light treatments were observed in higher plants for the first time (Materová, Sobotka et al., 2017). Barley (*Hordeum vulgare*), basil (*Ocimum basilicum*), sunflower (*Helianthus annuus*), Norway spruce (*Picea abies*) and, with lower amount, amaranth (*Amaranthus* sp.) accumulated a large pool of geranylgeranyl-Chl *a* cultivated under green light conditions. However, the efficiency of photosynthesis was low. The accumulation of geranylgeranylated chlorophylls under green light was also observed for *Arabidopsis thaliana* in a study from 2021 (Karlický, Materová et al., 2021). In this study, the comparative growth response of the eukaryotic green algae *Picochlorum* sp. under discrete white, red, blue, and green light exposure has been investigated using advanced LED illumination techniques in combination with a detailed pigment analysis. *Picochlorum* sp. shows a pronounced tolerance towards high salinities and fluctuating environmental conditions (Foflonker, Price et al., 2015)*.* Recent studies showed it is a promising candidate for several industrial applications like wastewater remediation or the production of lipids and food additives (Yang, Xiang et al., 2014; Woortman, Fuchs et al., 2020; Goswami, Agrawal et al., 2021). *Picochlorum* sp. growth and biomass yield under green light was, after a short adaptation period, comparable to or higher than growth rates measured with illumination at other wavelengths. We conducted a comprehensive pigment analysis for illumination under different light conditions in quantity and quality. Therefore, three different experimental setups were performed with different colored LEDs, with a specific spectrum of green light, and with scale up performance to study the effects of cell concentration and light penetration. Next to the compositional alteration in the carotene pigment class, we intensified the analyses of chlorophylls because the formation of new chlorophyll variants was detected, initially *via* HPLC analysis. The subsequent characterization of these chlorophyll variants was conducted by HPLC-MS and NMR and demonstrated, for the first time in microalgae, the accumulation of geranylgeranyl-Chl *a* + *b* under green light conditions.

## Materials and Methods

### Strain and Media

In this study, the microalgae *Picochlorum* sp. (Trebouxiophyceae, Chlorophyta), an original isolate from Salt Lake Pond, San Salvador, Bahamas (24°01′40.7″N, 74°26′58.7″W), and propagated in our in-house strain collection, was used. The strain was identified with 99% identity as *Picochlorum sp*. SENEW3 (NCBI Accession-No.: KF591594). For cultivation, a modified Artificial Seawater (ASW) medium (Boussiba, Vonshak et al., 1987) with KNO_3_ (5 g L^−1^) and a modified trace element solution (1 ml L^−1^) was used. The trace element solution was comprised of MnCl_2_ * 4 H_2_O (0.628 g L^−1^), H_3_BO_3_ (0.6 g L^−1^) (NH_4_)_6_Mo_7_O_24_ * 4 H_2_O (0.37 g L^−1^), ZnCl_2_ (0.04 g L^−1^) and CuCl_2_ * 2 H_2_O (0.04 g L^−1^), and was filter-sterilized and added after autoclaving. The pH was set to 8.2. For shake flask experiments the medium was inoculated with the seed culture prior to being aliquoted into individual shake flasks.

### LED Setup and Cultivation Systems

**FIGURE 1:**
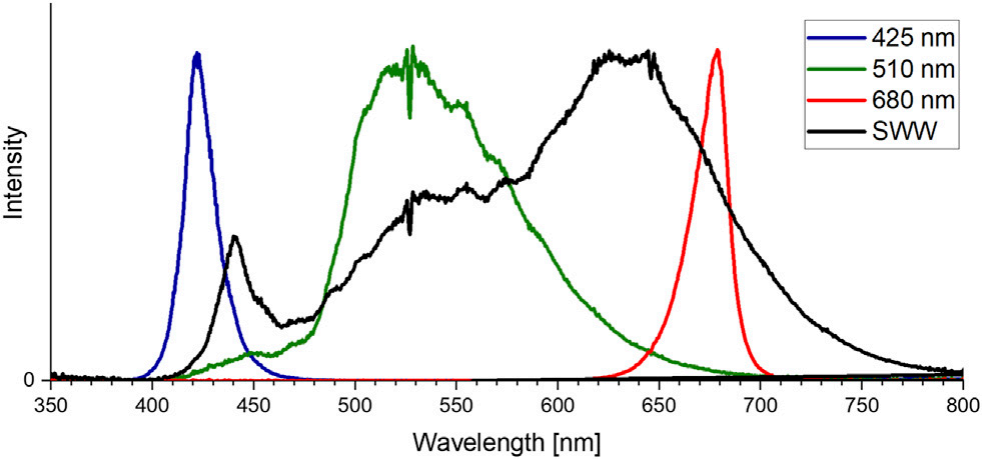
Light-emitting diode (LED) intensities (425 nm, 510 nm, 680 nm and SWW) used for color growth experiments. Ocean optic spectrometer data was normalized to fit the 425 nm peak.

**FIGURE 2:**
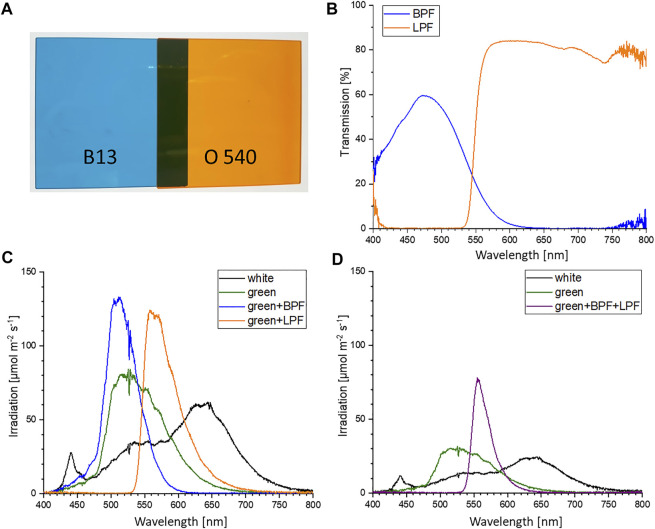
**(A)** Colored glass filters: band-pass filter B 13 (BPF) and long-pass filter O 540 (LPF) (LaserComponents 2022) **(B)** Transmission values of the BPF and LPF. Values obtained *via* warm-white light emitting diode (LED) illumination and ocean optic spectrometer measurements. **(C)** Irradiation intensities of warm-white LED (SWW), green LED (510 nm), green LED with BPF and green LED with LPF at setpoint 200 μmol m^−2^ s^−1^. **(D)** Irradiation intensities of warm-white LED (SWW), green LED (510 nm) with BPF and LPF were set to 75 μmol m^−2^ s^−1^.

Three different experiments were performed. A) Color growth experiments were conducted with monochromatic light colors with blue, green, red, and white LEDs to study the performance of algal biomass growth and pigment production. B) To differentiate the effect of concomitant irradiance the second experiment focused on the photo-induced performance of green light in the narrow sense/*sensu stricto* by cutting the transition wavelength transmitted by the green LED with portions of blue and orange color. C) The verification and scale up for pigment isolation were conducted to evaluate whether the observation of unknown pigment formation during green light illumination might be a shake flask cultivation artifact. Thus, the experiments were repeated in commercial, controlled stirred tank photobioreactors at conditions of dense culture, long light pathways and comparable light intensities. An overview of the conducted cultivation experiments is given in Table 1. The experiments in shake flasks were conducted in a customized shaker unit consisting of a water-cooled platform with 18 shaker flask mounts with individual bottom-lit LED illumination systems (see Supplementary Figure S2A) developed together with the company FutureLed (Futurled 2022), which was installed in a 44 unit shaker (New Brunswick InnovaTM, Eppendorf, Hamburg, Germany). The LED-mounts (see Supplementary Figure S2B) individually allow a bottom-up illumination of 500 mL shake flasks with several LEDs, allowing a shade-free algae culture illumination with each having individual irradiation settings. Cross-illumination from adjacent shake flasks was prevented by shading each flask individually with a black plastic wrapping. Calibration and setting to desired irradiance levels were performed via a spectrometer (Ocean optic STS-VIS, Ocean Insight, Ostfildern, Germany), utilizing the Ocean View Software (version 1.5.2) with 20 scans in the wavelength range of 400 - 800 nm. The 44 shaker units, equipped with the LED-platform, were additionally improved with a custom built aeration system, powered by a mass-flow control unit (DASGIP® MX4/4, Eppendorf). The aeration system was connected to the unbaffled Erlenmeyer flasks (shown in Supplementary Figure S2). Each flask was sealed airtight with a custom-made rubber stopper, including air-inflow and exhaust filters. To ensure equal pressure and sterility, the air-distribution for the connected flasks used an air-inlet filter with a pore size of 0.45 µm, whereas the exhaust filter had 0.2 µm pores. Aeration was set for all experiments at 1.8 L h -1 per flask, with 1 % (v/v) CO2-enriched air. In experiments with 200 µmol photons m-2 s-1, the CO2 enrichment was set to 2 % (v/v) to avoid an untimely pH shift during the shake flask experiments. For identical inoculation of multiple shake flasks, fresh ASW medium (see previous section) was inoculated in a sterile 5 L glass bottle with a seed culture in exponential growth phase up to a starting OD750nm of 0.05. The well mixed culture volume was then transferred to the individual shake flasks. Each 500 mL flask was filled with 200 mL. With an eccentricity of 2.5 cm and 150 rpm shaking speed this resulted in a culture depth of approximately 1.5 cm. All growth experiments were conducted at 25 °C over 17 days. For the color growth experiments (A), the LEDs used for algae culture illumination were installed in the previously described bottom lit shake flask array. The light spectrum of each LED used for this setup is displayed in Figure 1. Each spectrum is normalized to match the total intensity of the 425 nm peak of the blue LED. The total photosynthetic photon flux density (PPFD) setup for the experiments for 50, 100, 150, and 200 μmol m^−2^ s^−1^ was integrated over the full spectrum of photosynthetically active radiation (PAR) between 400 and 750 nm. The bandwidth of the standard warm white (SWW) LED, with a broad bandwidth of 163 nm full width at half maximum (FWHM), is shown in Table 2. This LED had, overall, a smaller intensity compared to the sharp peaks for the blue and red LED. For the second cultivation setup (B) for specific wavelength restrictions of LED light spectra, two colored cut-off glass filter plates (LaserComponents 2022) were attached to the LED illumination base. Specifically, a blue-colored band-pass filter B 13 (band-pass filters for blue-green spectrum; BPF) and an orange-colored long-pass filter O 540 (long-pass filter for yellow-orange spectrum; LPF) obtained from Laser Components GmbH (Olching, Germany) were applied (shown in Figure 2A). The transmission efficiency of the glass filters is shown in Figure 2B. Filters were chosen to limit transmission above 610 nm (BPF) and below 530 nm (LPF) to specifically examine the wavelength dependence of green light illumination only. Growth with green light LED, green LED + BPF and green LED + LPF illumination was compared to white LED illumination (Figure 2C). The two glass filters were stacked (Figure 2D) to restrict the illumination wavelength range to 530–610 nm. With this filter setup, the warm-white LED (SWW), green 510 nm and green-yellow 565 nm LED had to be turned on to maximum power to obtain maximal irradiation levels. Irradiation levels were measured *via* a 5-point calibration and the resulting irradiation levels averaged at 75 μmol m^−2^ s^−1^. This value was used to set the green (510 nm) and warm-white (SWW) LED. Therefore, the application of both filter systems on the light spectra reduced the maximal light irradiance to 75 μmol m^−2^ s^−1^ at full LED power, which induced low light conditions. Moreover, scale up experiments (C) were carried out in 3.7 L glass bioreactors (Labfors 5 Lux bioreactors, Infors GmbH, Einsbach, Germany) and controlled vi*a* the Infors IRIS software tool (Franco 2014; Infors 2017). Cultures were aerated with 0.5 vvm (with stepwise increase of CO_2_ addition in 30 s increments to adjust pH to setpoint 8.2). The 3.7 L glass reactors were stirred at 150 rpm and filled with to maximum working volume of 2.3 L with an average culture depth of approximately 6.5 cm. The temperature was set to 25 C. An external illumination system was developed and built in cooperation with the company FutureLed, Berlin, Germany (Futurled 2022), equipped with the same LEDs as the LED-shaker platform shown in Table 2 and Figure 1 and calibrated *via* an ocean optic spectrometer (Ocean Insight, Ostfildern, Germany). The light intensity was set to 150 μmol m^−2^ s^−1^PPFD.

**TABLE 1:** Overview of the experimental setup for the cultivation under different illumination conditions.

Setup	Color growth experiments (A)	Specific wavelength restriction (B)	Scale up (C)
Platform	500 ml shake flask	500 ml shake flask	3.7 L photobioreactor
Colors	white, blue, red, green	white, green, green + BPF + LPF	white, green
Irradiation [µmol m^−2^ s^−1^]	50, 100, 150, 200	75	150
CO_2_	1-2%	1%	adjusted for pH-regulation
Biological replicates	3–4	3	2

**TABLE 2:** Light-emitting diode (LED) properties of the installed LED in each illumination base. SWW is a standard warm white LED. Peak *λ*, full width at half maximum (FWHM) and max irradiation determined *via* spectrometer.

Color	Peak *λ* [nm]	FWHM [nm]	Max irradiation [µmol m^−2 ^s^−1^]
white	SWW	163	1764
blue	425	17	473
green	510	91	868
red	680	20	776

### Growth Analysis

Measurements of optical density of bioreactor and shake flask pre-cultures were performed by a photometer (Hewlett Packard 8,453, HP/Agilent, Santa Clara, United States), with sample volumes of 1 ml and standard semi-micro cuvettes made of polystyrene (PS). Optical densities were measured at 750 nm with 3% (w/v) NaCl solution for the dilution of samples. Optical density measurements of shake flasks cultures were performed on a Perkin Elmer EnSpire2 microtiter plate (MTP) reader (Perkin Elmer, Waltham, United States) using 96-well plates (Sarstedt TC-Plate 96 Well Standard F) with a volume of 200 μL at OD = 750 nm. Each measurement was carried out in biological triplicates and within the linear range of the corresponding device (max. absorption of OD_750nm_ = 0.6). A 3% (w/v) NaCl solution was applied as control blank. Biomass sampling for cell dry weight analysis (minimum cell dry weight for sampling: 10 mg) commenced twice in the late exponential phase, once when sufficient biomass was generated (not to deplete the entire cultivation volume), and finally at the end of the cultivation. A strain- and color-specific correlation factor for cell dry weight (CDW) and optical density (OD) was established (not shown). The CDW was calculated *via* washed, lyophilized (-80°C, min 48 h) and dried (65 C for 24 h) cells (*m*_2_) (minimum 10 mg sampled in a 50 ml centrifuge tube with screw cap), compared to empty vessels (*m*_1_) as CDW = (*m*_2_-*m*_1_)*V_sample_^−1^, each performed in biological triplicates. CDW data points were processed using R version 4.1.0 (2021-05-18) and Rstudio version 1.4.1717. Specific growth rates were determined with R package grofit 1.1.1-1. The logistic growth model was applied for all growth experiments (Kahm, Hasenbrink et al., 2010). For all gravimetric and spectrophotometric data sets mean values and standard deviation were calculated from respective biological triplicates to generate the graphs with Origin (OriginPro 2017G, OriginLab Corporation, Northampton, United States) for data representation.

### Pigment Extraction

Pigment extraction was adapted from (Roy, Llewellyn et al., 2011), using 10-15 mg dry biomass per sample. All steps were performed on ice and under dark conditions. Briefly, washed biomass was stored as pellets at -20 C until extraction. The pellets were slowly thawed on ice for 30 min prior to extraction. Each pellet was suspended in 2 ml 90% (v/v) HPLC-grade acetone in ddH_2_O and promptly transferred to a precooled 10 ml glass tube with solvent-proof screw-top lid. With additional 2 ml acetone the tube was washed to retrieve all pigments from the pellet. Two 5 mm glass beads were added to break the cells during vortex-mixing (maximum speed) for 10 s (Scientific Industries SI™ Vortex-Genie™ 2, Bohemia, United States). After 20 min of ultrasonic extraction in an ice-chilled water bath (Ultrasonic cleaner, VWR, Dietikon, Switzerland), the samples were vortex-mixed again for 10 s. Pigment extracts were stored over night at -20 C. After slow thawing for 30 min on ice and another 10 s of shaking on the vortex mixer, the pigment extract was transferred to a 2 ml syringe and filtered through a 0.2 µm PTFE filter, directly into GC vials. The vials were directly transferred into the shaded/cooled HPLC autosampler (4°C; Agilent 1100 HPLC, Agilent Technologies, Santa Clara, United States), or wrapped (light-proof) and stored temporarily at -20 C.

### HPLC Based Pigment Analysis

The HPLC analysis protocol was adjusted from a method described by Van Heukelem (Van Heukelem and Thomas 2001). A YMC-Pack Pro C8 Column (250 mm * 4.6 mm I.D.; particle size 5 μm, YMC, Kyoto, Japan) was used in a HP Agilent 1100 HPLC-System (Agilent Technologies, Santa Clara, United States), equipped with a diode array detector (Agilent 1,100) operating at 450 nm. The two-buffer system, consisting of buffer A: 30:70% (v/v) water/methanol and buffer B: 100% methanol, were set to 40% buffer B, with a gradient ramping to 95% within 30 min, held for 5 min and dropped to 40% over 10 min. A flow of 1 ml min^−1^ and 10 µl injection volume were applied. The oven temperature was set to 60 C and the shaded autosampler was kept at 4 C. Chromeleon 6.80 SR12 software (Thermo Fisher Scientific, Germany) was applied to control the HPLC system and for post-experimental analysis. Pigment control standards were obtained from CaroteNature (Lupsingen, Germany) and Sigma-Aldrich Chemie (Weinheim, Germany) in HPLC grade quality. Preparative HPLC was performed for pigment extracts of each biological triplicate, which were vacuum-dried in a GeneVac Atlas Evaporator HT4 (GeneVac, Ipswich, UK) and resolved in small volumes of 100% (v/v) acetonitrile (e.g., 1.5 ml extract dissolved in 300 µl). The subsequent HPLC-based pigment separation was conducted as described above for the analytical procedures, while components of interest were each collected manually for every biological replicate (n = 3). For preparative procedures 100 µl of the resolved extracts were injected. High resolution LC-MS/MS analysis was performed on a LTQ-FT mass spectrometer (Thermo Fisher Scientific, Schwerte, Germany) equipped with an UltiMate3000 HPLC-System. The solvent system was dd H2O (A): 90% (v/v) acetonitrile (B), both spiked with 0.1% (v/v) formic acid. A flow rate of 1.1 ml min^−1^ of 80% B was applied. The sample volume was 1 µl. The autosampler was kept at 15°C, while the oven temperature was set to 22 C. Samples were analyzed by direct injection. The full-scan mass spectra for a range of m/z 210–1,500 were acquired in positive mode, with a resolution of 100,000 (m/z 773.49). MS/MS analysis was obtained with collision induced dissociation (CID), using helium with a collision energy (CE) of 35%. The Xcalibur software (Thermo Fisher Scientific, Schwerte, Germany) was used for the identification of unknown pigments. For the peak identification process a range of potential atom types (such as carbon, oxygen, nitrogen, hydrogen and magnesium) and the quantity of these atoms was implemented. For targeted analysis of Chl *a* mass formula of C_55_H_72_MgN_4_O_5_ was applied as the input parameter. The high resolution LC-MS/MS allowed working with a critical mass tolerance of 1 ppm. Pigments were identified by specific masses.

### Statistical Analysis of Pigment Concentration

A statistical analysis of differences in pigment concentration for biomass obtained from white and green light cultivation was performed *via* a two-tailed *t*-test. Datasets of three repilicates were used for the calculation of mean values, standard deviations, and *p*-values.

### NMR Based Chlorophyll Analysis

Nuclear magnetic resonance (NMR) spectroscopy was performed on a Bruker AV500 Avance-I with TopSpin 2.1 software (Bruker BioSpin MRI GmbH, Ettlingen, Germany) at the central analytics lab of the Department of Chemistry at TUM. As solvent acetone-d_6_ (99.9 atom % D) was chosen to match conditions employed by previously published comparative studies (Kobayashi, Akutsu et al., 2013). The same samples used for LC-MS/MS analytics were analyzed. Samples were placed on ice and the acetonitrile solvent was evaporated by nitrogen ventilation. Dried pigment controls (Chl *a* and *b*) and each sample was resuspended in 120 µL acetone-d6 and transferred to a micro-NMR-tube. The micro-NMR-tubes were shaded until being placed inside the AV500. For all samples the 1H and COSY spectra were recorded. Additionally, Chl *a* and *b* samples were subjected to HSQC and HMBC analyses. Multipoint-baseline correction was obtained manually to alter upfield values and identify peaks. The chemical shifts in the ^1^H spectra were correlated to literature data (Kobayashi, Akutsu et al., 2013), starting from the known structural data of Chl *a* and *b*.

## Results and Discussion

### Shake Flask Experiments at Different LED Colors

**FIGURE 3:**
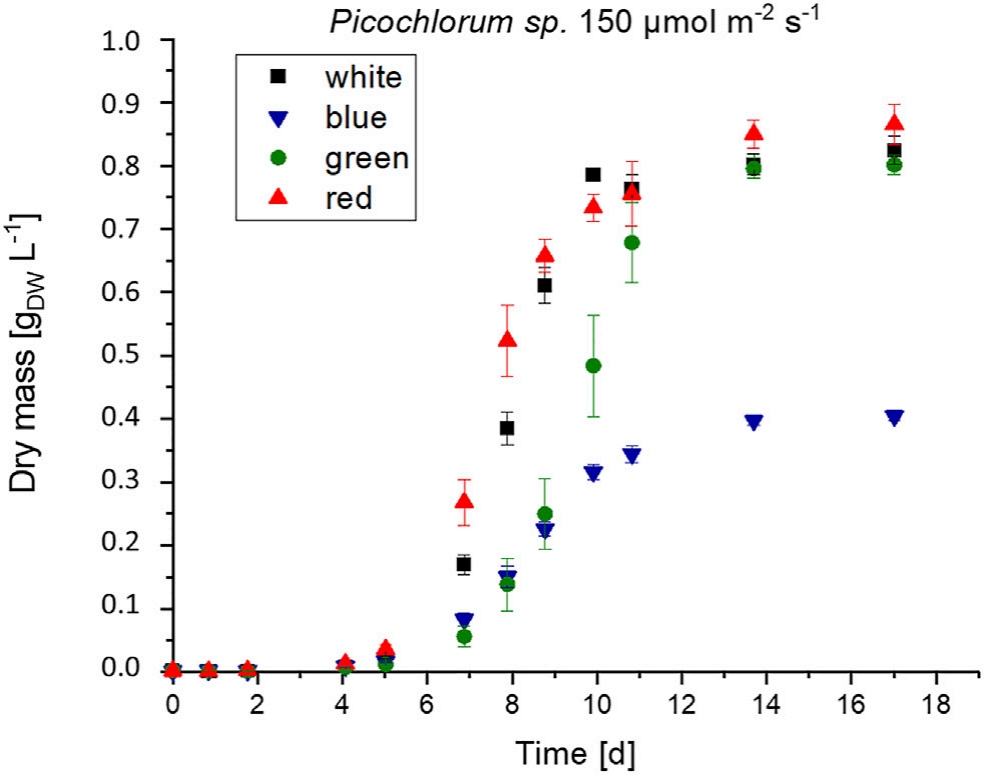
Biomass formation of *Picochlorum* sp. at 150 μmol m^−2^ s^−1^ irradiance with white, blue, green and red light illumination (n = 3).

*Picochlorum* sp. cultures were grown in biological triplicates under white, green, blue, and red light illumination using LEDs with irradiation levels set each to 200, 150, 100, and 50 μmol m^−2^ s^−1^. Algae cultures that were illuminated with green light exhibited a longer initial lag phase but reached similar growth rates and final biomass concentration (see Figure 3).

Under the same illumination conditions, cultivation with blue light resulted in approximately half the maximal growth and biomass formation when compared to white and red illumination conditions. This observation indicates that *Picochlorum* sp. could not use blue light efficiently for photosynthesis. This contrasts the situation with other marine eustigmatophytes, such as *Nannochloropsis* sp., which is reported to preferentially use blue light for growth, biomass, and lipid formation (Das, Lei et al., 2011). Overall, higher growth rates were reached with increased light intensity (see Table 3). Thus, growth in this experimental setup is likely not photo-inhibited but light limited instead.

**TABLE 3:** Maximum growth rates µ_max_ d^−1^ of *Picochlorum* *sp*. at color illumination of 200–50 μmol m^−2^ s^−1^.

Color	200 (n = 4)	150 (n = 3)	100 (n = 3)	50 (n = 3)
white	0.550 ± 0.038	0.268 ± 0.014	0.186 ± 0.016	0.089 ± 0.009
blue	0.240 ± 0.021	0.085 ± 0.002	0.093 ± 0.011	0.040 ± 0.004
green	0.606 ± 0.043	0.218 ± 0.008	0.167 ± 0.009	0.094 ± 0.011
red	0.529 ± 0.065	0.215 ± 0.019	0.196 ± 0.016	0.075 ± 0.011

Initial spectrophotometric-based pigment analysis indicated, that the total pigment content decreased gradually over the course of three sample points on days 9, 11, and 17 (end of cultivation). This pigment decrease was consistent with a reduction in new biomass formation towards the end of the cultivation. Lutein, Chl *a* and *b* were the major pigments, with further spectral signals for *β*-carotene and canthaxanthin, that were also in agreement with literature data (Singh 1975; Fisher, Minnaard et al., 1996; Abiusi, Sampietro et al., 2014).

**FIGURE 4:**
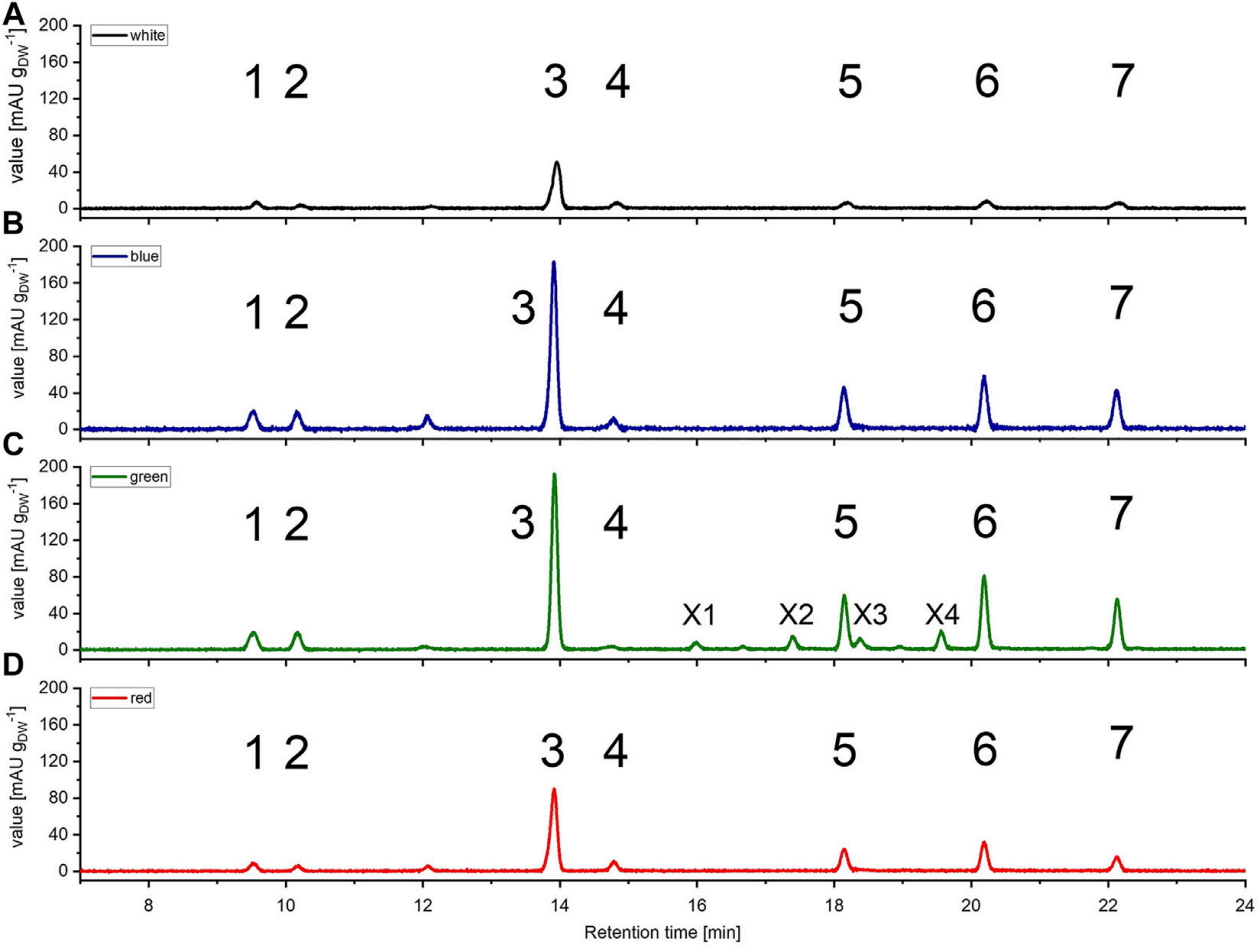
High pressure liquid chromatography (HPLC) spectra of *Picochlorum* sp. grown at **(A)** white, **(B)** blue, **(C)** green, and **(D)** red light, harvested at late exponential phase, at 150 μmol m^−2^ s^−1^ irradiation level. Peaks: 1 all-trans neoxanthin, 2 violaxanthin, 3 lutein, 4 canthaxanthin, 5 Chl *b*, 6 Chl *a*, 7 β-carotene, X1, X2, X3 and X4 unknown pigments (only occurring with green light). Measured on Kinetex 5 µm C8 column (150 mm * 4.6 mm), detection at λ = 450 nm. Known substances were identified by comparison to commercial standards.

Subsequently, pigments were extracted from biomass and separated *via* HPLC using samples harvested on day 11 of cultivation (late exponential growth phase) applying a 150 μmol m^−2^ s^−1^ illumination setup. All samples displayed pigment profiles, that were in agreement with previously reported profiles for *Picochlorum* sp.*,* prominently comprising lutein, Chl *a* and *b* and *β*-carotene. Additionally, minor signals for all-trans neoxanthin, violaxanthin, and canthaxanthin could be detected in samples illuminated with blue, red, and white light, respectively. All of the respective HPLC signals were identified in comparison to commercial standards (see Figure 4). Total carotenoid concentration ranged between 6.28–6.93 mg g_DW_^−1^ at white light and 5.92–6.02 mg g_DW_^−1^ at green light. The ratio of (Chl *b* + carotenoids)/Chl *a* that is considered to be related to the degree of light harvesting capability of PS II, varied between 1.01–1.79 with white illumination and 0.89-0.94 with green illumination. Moreover, the Chl *a*/*b* ratio was 2.01–2.80 and thus in the normal range of 1.5–4 for green algae (Wood 1979) indicating no noteworthy degradation during the pigment isolation procedure.

All pigment samples were subsequently normalized with respect to the amount of extracted biomass (see Figure 4). The normalized data indicated that samples generated with blue and green light illumination featured approximately three-fold higher pigment concentrations [mg_pigment_ g_DW_^−1^] compared to samples derived from white light illumination. An enhanced pigment content under blue light illumination has also been reported for the marine green algae *Tetraselmis suecica* (Abiusi, Sampietro et al., 2014).

Further, over the entire *Picochlorum* sp. data set the pigment distribution was similar, with the xanthophyll lutein being the most dominant pigment. With respect to chlorophylls, Chl *a* appeared to be dominant over Chl *b*, which is again consistent with literature data (da Silva Ferreira and Sant’Anna 2017). Likewise to the situation with blue and green light cultivation, pigment formation with red light illumination was also increased, compared to white light controls.

Notably, with green light LED illumination, additional, four additional peaks were detected reproducibly in HPLC analyses, which were absent under white, blue and red light color illumination conditions (see Figure 4). Hence, the additional signals were labeled as unknown pigment signals X1, X2, X3, and X4 respectively. Moreover, these additional pigment signals were detected at all green LED illumination intensities of 50–200 μmol m^−2^ s^−1^. Notably, the pigment signals were most prominent in samples harvested at the exponential growth phase.

### Shake Flask Experiments With Restrictive Illumination Spectra

To examine whether the formation of the extra pigments is connected to a certain outlier wavelength within the spectrum of the green LED–such as the blue-green or yellow-orange spectrum, a restrictive bandwidth reduction of the green LED was performed (530–610 nm) and compared to white light illumination. This bandwidth restriction of the green light LED was implemented *via* the addition of appropriate glass filter plates (long-pass filter and band pass filters). As different colored illumination modulates pigment formation in various microalgae (Lubián, Montero et al., 2000; Mohsenpour, Richards et al., 2012; Markou 2014; Schulze, Barreira et al., 2014; Glemser, Heining et al., 2016), blue, yellow or orange portions might also trigger other pigments to induce adaptation to the non-optimal, green light illumination.

**FIGURE 5:**
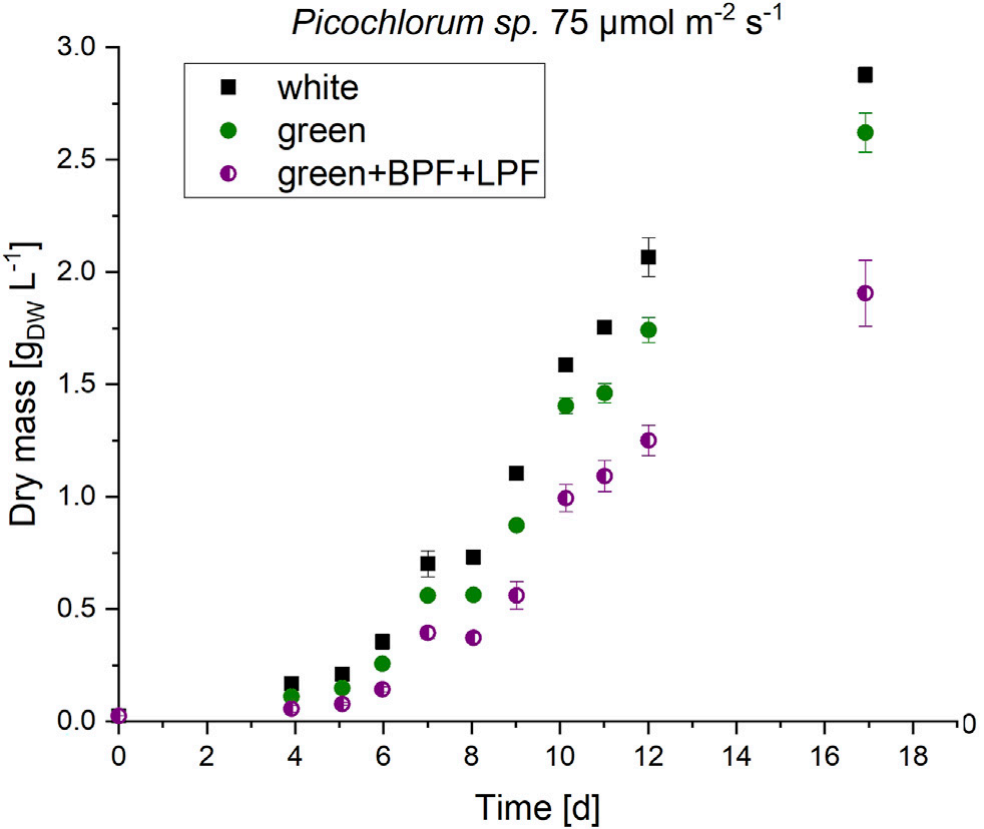
Biomass formation of *Picochlorum sp*. at 75 μmol m^−2^ s^−1^ irradiation with white, green and double-bandwidth diminishment green + BPF (band-pass filter B 13) +LPF (long-pass filter O 540). Experiments were carried out in triplicates.

Border illumination effects may be omitted and potential absorbance effects of the unknown pigments could be exposed in a follow-up experiment that applies double–bandwidth restrictions. A double-bandwidth restriction of the green LED to the central green wavelength at 510 nm, *via* a combination of the long-pass and band width filters, resulted in an illumination spectrum between 530 and 610 nm as shown in (see Figure 2D). Due to LED power limitations the total irradiation was limited to 75 μmol m^−2^ s^−1^ for the white, green and double–bandwidth restrictions settings. Under these low light conditions, slightly reduced growth was observed when compared to the control groups, grown in parallel under white and green light (see Figure 5). Nevertheless, this experimental setup showed *Picochlorum sp*. is able to grow under narrow green light illumination conditions. In the control with white light illumination the formation of unknown pigments was much lower than in samples subjected to green LED illumination without band-pass and/or long-pass wavelength restrictions (see Supplementary Figures S3, 4). Notably, the carotenoid pigment concentrations for neoxanthin, violaxanthin, zeaxanthin, lutein, canthaxanthin and β carotene did not differ significantly in the experiments with white and green illumination (see Supplementary Figure S5).

These experiments indicate, that the observed new pigment signals do not alter the absorbance range in *Picochlorum* sp. when illuminated with green light. In initial experiments, the observed growth under green LED illumination (see Figure 3) must therefore be caused by an absorbance overlap of the green LED spectra and general cell absorbance of *Picochlorum sp*. This correlates well with the reduced algae cell growth observed with the restricted narrow bandwidth and double-filter illumination setup (see Figure 5).

The formation of the unknown pigments is therefore triggered by the green illumination. However, the formation of the pigments does not directly correlate with absorbance or growth of the *Picochlorum* sp. cells. Absorbance analysis was performed with a cell suspension, therefore only the total absorption of the whole culture was detectable in these experiments (see Supplementary Figures S6, 7).

### Verification and Scale up for Pigment Isolation

**FIGURE 6:**
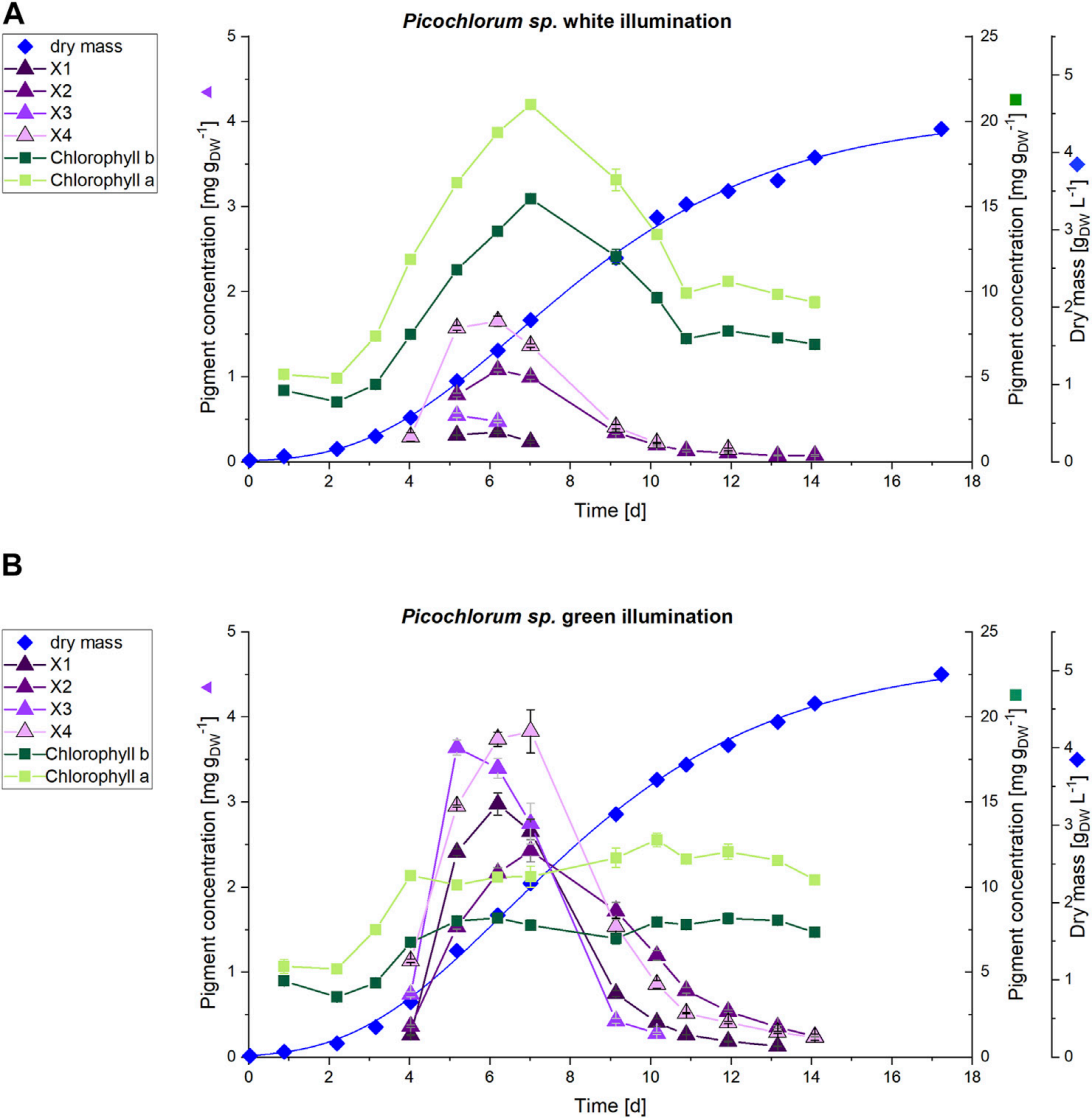
HPLC analysis of pigments (n = 3) of **(A)** white and **(B)** green illuminated *Picochlorum* sp. cultures at 150 μmol m^−2^ s^−1^. The scale for dry mass values (diamonds) is shown on the far right, while that for the concentrations of unknown pigments X1-4 (triangles) is on left side axis. Concentrations of chlorophyll *a* and *b* (squares) on the right side axis.

To evaluate whether the observation of unknown pigment formation during green light illumination is not a shake flask cultivation artifact, and to test conditions of higher cell density and comparable light intensities (150 μmol m^−2^ s^−1 ^PPFD) the experiments were repeated in commercial, controlled photobioreactors. Photobioreactor cultivation provided larger sample volumes, which allowed isolation of unknown pigments for downstream structural characterization. In that context, culture scale up to 3.7 L allowed for continuous sampling and pigment analysis and enabled sufficient sample volumes to apply high-resolution HPLC-MS and NMR for identification and structural characterization of unknown pigments. The stirred tank photobioreactor cultivations were carried out using the same green and white light illumination settings (white light as control) previously established in the shaker platform (without any filter plates). Although the cultivation resulted in similar growth rates, significant differences in the dry weight yields could be observed after a cultivation time of 17 days. Cultures under white illumination reached 4.2 g_DW_ L^−1^, whereas cultures with green light illumination yielded 4.9 g_DW_ L^−1^. The respective *Picochlorum *sp. cell growth and pigment formation obtained from cultures illuminated with green and white LEDs is shown in Figure 6.

The increased cultivation volume in the photobioreactor facilitated a daily pigment analysis for the first 2 weeks of cultivation. Subsequently, HPLC analysis indicated, that the unknown pigments are predominantly formed during the exponential growth phase, but decline towards the stationary phase. On day 7, the cultivation under white light illumination showed increased pigment concentrations with 21.0 mg g_DW_^−1^ and 15.5 mg g_DW_^−1^ of Chl *a* and Chl *b,* respectively. Thereafter, the chlorophyll content declined to a baseline level of 10 mg g_DW_^−1^ (Chl *a*) and 7.5 mg g_DW_^−1^ (Chl *b*) at the end of the cultivation.

In contrast, the Chl *a* and *b* contents in the green illuminated *Picochlorum* sp. cells remain constant at approximately 11 mg g_DW_^−1^ (Chl *a*) and 8 mg g_DW_^−1^ (Chl *b*) throughout the entire cultivation time, including the exponential growth phase. For the unknown pigments X1–X4 maximum production could be detected in the exponential growth phase (days 4–9). On day 6 the concentration of the unknown pigments was increased in the green illuminated setup by 8.5-fold (X1), 2-fold (X2), 7.1-fold (X3) and 2.3-fold (X4) to a maximum of around 3-4 mg g_DW_^−1^ (see Figure 6B). At green light, the sum of the concentrations of the unknown pigments X4 (3.7 mg g_DW_^−1^) and X3 (3.4 mg g_DW_^−1^), as well as that of Chl *a* (10.6 mg g_DW_^−1^) on day 6 almost equal the Chl *a* content of the white light illuminated cultivation control (19.4 mg g_DW_^−1^). With the sum of all extracted pigments obtained by the method used (see section 2.4) set to 100%, the combined sum of all unknown pigments X1-X4 rise from 8.7% at white light illumination to 34.9% at green light illumination at day 6.

A two-tailed *t*-test showed highly significant differences (*p* < 0.01) in pigment concentration for Chl *a*, Chl *b*, and the pigments X1-X4 during the exponential growth phase. The occurrence of the unknown pigments is limited to the exponential growth phase and declines in the stationary phase. This implies, that the formation of the unknown pigments provides *Picochlorum* sp. with a benefit for accelerated growth. The concentrations of the identified pigments (lutein, canthaxanthin, neoxanthin, violaxanthin, zeaxanthin and β-carotene) over the cultivation time is displayed in Supplementary Figure S5.

Absorbance spectra of X1 and X2 are nearly identical to that of Chl *b*, whereas those of pigments X3 and X4 are identical to that of Chl *a*. As X3 has a similar retention time as Chl *b* an identification *via* UV-Vis signal was difficult to measure and it could only be detected *via* tailing (indicated with the arrow in Supplementary Figure S8). Therefore, identification with further HPLC experiments with a higher peak resolution were performed.

### Structural Characterization of Pigments X1-X4 *via* HR HPLC and NMR

The newly identified pigments X1, X2, X3 and X4 formed with green light cultivation of *Picochlorum *sp. were each HPLC-purified from biomass samples generated during photobioreactor cultivation described in section 3.3. Purified samples were validated by UV-Vis absorbance spectrophotometry and subsequently subjected to HR-HPLC-MS and NMR analysis for further structural elucidation. High-resolution LC-MS enabled the determination of relative abundances *via* the m/z distributions and in comparison to simulated mass distributions of the pigments (shown in Supplementary Figure S9–14). The masses of the unknown pigments were recorded and correlated with those of Chl *a* and *b*. The observed MS spectral differences were consistent with additional C=C double bonds (+1 and +3) in the phytol side chain of Chl *a* and *b.* The data indicate that X3 and X4 are derivatives of Chl *a*, while X1 and X2 are derivatives of Chl *b*.

The HR-HPLC-MS data indicate double bond insertion in the phytol side chain of the chlorophyll porphyrin rings. This interpretation is also consistent with the lack of any observable changes in the UV-Vis absorption spectra of the unknown pigments and chlorophyll reference molecules, as the phytol chain is spectroscopically silent (Deisenhofer, Epp et al., 1995; Fiedor, Kania et al., 2008).

To further elucidate the structural properties of pigments X1-X4, a comprehensive NMR analysis was performed. The concentrations of purified chlorophylls and pigments X1-X4 allowed for ^1^H-^1^H-COSY and ^1^H-^13^C-HSQC analysis. These resulting spectra allowed a complete signal assignment of all chemical shifts for samples dissolved in d_6-._acetone. Subsequently, the ^1^H-chemical shifts of each sample could be correlated with relevant literature data (Abraham and Rowan 1991; Valverde and This 2008; Kobayashi, Akutsu et al., 2013) reported for chlorophylls (as shown in Supplementary Table S1). However, for small sample volumes the chemical shifts for unknown pigments were difficult to assign in the high-field region of the individual ^1^H spectra. Nonetheless, the peak intensity in the low-field region was sufficient to calculate the integrals of the peak areas (see P2 shown in Supplementary Figure S10). For the unassigned signals, which appeared to be similar to those of Chl *a* or *b*, an integration of the distinguished downfield signals (10, 5, 20 and 3^1^ for Chl *a*, and 7^1^, 5, 10, 20 and 3^1^ for Chl *b*) was conducted (see Supplementary Figure S1) for IUPAC numbering of chlorophylls). Comparison to the integrated signal area of the P2-shift allowed for the determination of additional double bonds in the respective chemical structures. Therefore, the NMR data were consistent with the previous HR-HPLC-MS analysis, thereby confirming that pigments X1-X4 feature additional double bonds in the phytol side chain of Chl *a* and *b*, respectively. Further, the NMR data allowed for the assignment of the exact positions of double bond insertions within the phytol moiety.

**FIGURE 7:**
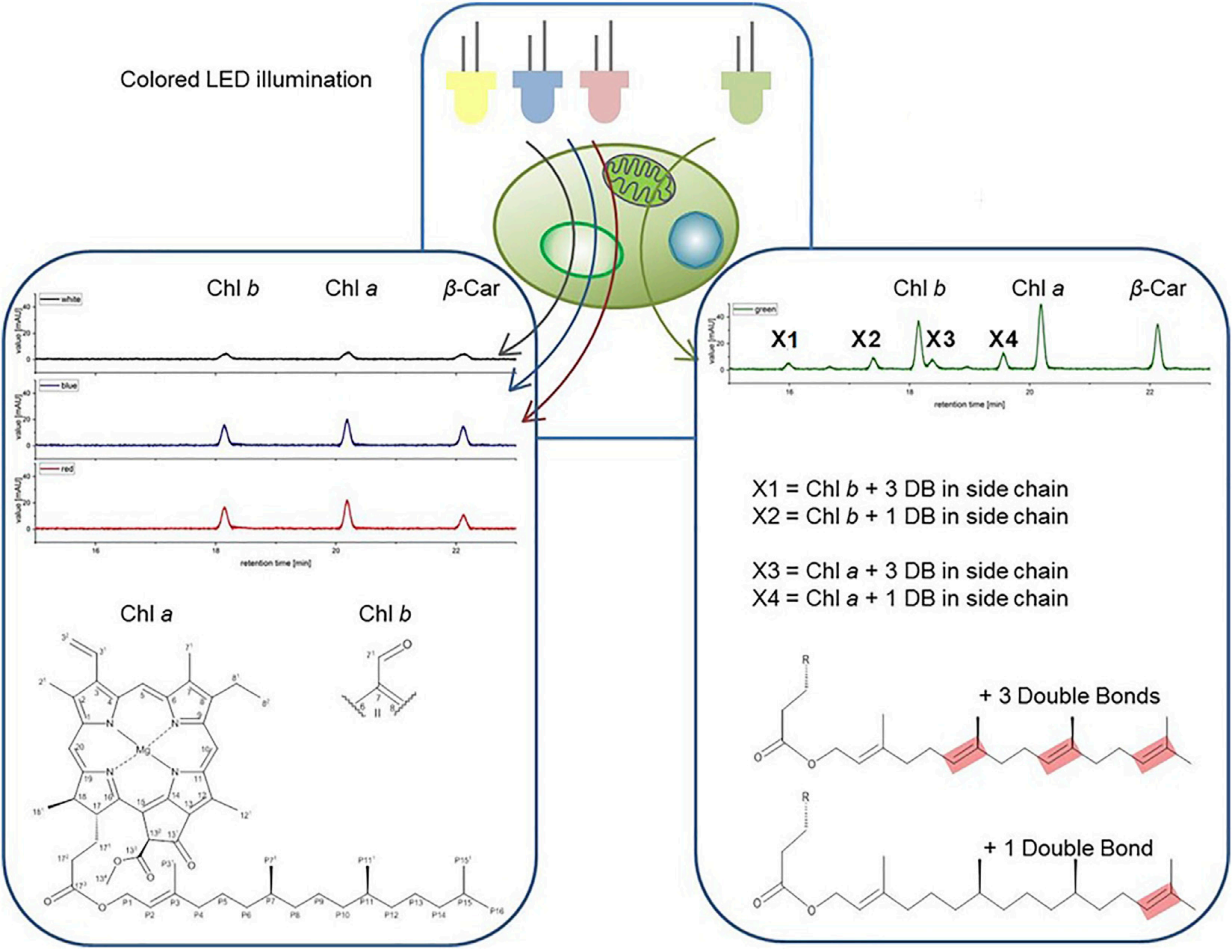
Structures of the pigments X1-X4 shown on right side. Green color LED illumination of *Picochlorum* sp. leads to the accumulation of additional pigments peaks in the exponential growth phase, identified as geranylgeranylated derivatives of Chl *a* and Chl *b* (shown on left side).

For bacteriochlorophyll (BChl) biosynthesis the chemical steps involved in the phytyl-group formation are reported (Mizoguchi, Harada et al., 2006). Specifically, the position of the C=C double bonds and their stepwise reduction, starting from the geranylgeranylpyrophosphate (GG; ∆2,6,10,14-Phytatetraenyl) structure, are well resolved. The transition from GG to Phy is a three-step process involving the reduction of double bonds. This process involves two chemical isomers of dihydrogeranylgeranyl (DHGG; ∆2,6,14-Phytatrienyl) and tetrahydrogeranyl-geranyl (THGG; ∆2,14-Phytadienyl) as key intermediates. The terminal step in this biosynthesis involves the reduction of the double bond between the P14 and P15 carbon atoms according to IUPAC nomenclature. The enzyme geranylgeranyl reductase is responsible for this reduction of phytyl chain double bonds. Indeed, high concentration increases of this enzyme were reported for several higher plants grown under monochromatic green light accompanied with the formation of high contents of GG and THGG (Materová, Sobotka et al., 2017). For identification GG, DHGG, THGG and Phy display differential ^1^H chemical shifts in the high-field region between 2.0 and 0.8 ppm (in a chloroform- *d* -pyridine-*d*_*5*_ solvent system). Unfortunately, the NMR signals obtained for the unknown pigments are too weak in this region for a substantial comparison to published data. Yet, this published pathway is a suitable base for assigning the position of the observed double bonds. Our data indicated that C=C bonds are indeed positioned between P^6^-P^7^, P^10^-P^11^ and P^14^-P^15^ in the unknown pigments X1 and X3 and thus are identical to GG (with three extra double bonds) corresponding to the actual MH^+^ ion [m/z] (see Table 4) for chlorophyll *b* esterified with GG (X1) and chlorophyll *a* with GG (X3). Moreover, regarding pigments X2 and X4 the double bonds are at position P^14^-P^15^, respectively (see Figure 7), corresponding to the actual MH^+^ ion [m/z] (see Table 4) from chlorophyll *b* esterified with THGG (X2) and chlorophyll *a* with THGG (X4). These results are in accordance to findings from purple photosynthetic bacteria (e.g., *Rhodopseudomonas palustris* (Mizoguchi, Harada et al., 2006; Mizoguchi, Isaji et al., 2015), cyanobacterial mutants with an inactive of geranylgeranyl reductase gene (ChlP) (Shpilyov, Zinchenko et al., 2005), diatoms (*Chaetoceros calcitrans*; (Mizoguchi, Isaji et al., 2017)) and higher plants exposed to green light (Materová, Sobotka et al., 2017; Karlický, Materová et al., 2021). Chlorophylls esterified with DHGG could not be detected in this study.

**TABLE 4:** Identification of chlorophylls by high resolution high pressure liquid chromatography-mass spectrometry (HPLC-MS) analysis of chlorophyll (Chl) *a* and *b* and unknown pigments X1-X4.

Pigment	Simulated matching MH^+^ ion formula	Simulated matching MH^+^ ion [m/z]	Actual MH^+^ ion [m/z]	Conformity value [ppm]	Potential construct
Chl *a*	C_55_H_73_O_5_N_4_Mg	893.54259	893.54219	-0.44315	Chlorophyll *a*
X4	C_55_H_71_O_5_N_4_Mg	891.52694	891.52644	-0.55846	Chl *a* + 1 DB
X3	C_55_H_67_O_5_N_4_Mg	887.49564	887.49532	-0.35664	Chl *a* + 3 DB
Chl *b*	C_55_H_71_O_6_N_4_Mg	907.52185	907.52182	-0.04237	Chlorophyll *b*
X2	C_55_H_69_O_6_N_4_Mg	905.50620	905.50598	-0.24781	Chl *b* + 1 DB
X1	C_55_H_65_O_6_N_4_Mg	901.47490	901.47479	-0.12793	Chl *b* + 3 DB

Chlorophylls with esterified GG, DHGG, and THGG were reported from higher plants during greening processes (early phase of de-etiolation), when chloroplast development is extensively stimulated (Schoefs 2000), and thus were suggested as biosynthetic precursors for phytylated chlorophylls. In shake flask experiments the massive formation of chlorophylls esterified with GG and THGG was exclusively induced by green light and could not been detected under other light regimes. Moreover, the highest synthesis of these pigments was during the exponential growth phase. In addition, since pheophytin, a well-known marker of chlorophyll degradation was absent in all samples, we propose that the change in the esterified chlorophylls occurs during pigment synthesis rather than during its degradation. These observations indicate an active induced physiological mechanism caused by acclimation to monochromatic green light suggesting that these chlorophylls are not just biosynthetic precursors but photosynthetically active pigments.

Although the phytyl residue is not considered to be involved in the spectral properties of chlorophyll, it indirectly affects the electronic absorption, as it interacts with the hydrophobic thylakoid membrane environment (Fiedor, Stasiek et al., 2003). The insertion of additional C=C double bonds in the phytyl moiety could therefore result in higher mobility of the chromophore in the thylakoid membrane, under green light illumination. As the two photosystems are finely tuned to respond to changing light qualities, phosphorylation of the LHCII antenna complexes have also been observed in *Arabidopsis thaliana* (Wunder, Xu et al., 2013; Longoni, Douchi et al., 2015) and *Chlamydomonas sp*. (Bellafiore, Barneche et al., 2005; Frenkel, Bellafiore et al., 2007; Goldschmidt-Clermont and Bassi 2015). To that end, the antenna complexes in the photosystem can be adapted for an optimum response to changing light qualities and quantities by repositioning and rearrangement of antenna chromophores. The observed formation of new *Picochlorum* sp. pigments in the exponential growth phase under green light illumination could therefore be caused by a similar regulatory system. The additional double bonds in the phytol side chain allow the chlorophylls higher mobility within the hydrophobic membranes. With this enhanced mobility, a better regulation between the photosystems could be arranged in order to adapt to the unusual green light illumination.

Moreover, the interaction with other proteins in the thylakoid membrane would also be affected through the altered side-chain and have a positive impact on the structures of the antenna chromophores within the light-harvesting complex of *Picochlorum sp*. An involvement of the light harvesting complex (LHC) was found in purple photosynthetic bacteria, preferentially in core complexes over the peripheral antennas (Mizoguchi, Isaji et al., 2015), and recently also confirmed in higher plants illuminated with monochromatic green light (Karlický, Materová et al., 2021). In *A. thaliana* geranylgeranylated chlorophylls were more abundant in light-harvesting complex II (LHCII) and less prominent in supercomplexes of photosystem II (PSII). Moreover, the accumulation of geranylgeranylated chlorophylls hampered the formation of PSII and PSI super- and megacomplexes in the thylakoid membranes as well as their assembly into chiral macrodomains (Karlický, Materová et al., 2021). A reduced stability of photosynthetic pigment-protein complexes (PPC) assembled with geranylgeranylated chlorophylls was also found in cyanobacterial mutants lacking geranylgeranyl reductase (Shpilyov, Zinchenko et al., 2005). Also the temperature stability of pigment-protein complexes in higher plants was lowered, especially that of LHCII trimers, which led to their monomerization and an anomaly in the photoprotective mechanism of non-photochemical quenching (Karlický, Materová et al., 2021). We therefore suggest a similar role of geranylgeranylated chlorophylls for acclimation processes in *Picochlorum sp*. because the formation of these chlorophylls with esterified GG and THGG can be found from photosynthetic bacteria to algae to higher plants and therefore represent a conservative process in evolutionary terms.

In all studies on the role of geranylgeranylated chlorophylls adverse effects on photosynthetic apparatus have been determined and are concomitant with reduced photosynthetic growth. Interestingly, *Picochlorum sp*. acclimated to green light with comparable or even better growth rates under different experimental designs despite lower contents of Chl *a*+*b* and negative impacts on LHC and stability. Thus, this is the first time, that green light induced geranylgeranylated chlorophylls have been found in algae and it is the first time that physiological advantages are documented during the formation of these pigments.

### Conclusion

This study demonstrates that the marine eukaryotic green algae *Picochlorum* sp. can be efficiently cultivated under sole green LED light illumination, reaching the same biomass yields as controls grown under red or white light respectively. Interestingly, cultivation with green light is associated with a slow adaptation phase, which, dependent on the cultivation conditions, can extend up to 6 days. HPLC analysis of the extracted pigment profile indicated, that this lag phase was associated with the formation of unusual photosynthetic pigments, which were observed in both LED-illuminated shake flasks and controlled stirred-tank photobioreactors. High resolution LC-MS and NMR analyses allowed for the identification of pigments as derivatives of Chl *a* and *b,* esterified with GG and THGG, featuring up to three additional C=C double bond insertions in the phytol side chain. When *Picochlorum* sp. is cultivated with green LED light, these chlorophyll derivatives appear to be beneficial, particularly in the exponential growth phase, while their concentrations decline gradually towards the stationary phase. At present, there is no data on the exact impact of green light on metabolic pathways that are involved in chlorophyll synthesis. Especially the regulation of respective enzymes in conjunction with different light spectra needs further investigation. Geranylgeranylated chlorophylls which are produced under green light illumination might be photosynthetically active pigments (Mizoguchi, Isaji et al., 2017). However, this statement cannot be sufficiently verified with the currently available data. To our knowledge, this is the first time that the green light induction of these chlorophyll derivatives are described for *Picochlorum* sp. or any algae. Our experimental setup showed a clear light color dependence for the formation of the identified chlorophyll derivatives, indicating a high flexibility in the chlorophyll biosynthesis pathway of *Picochlorum sp*. Changes in spectral composition can be matched by *Picochlorum* sp. by alternation in the pigment variety and absorption capabilities. These findings, in combination with obtained high biomass productivity, suggest that green light illumination can be applied in optimizing bioreactor illumination setups. To that end, irradiation wavelength and intensity are challenging parameters in modern photobioreactor design. The utilization of additional green LED light may intensify light penetration in the photobioreactor at high cell densities, which are observed during the exponential growth phase. This may prevent light limitation in the center of the photobioreactor, thereby resulting in increased biomass productivity. Moreover, green light might prevent contamination from other green algae, because they are outcompeted by higher growth rates of *Picochlorum sp*., a process also used as isolation technique of cyanobacteria from environmental samples because their LHC with phycobilisoms are able to utilize green light by a process called complementary chromatic adaptation (Tandeau de Marsac 1977). More than 80 years after the initial structure determination of chlorophylls by Hans Fischer (Fischer 1937) there are still variants of these enigmatic pigment molecules to be discovered supporting acclimation mechanisms in different organisms. Moreover, our data demonstrates, that expanding knowledge in fundamental pigment research is essential to improve cultivation technologies for phototrophic organisms that can provide sustainable food and chemicals for a circular bioeconomy.

## Data Availability

The original contributions presented in the study are included in the article/Supplementary Material, further inquiries can be directed to the corresponding author.

## References

[B1] Abiusi F. Sampietro G. Marturano G. Biondi N. Rodolfi L. D'Ottavio M. (2014). Growth, Photosynthetic Efficiency, and Biochemical Composition ofTetraselmis suecicaF&M-M33 Grown with LEDs of Different Colors. Biotechnol. Bioeng. 111 (5), 956–964. 10.1002/bit.25014 23904253

[B2] Abraham R. J. Rowan A. (1991). Nuclear Magnetic Resonance Spectroscopy of Chlorophyll.

[B3] An J. Gao F. Ma Q. Xiang Y. Ren D. Lu J. (2017). Screening for Enhanced Astaxanthin Accumulation Among Spirulina Platensis Mutants Generated by Atmospheric and Room Temperature Plasmas. Algal Res. 25, 464–472. 10.1016/j.algal.2017.06.006

[B4] Bellafiore S. Barneche F. Peltier G. Rochaix J.-D. (2005). State Transitions and Light Adaptation Require Chloroplast Thylakoid Protein Kinase STN7. Nature 433 (7028), 892–895. 10.1038/nature03286 15729347

[B5] Bleakley S. Hayes M. (2017). Algal Proteins: Extraction, Application, and Challenges Concerning Production. Foods 6 (5), 33. 10.3390/foods6050033 PMC5447909 28445408

[B6] Boussiba S. Vonshak A. Cohen Z. Avissar Y. Richmond A. (1987). Lipid and Biomass Production by the Halotolerant Microalga Nannochloropsis salina. Biomass 12 (1), 37–47. 10.1016/0144-4565(87)90006-0

[B7] Brzezowski P. Richter A. S. Grimm B. (2015). Regulation and Function of Tetrapyrrole Biosynthesis in Plants and Algae. Biochim. Biophys. Acta (Bba) - Bioenerg. 1847 (9), 968–985. 10.1016/j.bbabio.2015.05.007 25979235

[B8] Cao J. Li F. Li Y. Chen H. Liao X. Zhang Y. (2021). Hydrophobic Interaction Driving the Binding of Soybean Protein Isolate and Chlorophyll: Improvements to the thermal Stability of Chlorophyll. Food Hydrocolloids 113, 106465. 10.1016/j.foodhyd.2020.106465

[B9] Chaves O. Amorim A. Castro L. Sant’Anna C. de Oliveira M. Cesarin-Sobrinho D. (2015). Fluorescence and Docking Studies of the Interaction between Human Serum Albumin and Pheophytin. Molecules 20, 19526–19539. 10.3390/molecules201019526 26516829 PMC6332261

[B10] Chukhutsina V. Bersanini L. Aro E. M. van Amerongen H. (2015). Cyanobacterial Light-Harvesting Phycobilisomes Uncouple from Photosystem I during Dark-To-Light Transitions. Sci. Rep. 5 (1), 1–10. 10.1038/srep14193 PMC4585685 26388233

[B11] da Silva Ferreira V. Sant'Anna C. (2017). Impact of Culture Conditions on the Chlorophyll Content of Microalgae for Biotechnological Applications. World J. Microbiol. Biotechnol. 33 (1), 20–28. 10.1007/s11274-016-2181-6 27909993

[B12] Das P. Lei W. Aziz S. S. Obbard J. P. (2011). Enhanced Algae Growth in Both Phototrophic and Mixotrophic Culture under Blue Light. Bioresour. Technology 102 (4), 3883–3887. 10.1016/j.biortech.2010.11.102 21183340

[B13] Deisenhofer J. Epp O. Sinning I. Michel H. (1995). Crystallographic Refinement at 2.3 Å Resolution and Refined Model of the Photosynthetic Reaction Centre fromRhodopseudomonas Viridis. J. Mol. Biol. 246 (3), 429–457. 10.1006/jmbi.1994.0097 7877166

[B14] Del Campo J. Moreno J. Rodriguez H. Vargas M. A. Rivas J. n. Guerrero M. G. (2000). Carotenoid Content of Chlorophycean Microalgae: Factors Determining Lutein Accumulation in Muriellopsis Sp. (Chlorophyta). J. Biotechnol. 76 (1), 51–59. 10.1016/s0168-1656(99)00178-9 10784296

[B15] Fiedor L. Kania A. Myśliwa-Kurdziel B. Orzeł Ł. Stochel G. (2008). Understanding Chlorophylls: central Magnesium Ion and Phytyl as Structural Determinants. Biochim. Biophys. Acta (Bba) - Bioenerg. 1777 (12), 1491–1500. 10.1016/j.bbabio.2008.09.005 18848915

[B16] Fiedor L. Stasiek M. Myśliwa-Kurdziel B. Strzałka K. (2003). Phytol as One of the Determinants of Chlorophyll Interactions in Solution. Photosynthesis Res. 78 (1), 47–57. 10.1023/a:1026042005536 16245063

[B17] Fischer H. (1937). Chlorophyll. Chem. Rev. 20 (1), 41–68. 10.1021/cr60065a002

[B18] Fisher T. Minnaard J. Dubinsky Z. (1996). Photoacclimation in the marine Alga Nannochloropsis Sp. (Eustigmatophyte): a Kinetic Study. J. Plankton Res. 18 (10), 1797–1818. 10.1093/plankt/18.10.1797

[B19] Foflonker F. Price D. C. Qiu H. Palenik B. Wang S. Bhattacharya D. (2015). Genome of the Halotolerant green algaPicochlorumsp. Reveals Strategies for Thriving under Fluctuating Environmental Conditions. Environ. Microbiol. 17 (2), 412–426. 10.1111/1462-2920.12541 24965277

[B20] Franco M. (2014). Batch Cultivation of Microalgae in the Labfors 5 Lux Photobioreactor with LED Flat Panel Option. InforsHT Appl. note, 1–6.

[B21] Franz A. K. Danielewicz M. A. Wong D. M. Anderson L. A. Boothe J. R. (2013). Phenotypic Screening with Oleaginous Microalgae Reveals Modulators of Lipid Productivity. ACS Chem. Biol. 8 (5), 1053–1062. 10.1021/cb300573r 23521767

[B22] Frenkel M. Bellafiore S. Rochaix J. D. Jansson S. (2007). Hierarchy Amongst Photosynthetic Acclimation Responses for Plant Fitness. Physiologia Plantarum 129 (2), 455–459.

[B23] Futurled (2022). LED Shaker Prototype. Berlin: Ger. Available at: https://futureled.de/ (Accessed Feb 1st, 2022).

[B24] Glemser M. Heining M. Schmidt J. Becker A. Garbe D. Buchholz R. (2016). Application of Light-Emitting Diodes (LEDs) in Cultivation of Phototrophic Microalgae: Current State and Perspectives. Appl. Microbiol. Biotechnol. 100 (3), 1077–1088. 10.1007/s00253-015-7144-6 26590582

[B25] Goldschmidt-Clermont M. Bassi R. (2015). Sharing Light between Two Photosystems: Mechanism of State Transitions. Curr. Opin. Plant Biol. 25, 71–78. 10.1016/j.pbi.2015.04.009 26002067

[B26] Goswami R. K. Agrawal K. Verma P. (2021). Phycoremediation of Nitrogen and Phosphate from Wastewater Using Picochlorum sp. A Tenable Approach. J. Basic Microbiol. 62, 3–4. 10.1002/jobm.202100277 34312905

[B27] Infors (2017). Infors AG, Labfors 5 Lux. Available at: https://www.infors-ht.com/index.php/de/produkte/bioreaktoren/tischbioreaktoren/labfors-5-lux (Accessed June 15th, 2017).

[B28] Kahm M. Hasenbrink G. Lichtenberg-Frate H. Ludwig J. Kschischo M. (2010). Grofit: Fitting Biological Growth Curves. Nat. Prec, 1. 10.1038/npre.2010.4508.1

[B29] Karlický V. Materová Z. K. Kurasová I. Nezval J. Štroch M. Garab G. (2021). Accumulation of Geranylgeranylated Chlorophylls in the Pigment-Protein Complexes of *Arabidopsis thaliana* Acclimated to green Light: Effects on the Organization of Light-Harvesting Complex II and Photosystem II Functions. Photosynthesis Res. 149, 1–20. 10.1007/s11120-021-00827-1 PMC8382614 33948813

[B30] Kehoe D. M. Grossman A. R. (1994). Complementary Chromatic Adaptation: Photoperception to Gene Regulation. Semin. Cel. Biol. 5, 303–313. 10.1006/scel.1994.1037 7881070

[B31] Kobayashi M. Akutsu S. Fujinuma D. Furukawa H. Komatsu H. Hotota Y. (2013). Physicochemical Properties of Chlorophylls in Oxygenic Photosynthesis—Succession of Co-factors from Anoxygenic to Oxygenic Photosynthesis. Photosynthesis, 47–90.

[B32] LaserComponents (2022). Laser Components GmbH, Colored Glass Filter. Available at: https://www.lasercomponents.com/de-en/product/colored-glass/ (Accessed Feb 1st, 2022).

[B33] Lee J.-Y. Seo S.-H. Ahn C.-Y. Lee C. S. An K.-G. Srivastava A. (2019). Green Light as Supplementary Light for Enhancing Biomass Production of Ettlia Sp. And Preventing Population Invasion from Other Microalgae. J. Appl. Phycol 31 (4), 2207–2215. 10.1007/s10811-019-1737-x

[B34] Li Y. Liu J. (2020). Analysis of Light Absorption and Photosynthetic Activity by Isochrysis Galbana under Different Light Qualities. Aquac. Res. 51 (7), 2893–2902. 10.1111/are.14628

[B35] Liu J. Chen J. Chen Z. Qin S. Huang Q. (2016). Isolation and Characterization of Astaxanthin-Hyperproducing Mutants of Haematococcus pluvialis (Chlorophyceae) Produced by Dielectric Barrier Discharge Plasma. Phycologia 55 (6), 650–658. 10.2216/16-14.1

[B36] Longoni P. Douchi D. Cariti F. Fucile G. Goldschmidt-Clermont M. (2015). Phosphorylation of the Lhcb2 Isoform of Light Harvesting Complex II Is central to State Transitions 2. Plant Physiol. 169 (6), 2874–2883. 10.1104/pp.15.01498 26438789 PMC4677923

[B37] Lorenz R. T. Cysewski G. R. (2000). Commercial Potential for Haematococcus Microalgae as a Natural Source of Astaxanthin. Trends Biotechnology 18 (4), 160–167. 10.1016/s0167-7799(00)01433-5 10740262

[B38] Lubián L. M. Montero O. Moreno-Garrido I. Huertas I. E. Sobrino C. González-del Valle M. (2000). Nannochloropsis (Eustigmatophyceae) as Source of Commercially Valuable Pigments. J. Appl. Phycology 12 (3), 249–255.

[B39] Markou G. (2014). Effect of Various Colors of Light-Emitting Diodes (LEDs) on the Biomass Composition of Arthrospira Platensis Cultivated in Semi-continuous Mode. Appl. Biochem. Biotechnol. 172 (5), 2758–2768. 10.1007/s12010-014-0727-3 24435766

[B40] Masojıdek J. Koblızek M. Torzillo G. (2004). 2 Photosynthesis in Microalgae. Handbook microalgal Cult. Biotechnol. Appl. phycology 20. 10.1002/9781118567166.ch2

[B41] Mata T. M. Martins A. A. Caetano N. S. (2010). Microalgae for Biodiesel Production and Other Applications: a Review. Renew. Sustain. Energ. Rev. 14 (1), 217–232. 10.1016/j.rser.2009.07.020

[B42] Materová Z. Sobotka R. Zdvihalová B. Oravec M. Nezval J. Karlický V. (2017). Monochromatic green Light Induces an Aberrant Accumulation of Geranylgeranyled Chlorophylls in Plants. Plant Physiol. Biochem. 116, 48–56. 28527413 10.1016/j.plaphy.2017.05.002

[B43] Mattos E. R. Singh M. Cabrera M. L. Das K. C. (2015). Enhancement of Biomass Production in Scenedesmus Bijuga High-Density Culture Using Weakly Absorbed green Light. Biomass and Bioenergy 81, 473–478. 10.1016/j.biombioe.2015.07.029

[B44] Merritt J. E. Loening K. L. (1980). IUPAC-IUB Joint Commission on Biochemical Nomenclature (JCBN) Nomenclature of Tetrapyrroles Recommendations 1978. Eur. J. Biochem. 108 (1), 1–30. 10.1111/j.1432-1033.1980.tb04691.x 7408840

[B45] Mizoguchi T. Harada J. Tamiaki H. (2006). Structural Determination of Dihydro- and Tetrahydrogeranylgeranyl Groups at the 17-propionate of Bacteriochlorophylls-A. FEBS Lett. 580 (28-29), 6644–6648. 10.1016/j.febslet.2006.11.020 17123518

[B46] Mizoguchi T. Isaji M. Harada J. Tsukatani Y. Tamiaki H. (2015). The 17-propionate Esterifying Variants of Bacteriochlorophyll-A and Bacteriopheophytin-A in Purple Photosynthetic Bacteria. J. Photochem. Photobiol. B: Biol. 142, 244–249. 10.1016/j.jphotobiol.2014.12.013 25559490

[B47] Mizoguchi T. Isaji M. Yamano N. Harada J. Fujii R. Tamiaki H. (2017). Molecular Structures and Functions of Chlorophylls-A Esterified with Geranylgeranyl, Dihydrogeranylgeranyl, and Tetrahydrogeranylgeranyl Groups at the 17-propionate Residue in a Diatom, Chaetoceros Calcitrans. Biochemistry 56 (28), 3682–3688. 10.1021/acs.biochem.7b00381 28627163

[B48] Mohsenpour S. F. Richards B. Willoughby N. (2012). Spectral Conversion of Light for Enhanced Microalgae Growth Rates and Photosynthetic Pigment Production. Bioresour. Technol. 125, 75–81. 10.1016/j.biortech.2012.08.072 23023239

[B49] Mohsenpour S. F. Willoughby N. (2013). Luminescent Photobioreactor Design for Improved Algal Growth and Photosynthetic Pigment Production through Spectral Conversion of Light. Bioresour. Technol. 142, 147–153. 10.1016/j.biortech.2013.05.024 23735796

[B50] Nakamura Y. Li-Beisson Y. (2016). Lipids in Plant and Algae Development. Springer. 10.1007/978-3-319-25979-6

[B51] Nürnberg D. J. Morton J. Santabarbara S. Telfer A. Joliot P. Antonaru L. A. (2018). Photochemistry beyond the Red Limit in Chlorophyll F–Containing Photosystems. Science 360 (6394), 1210–1213. 29903971 10.1126/science.aar8313

[B52] Olivieri G. Salatino P. Marzocchella A. (2014). Advances in Photobioreactors for Intensive Microalgal Production: Configurations, Operating Strategies and Applications. J. Chem. Technol. Biotechnol. 89 (2), 178–195. 10.1002/jctb.4218

[B53] Ooms M. D. Dinh C. T. Sargent E. H. Sinton D. (2016). Photon Management for Augmented Photosynthesis. Nat. Commun. 7 (1), 12699–12713. 10.1038/ncomms12699 27581187 PMC5025804

[B54] Ooms M. D. Graham P. J. Nguyen B. Sargent E. H. Sinton D. (2017). Light Dilution via Wavelength Management for Efficient High-Density Photobioreactors. Biotechnol. Bioeng. 114 (6), 1160–1169. 10.1002/bit.26261 28165134

[B55] Paliwal C. Jutur P. P. (2021). Dynamic Allocation of Carbon Flux Triggered by Task-specific Chemicals Is an Effective Non-gene Disruptive Strategy for Sustainable and Cost-Effective Algal Biorefineries. Chem. Eng. J. 418, 129413. 10.1016/j.cej.2021.129413

[B56] Roy S. Llewellyn C. A. Egeland E. S. Johnsen G. (2011). Phytoplankton Pigments: Characterization, Chemotaxonomy and Applications in Oceanography. Cambridge University Press, 890.

[B57] Sarrafzadeh M. H. La H.-J. Lee J.-Y. Cho D.-H. Shin S.-Y. Kim W.-J. (2015). Microalgae Biomass Quantification by Digital Image Processing and RGB Color Analysis. J. Appl. Phycol 27 (1), 205–209. 10.1007/s10811-014-0285-7

[B58] Schoefs B. (2000). The Light-dependent and Light-independent Reduction of Protochlorophyllide a to Chlorophyllide a. Photosynthetica 36 (4), 481–496.

[B59] Schulze P. S. C. Barreira L. A. Pereira H. G. C. Perales J. A. Varela J. C. S. (2014). Light Emitting Diodes (LEDs) Applied to Microalgal Production. Trends Biotechnology 32 (8), 422–430. 10.1016/j.tibtech.2014.06.001 25012573

[B60] Shpilyov A. V. Zinchenko V. V. Shestakov S. V. Grimm B. Lokstein H. (2005). Inactivation of the Geranylgeranyl Reductase (ChlP) Gene in the Cyanobacterium Synechocystis Sp. PCC 6803. Biochim. Biophys. Acta (Bba) - Bioenerg. 1706 (3), 195–203. 10.1016/j.bbabio.2004.11.001 15694347

[B61] Singh N. K. Sonani R. R. Rastogi R. P. Madamwar D. (2015). The Phycobilisomes: an Early Requisite for Efficient Photosynthesis in Cyanobacteria. EXCLI J. 14, 268–289. 10.17179/excli2014-723 26417362 PMC4553884

[B62] Singh P. K. (1975). Photoreactivation of UV-Irradiated Blue-green Algae and Algal Virus LPP-1. Arch. Microbiol. 103 (1), 297–302. 10.1007/bf00436364 807175

[B63] Smith H. L. McAusland L. Murchie E. H. (2017). Don't Ignore the green Light: Exploring Diverse Roles in Plant Processes. J. Exp. Bot. 68 (9), 2099–2110. 10.1093/jxb/erx098 28575474

[B64] Tandeau de Marsac N. (1977). Occurrence and Nature of Chromatic Adaptation in Cyanobacteria. J. Bacteriol. 130 (1), 82–91. 10.1128/jb.130.1.82-91.1977 856789 PMC235176

[B65] Toole C. M. Allnutt F. C. T. (2003). Red, Cryptomonad and Glaucocystophyte Algal Phycobiliproteins. Photosynthesis in algae 22-24, 305–334. 10.1007/978-94-007-1038-2_14

[B66] Vadiveloo A. Moheimani N. R. Cosgrove J. J. Bahri P. A. Parlevliet D. (2015). Effect of Different Light Spectra on the Growth and Productivity of Acclimated Nannochloropsis Sp. (Eustigmatophyceae). Algal Res. 8, 121–127. 10.1016/j.algal.2015.02.001

[B67] Valverde J. This H. (2008). 1H NMR Quantitative Determination of Photosynthetic Pigments from green Beans (Phaseolus vulgaris L.). J. Agric. Food Chem. 56 (2), 314–320. 10.1021/jf070277j 18081249

[B68] Van Heukelem L. Thomas C. S. (2001). Computer-assisted High-Performance Liquid Chromatography Method Development with Applications to the Isolation and Analysis of Phytoplankton Pigments. J. Chromatogr. A 910 (1), 31–49. 10.1016/s0378-4347(00)00603-4 11263574

[B69] Von Wettstein D. Gough S. Kannangara C. G. (1995). Chlorophyll Biosynthesis. The Plant Cell 7 (7), 1039. 10.2307/3870056 12242396 PMC160907

[B70] Walter A. Carvalho J. C. d. Soccol V. T. Faria A. B. B. d. Ghiggi V. Soccol C. R. (2011). Study of Phycocyanin Production from Spirulina Platensis under Different Light Spectra. Braz. Arch. Biol. Technol. 54, 675–682. 10.1590/s1516-89132011000400005

[B71] Wood A. M. (1979). Chlorophyll A:b Ratios IN Marine Planktonic Algae1. J. Phycology 15 (3), 330–332. 10.1111/j.0022-3646.1979.00330.x

[B72] Woodward R. B. (1960). Totalsynthese des chlorophylls. Angew. Chem. 72 (18), 651–662. 10.1002/ange.19600721803

[B73] Woortman D. V. Fuchs T. Striegel L. Fuchs M. Weber N. Brück T. B. (2020). Microalgae a superior Source of Folates: Quantification of Folates in Halophile Microalgae by Stable Isotope Dilution Assay. Front. Bioeng. Biotechnol. 7, 481. 10.3389/fbioe.2019.00481 32039182 PMC6985443

[B74] Wunder T. Xu W. Liu Q. Wanner G. Leister D. Pribil M. (2013). The Major Thylakoid Protein Kinases STN7 and STN8 Revisited: Effects of Altered STN8 Levels and Regulatory Specificities of the STN Kinases. Front. Plant Sci. 4, 417. 10.3389/fpls.2013.00417 24151498 PMC3801152

[B75] Yan C. Zhang L. Luo X. Zheng Z. (2013). Effects of Various LED Light Wavelengths and Intensities on the Performance of Purifying Synthetic Domestic Sewage by Microalgae at Different Influent C/N Ratios. Ecol. Eng. 51, 24–32. 10.1016/j.ecoleng.2012.12.051

[B76] Yang F. Xiang W. Sun X. Wu H. Li T. Long L. (2014). A Novel Lipid Extraction Method from Wet Microalga Picochlorum Sp. At Room Temperature. Mar. Drugs 12 (3), 1258–1270. 10.3390/md12031258 24663114 PMC3967208

