# Supplementary figures and images for: Efficient Green Light Acclimation of the Green Algae Picochlorum sp. Triggering Geranylgeranylated Chlorophylls
Source: Front Bioeng Biotechnol. 2022 Apr 28;10:885977. doi: 10.3389/fbioe.2022.885977 (PMC9095919; doi:10.3389/fbioe.2022.885977)

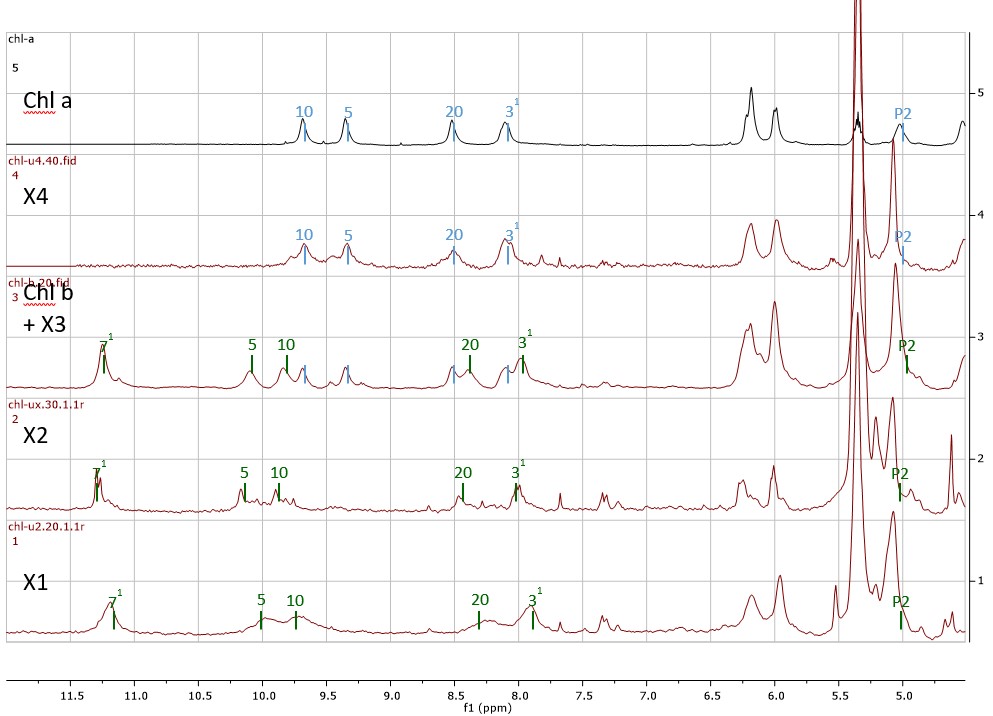

Supplement: Supplementary file 1 [file Image15.JPEG]

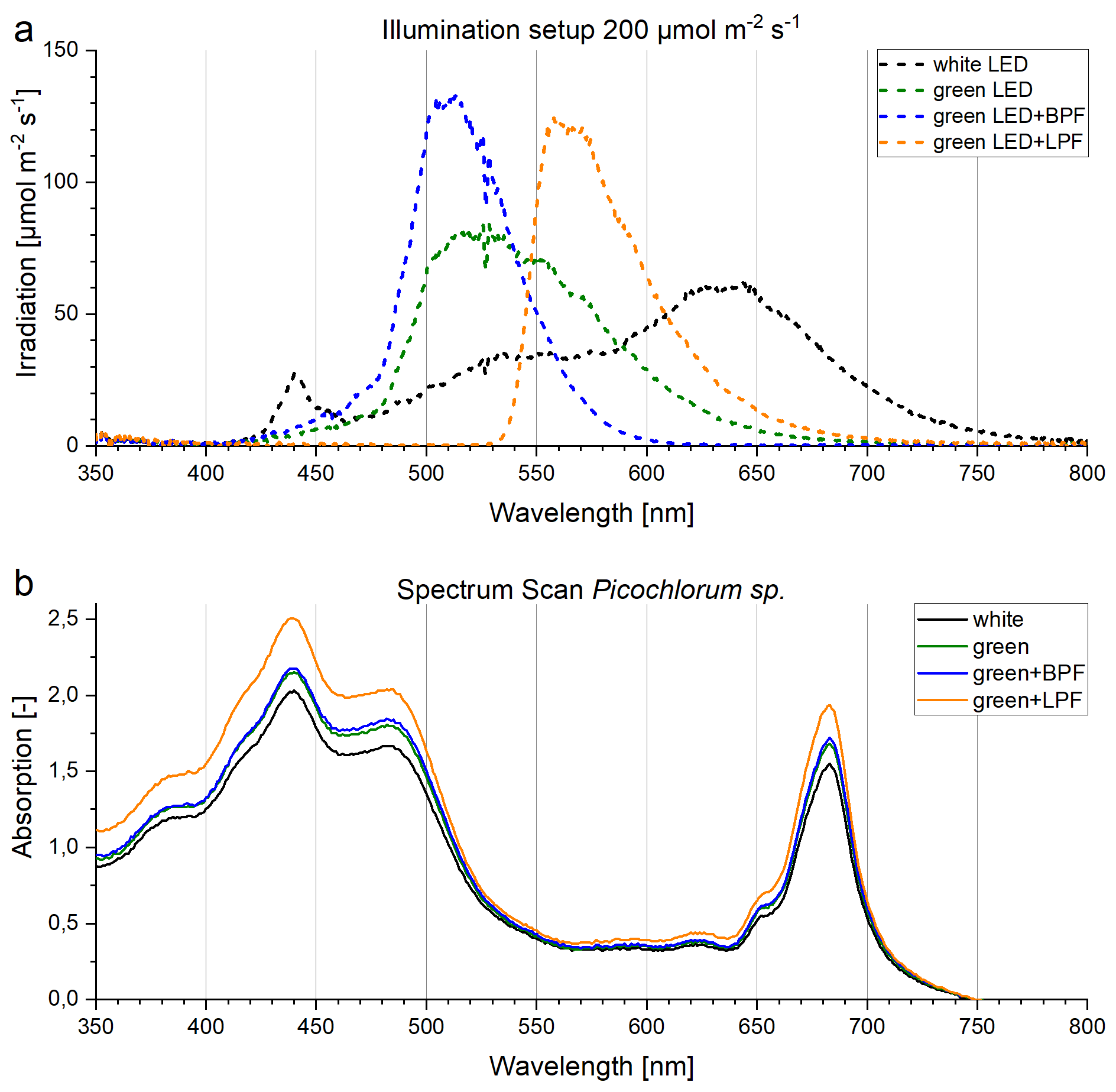

Supplement: Supplementary file 2 [file Image6.TIF]

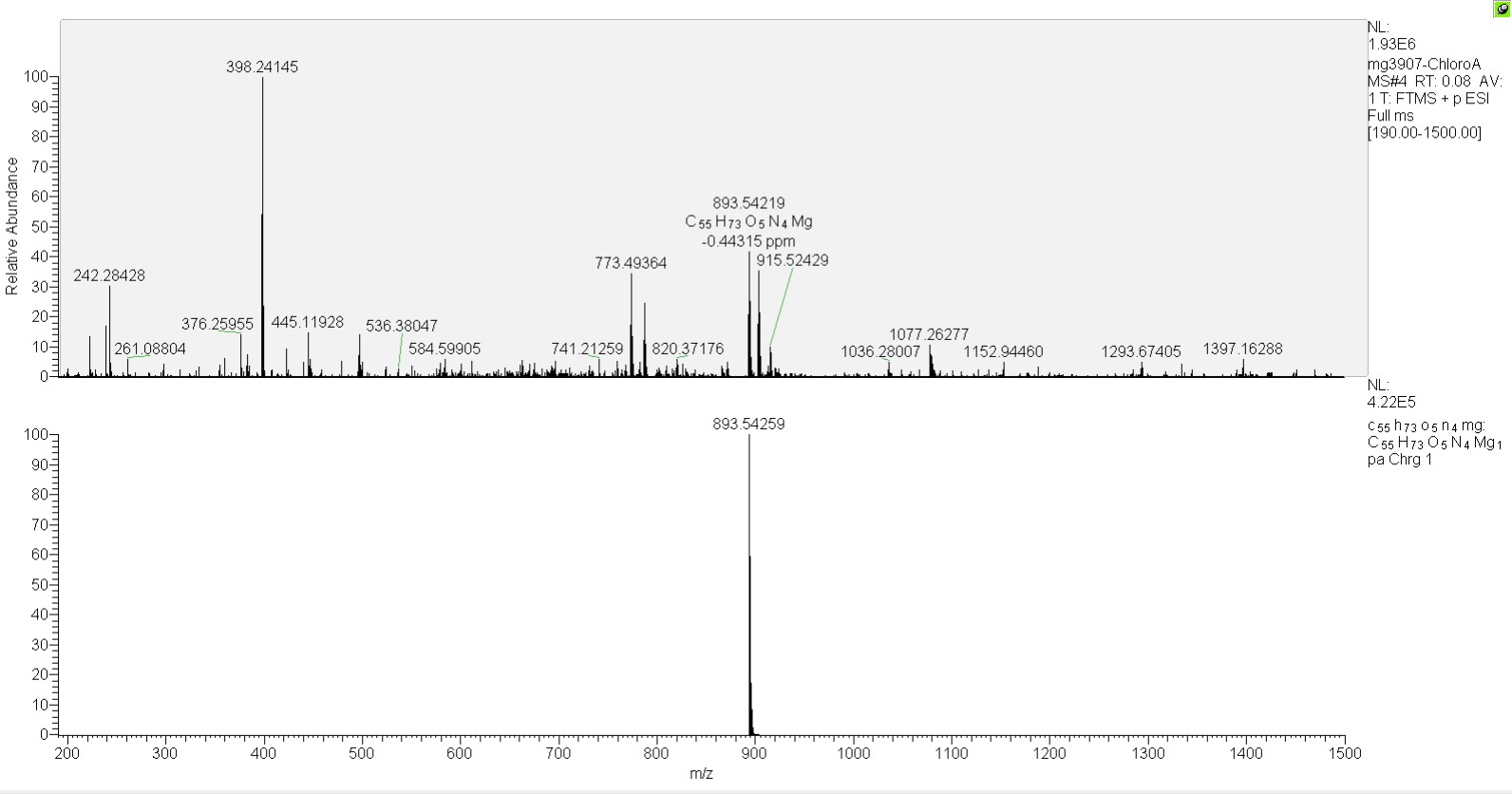

Supplement: Supplementary file 3 [file Image9.JPEG]

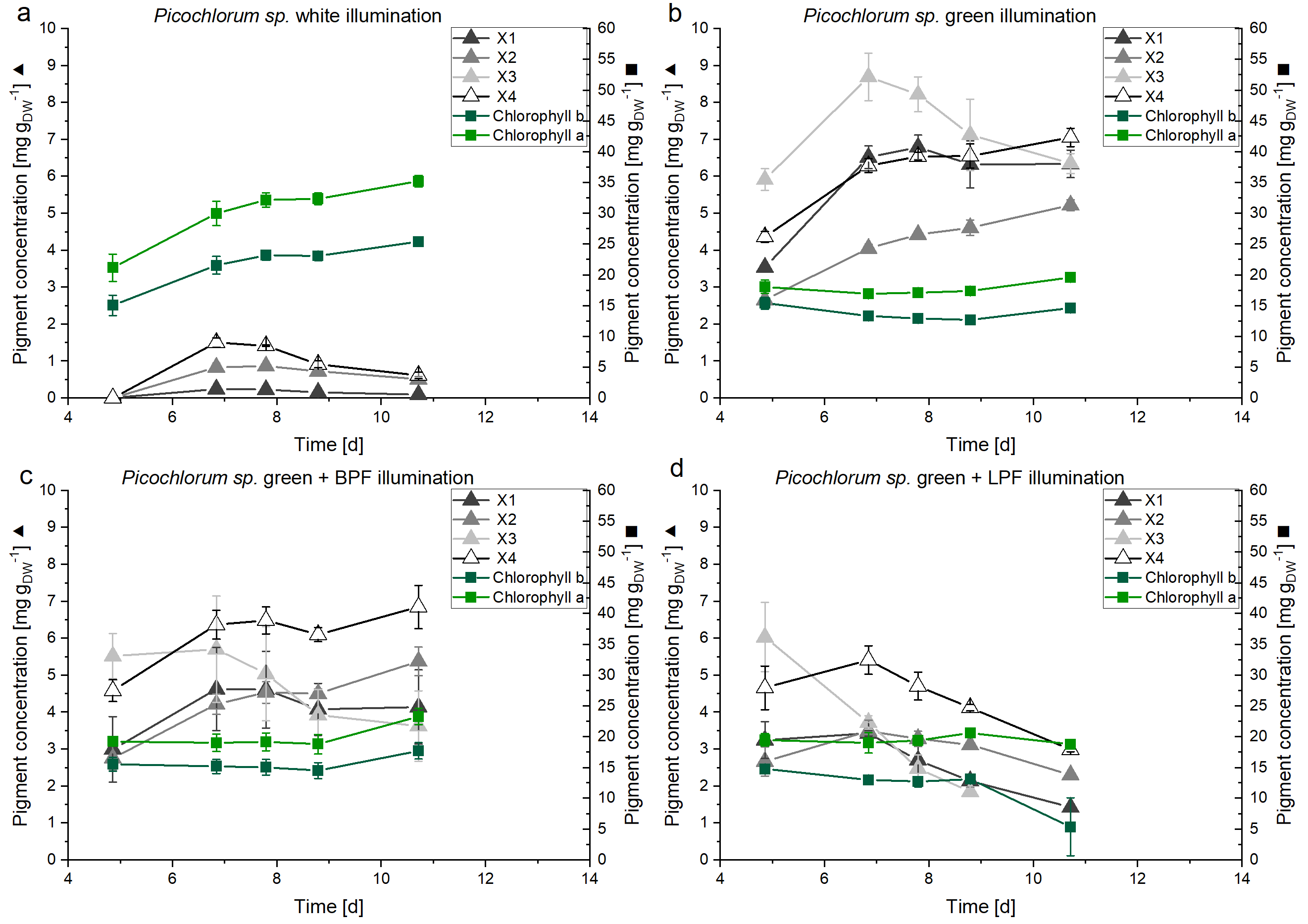

Supplement: Supplementary file 4 [file Image3.TIF]

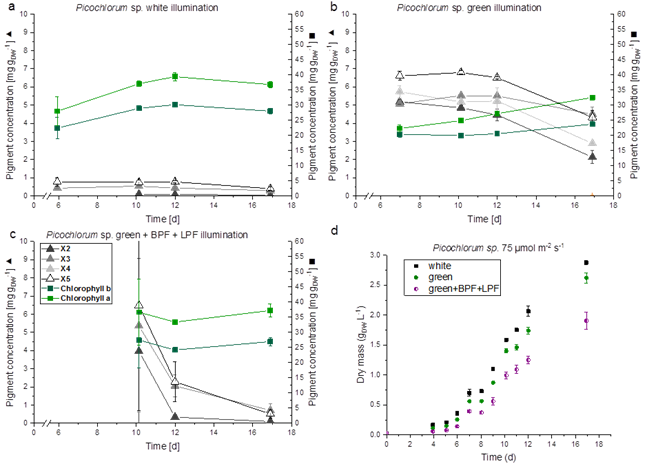

Supplement: Supplementary file 5 [file Image4.TIF]

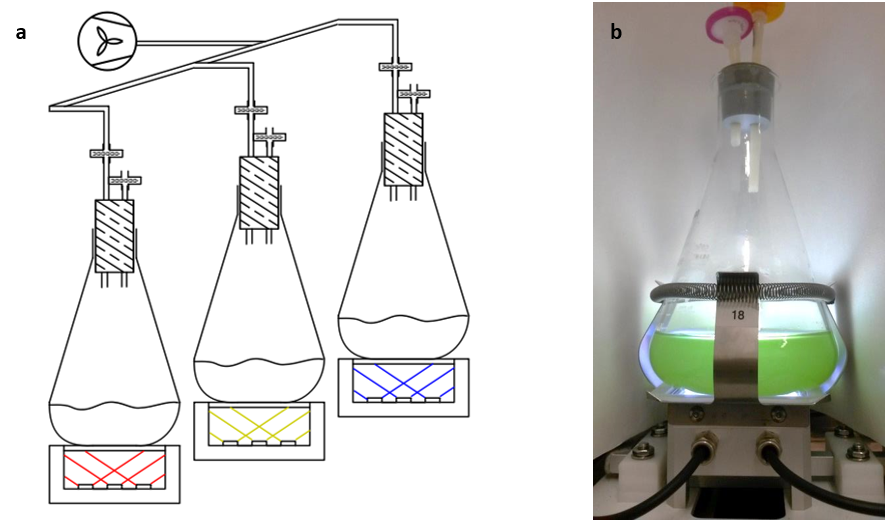

Supplement: Supplementary file 6 [file Image2.TIF]

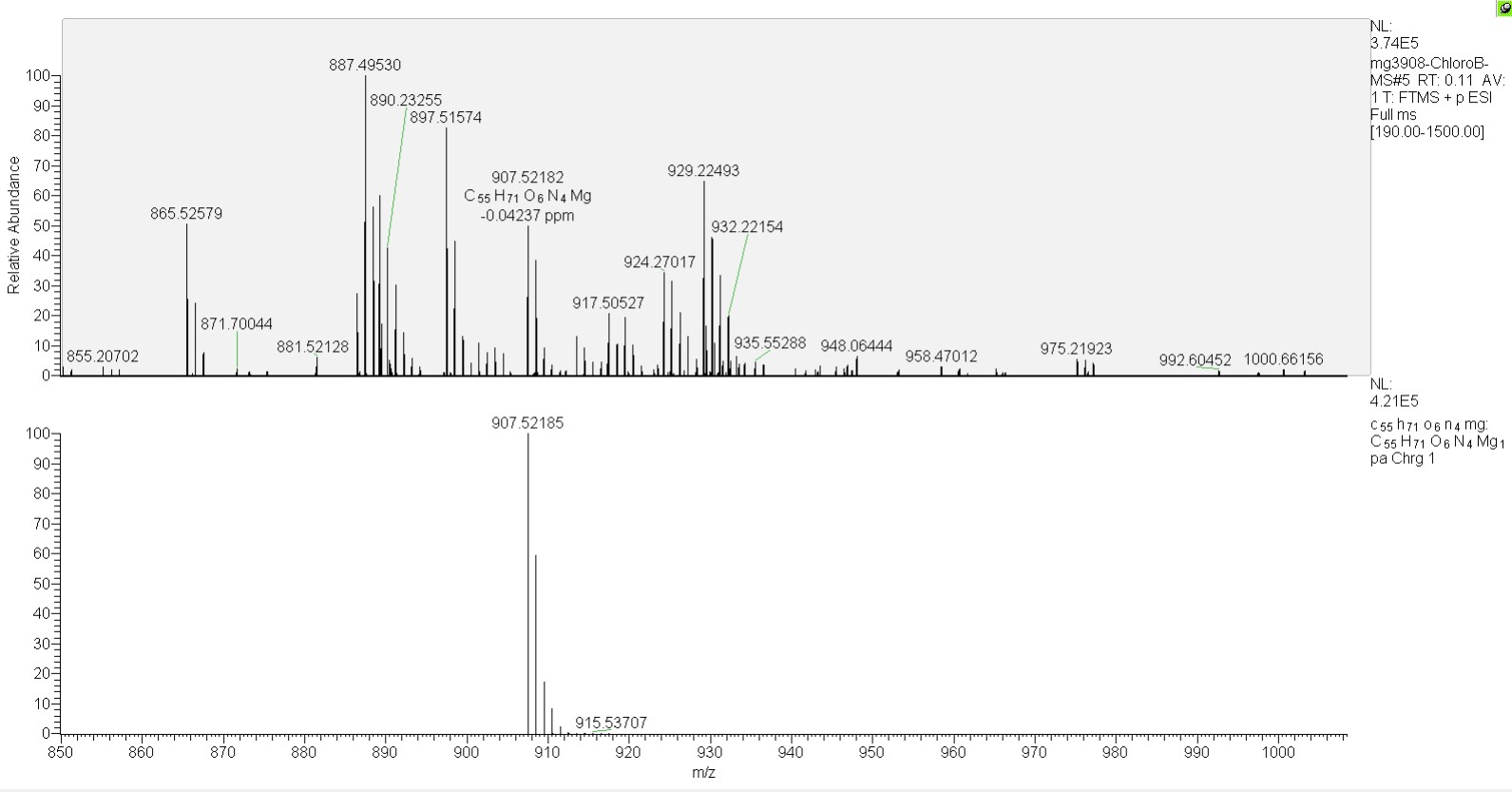

Supplement: Supplementary file 7 [file Image10.JPEG]

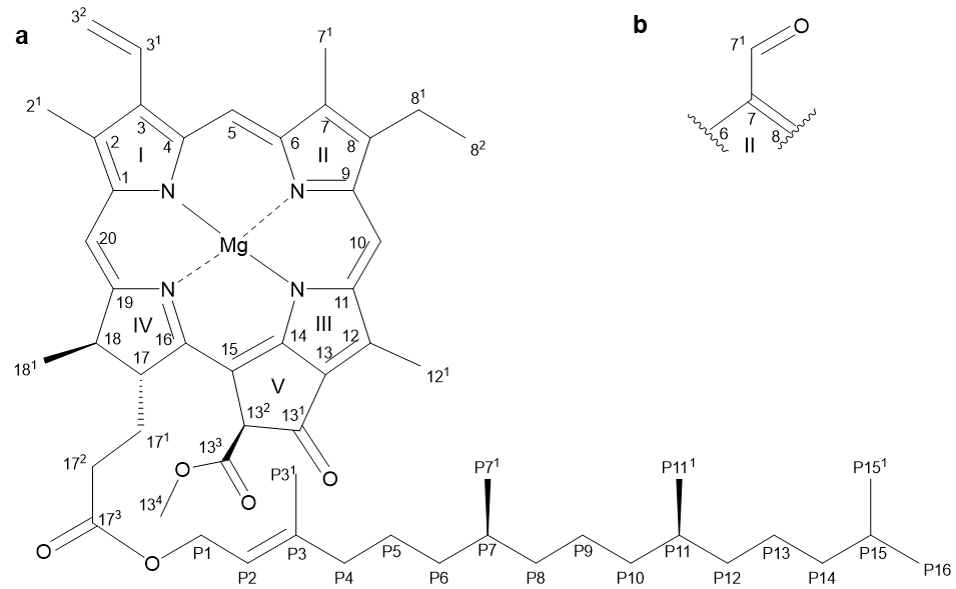

Supplement: Supplementary file 8 [file Image1.TIF]

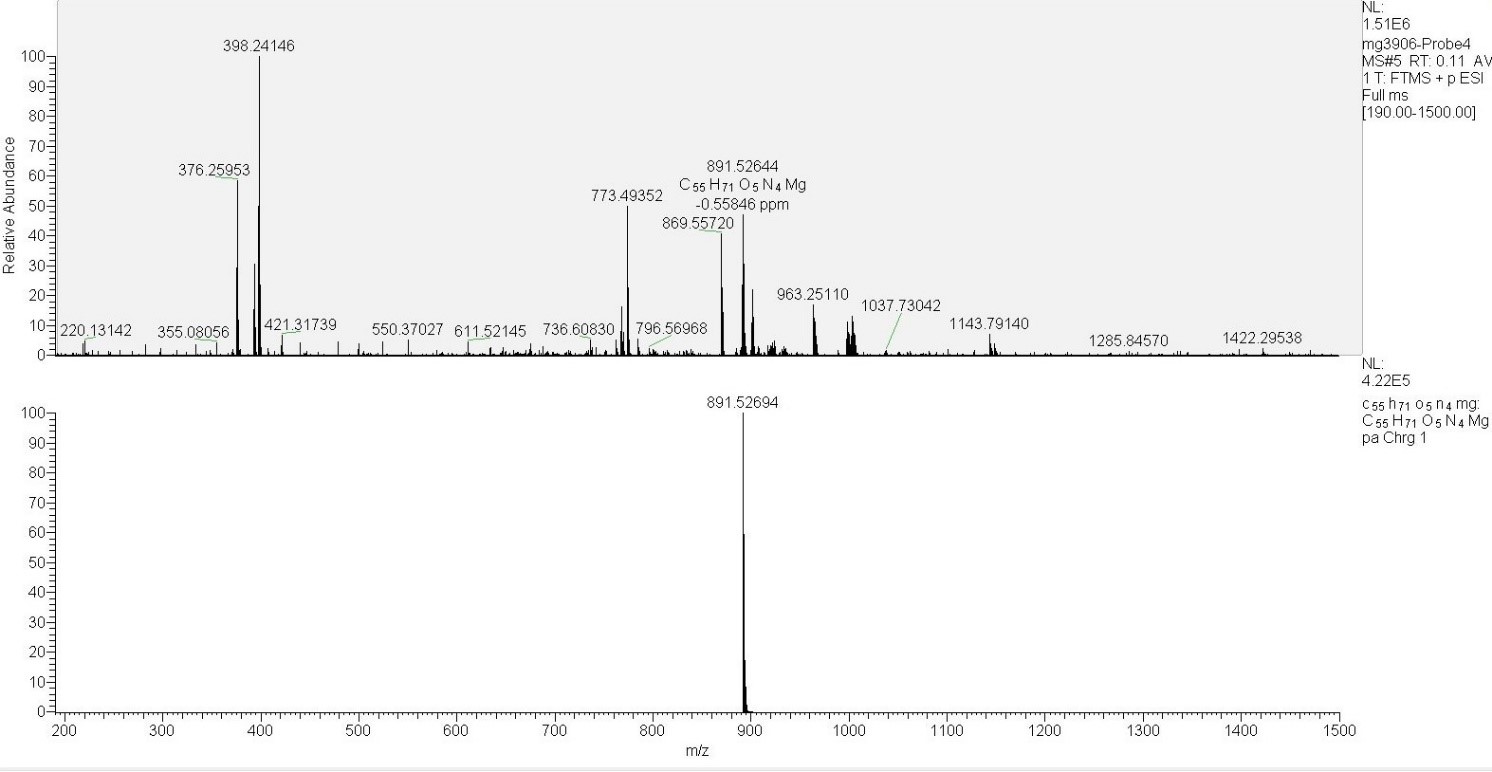

Supplement: Supplementary file 9 [file Image14.JPEG]

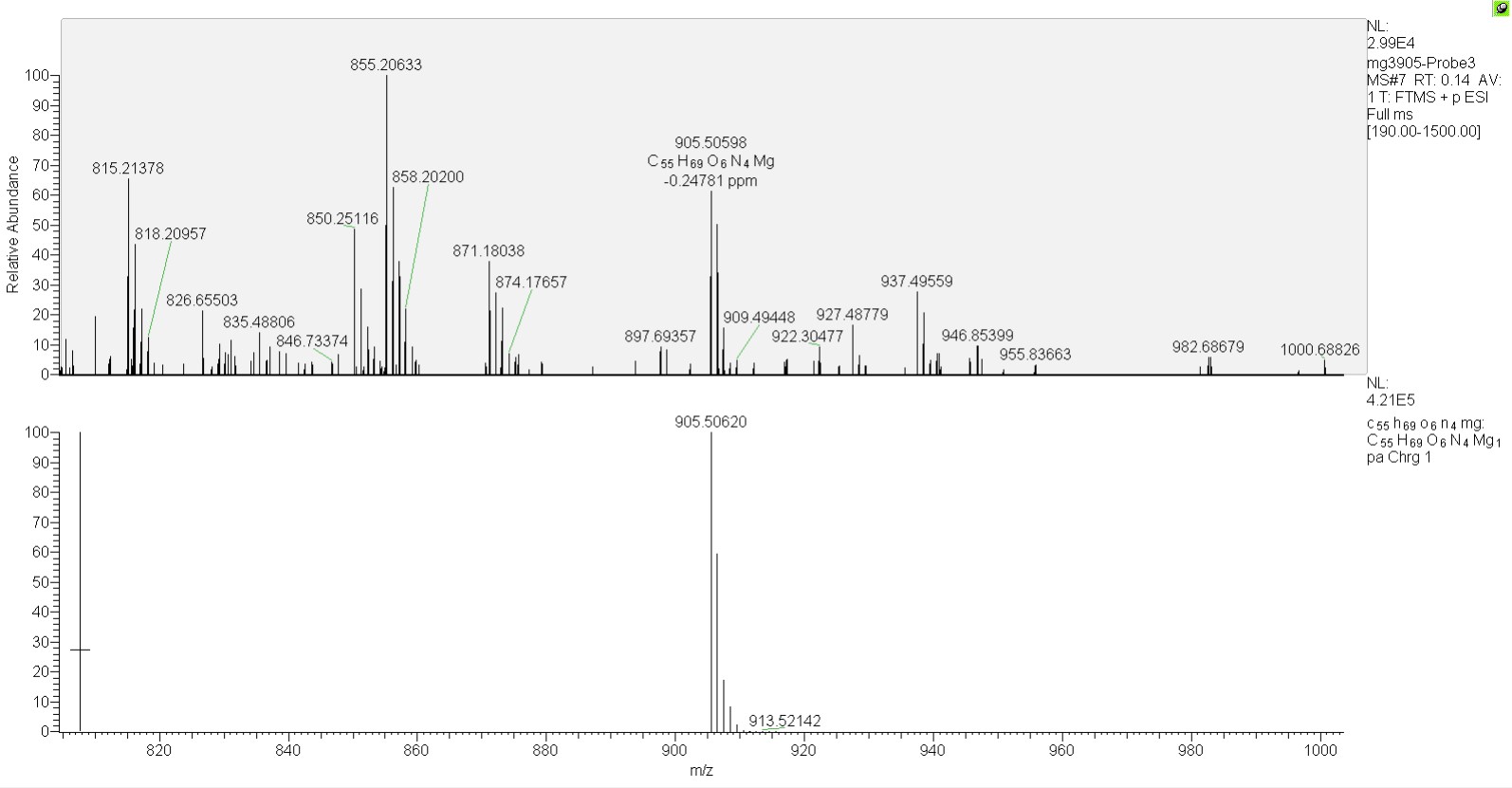

Supplement: Supplementary file 10 [file Image12.JPEG]

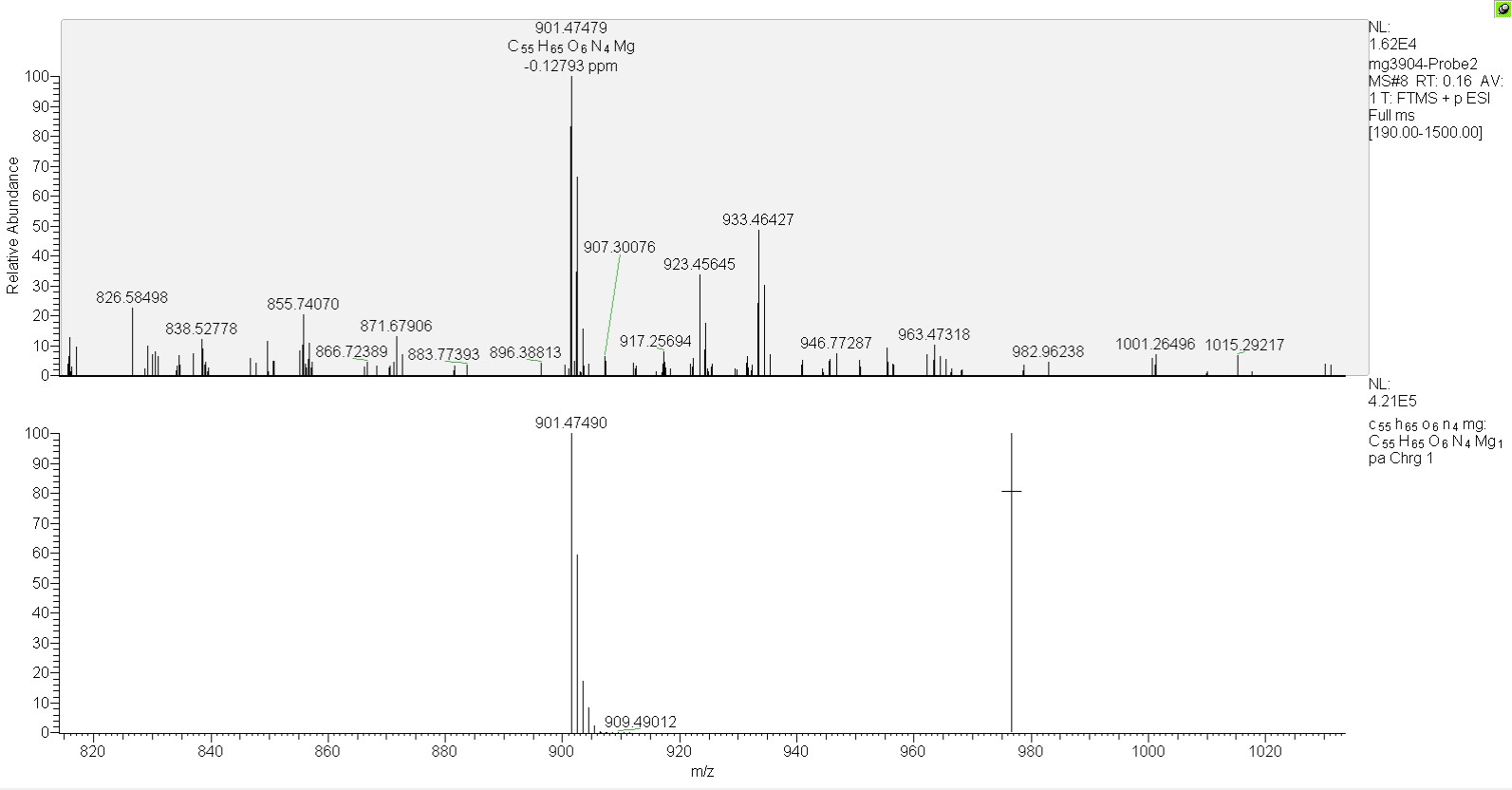

Supplement: Supplementary file 11 [file Image11.JPEG]

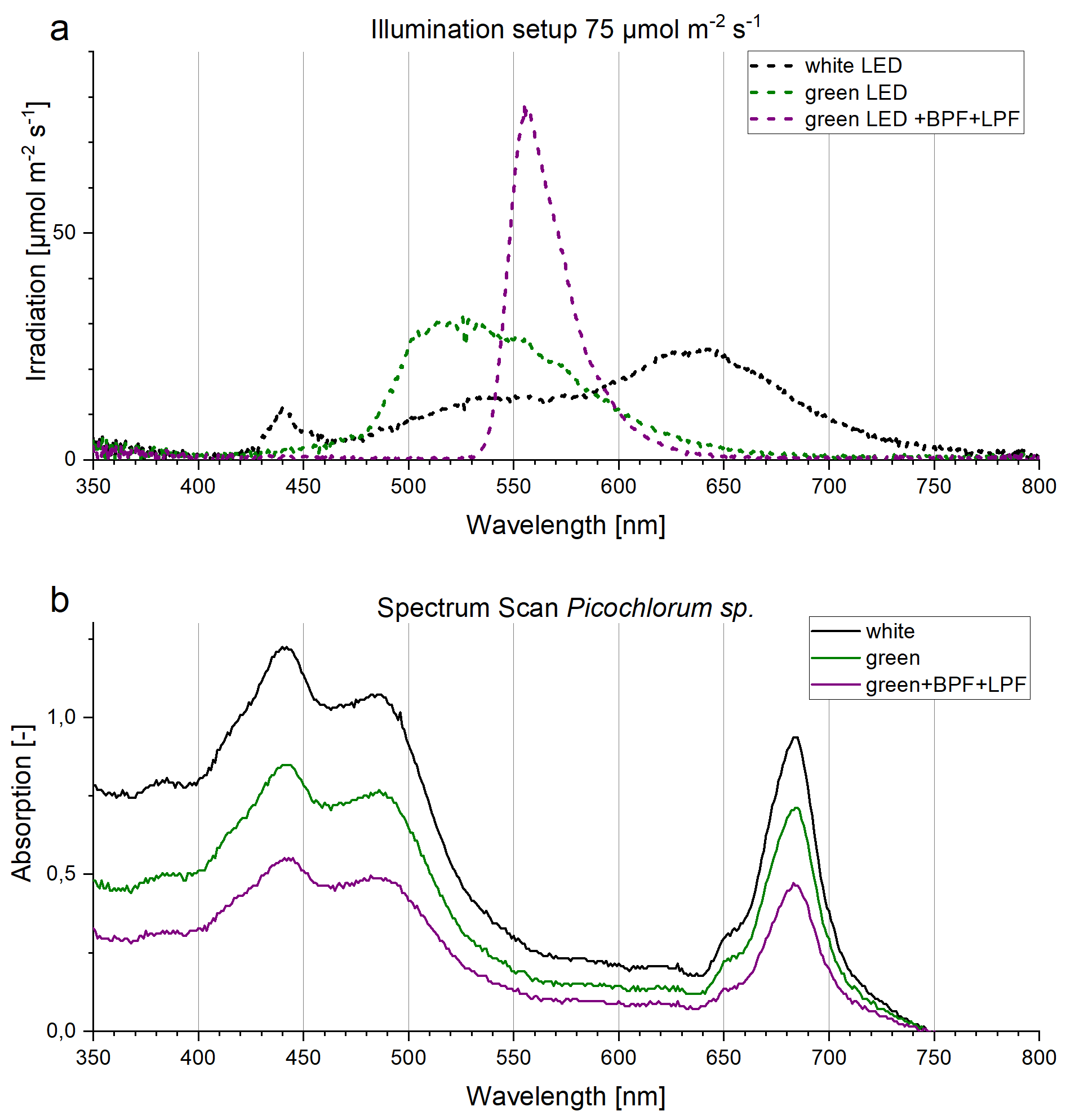

Supplement: Supplementary file 12 [file Image7.TIF]

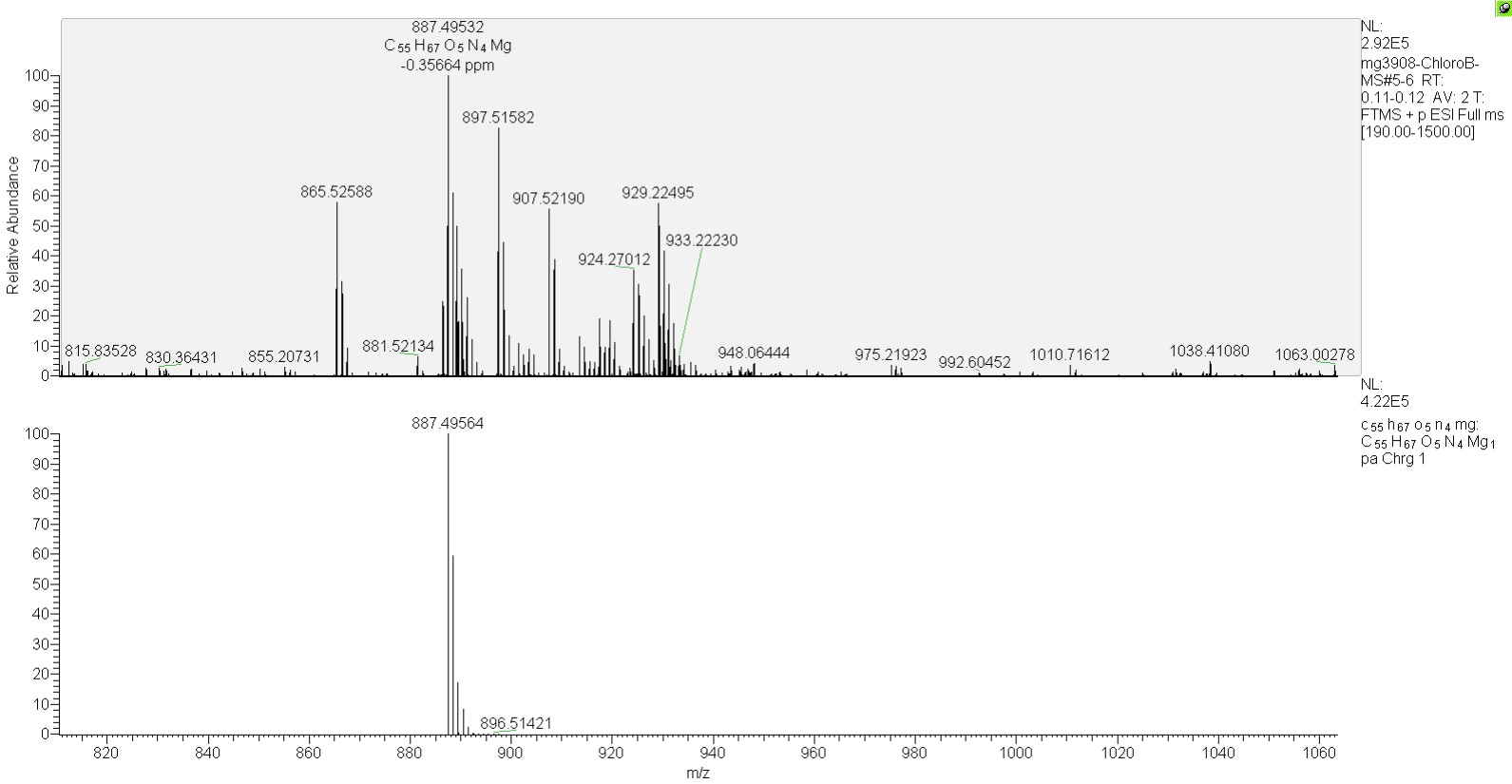

Supplement: Supplementary file 14 [file Image13.JPEG]

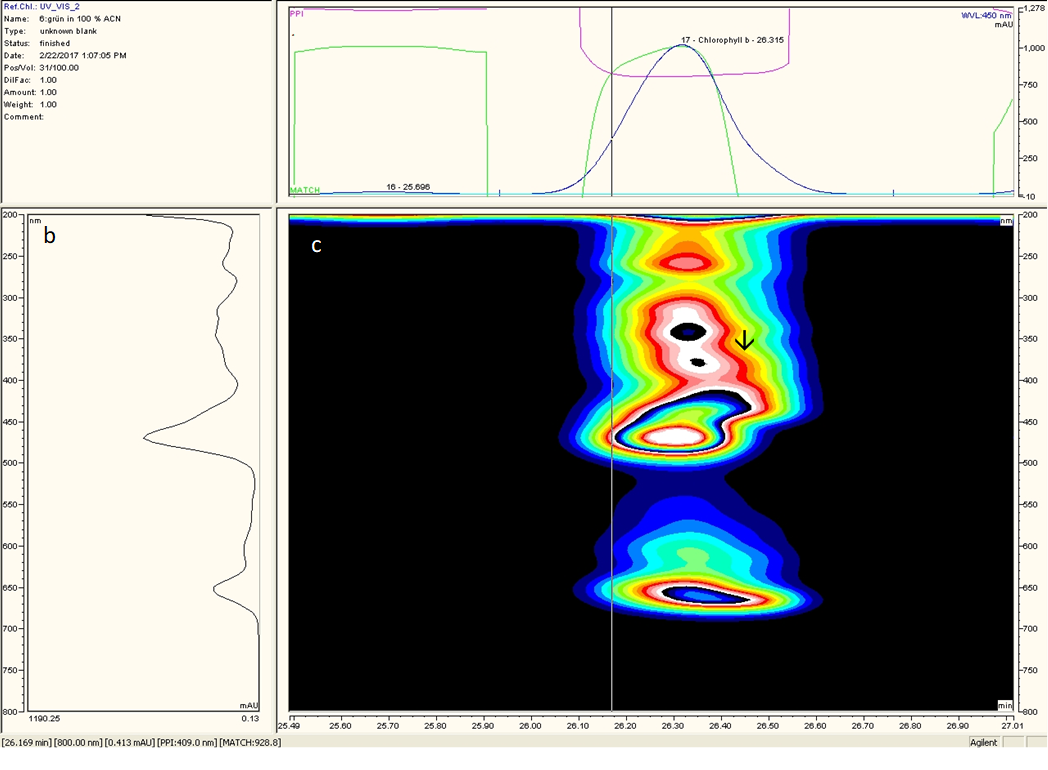

Supplement: Supplementary file 15 [file Image8.TIF]

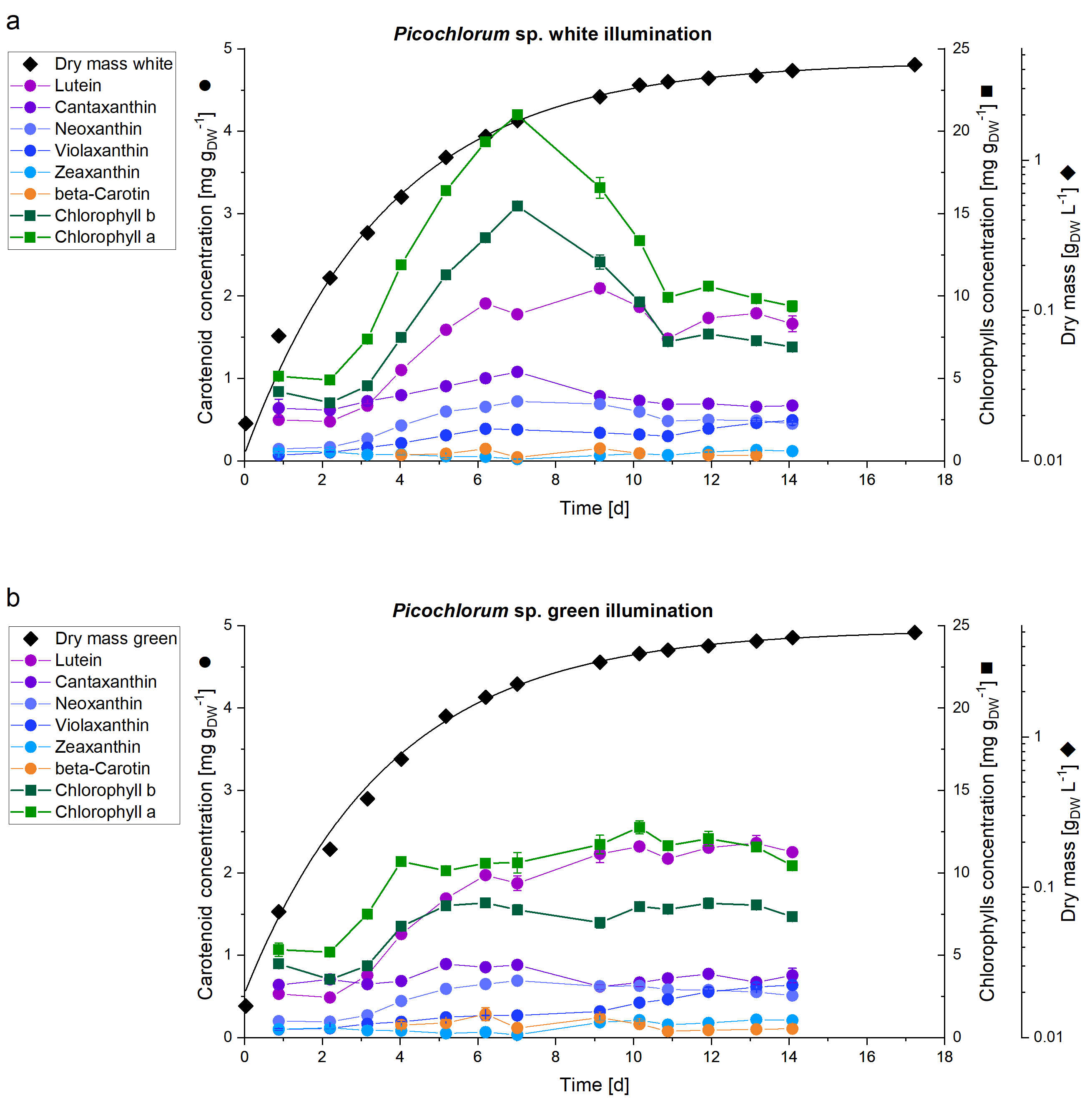

Supplement: Supplementary file 16 [file Image5.TIF]
