# Supplementary material for: Efficient Green Light Acclimation of the Green Algae Picochlorum sp. Triggering Geranylgeranylated Chlorophylls
Source: Front Bioeng Biotechnol. 2022 Apr 28;10:885977. doi: 10.3389/fbioe.2022.885977 (PMC9095919; doi:10.3389/fbioe.2022.885977)
Supplement: Supplementary file 13 [file DataSheet1.PDF]

Supplementary Material

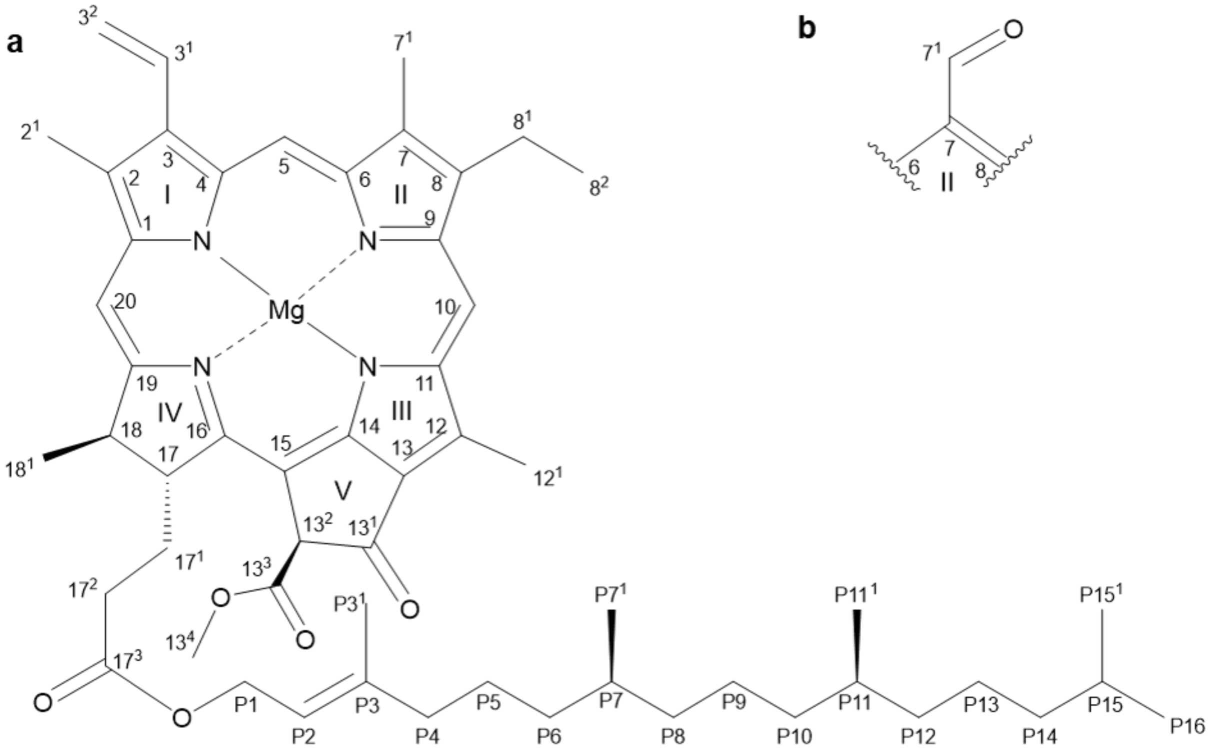

Supplementary Figure 1: Chlorophyll *a* with IUPAC numbering of carbon atoms. b) Section of chlorophyll *b* showing the oxidized residue at carbon atom 7^1.

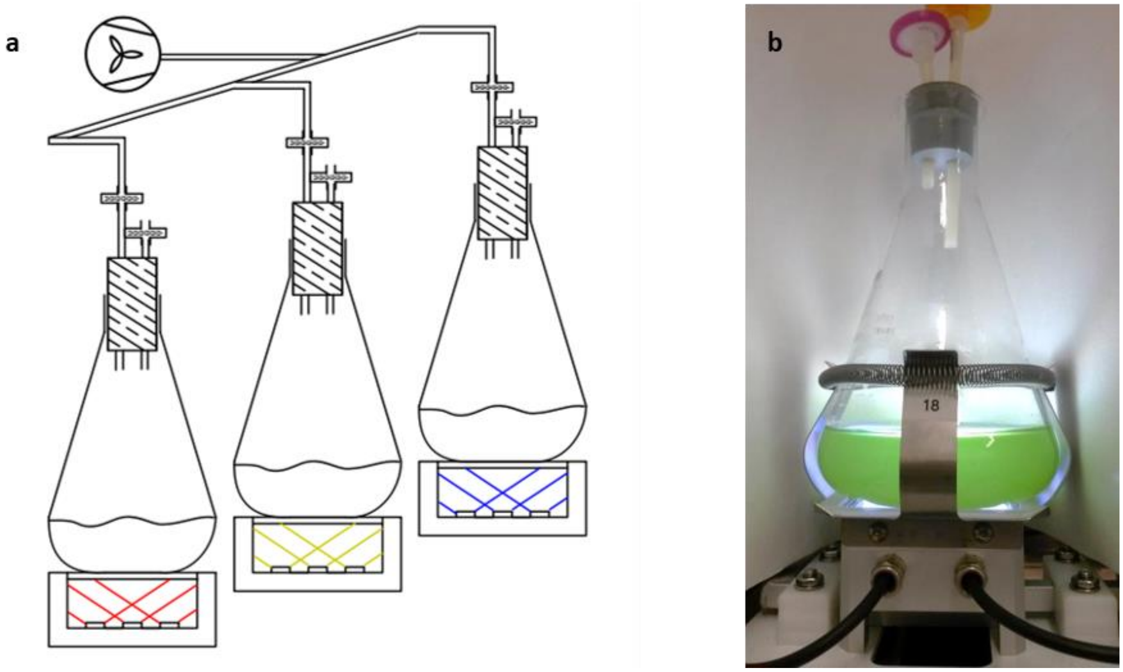

Supplementary Figure 2: a) Graphical scheme of 3 individually illuminated LED-Sockets. Erlenmeyer flasks equipped with custom made, airtight stoppers equipped with sterile filters, attached to an aeration system (massflow control DASGIP® MX4/4), b) figure of one attached and illuminated flask.

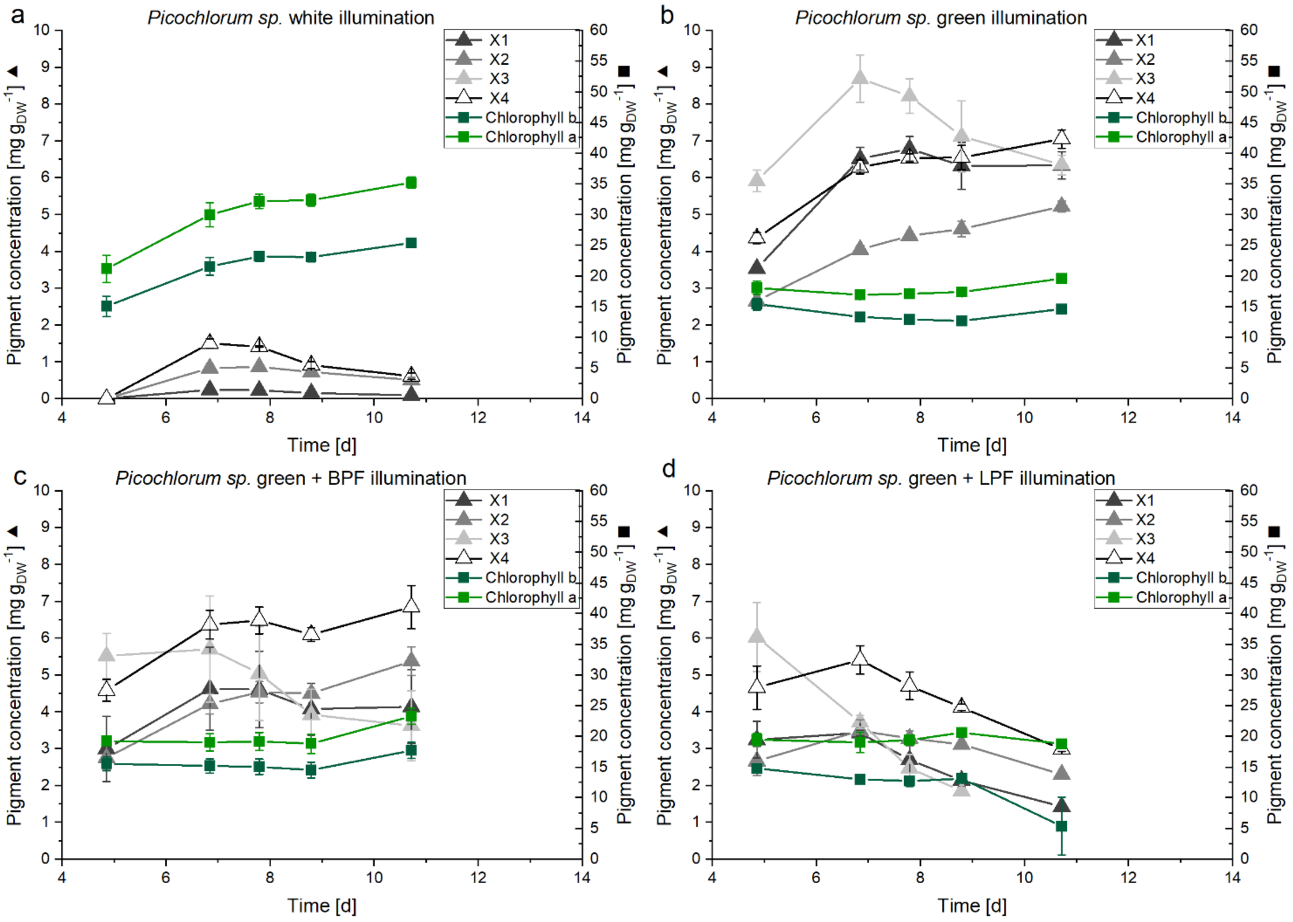

Supplementary Figure 3: High pressure liquid chromatography (HPLC) analysis of *Picochlorum sp.* pigments grown at 200 µmol m^{-2} s^{-1} irradiation with white, green, green + BPF (band-pass filter B 13) and green + LPF (long-pass filter O 540) colors. Right side axis for chlorophyll (Chl) *a* and *b* (squares). X1-X4: unknown pigments generated under green light illumination. Experiments were carried out in biological triplicates (n=3).

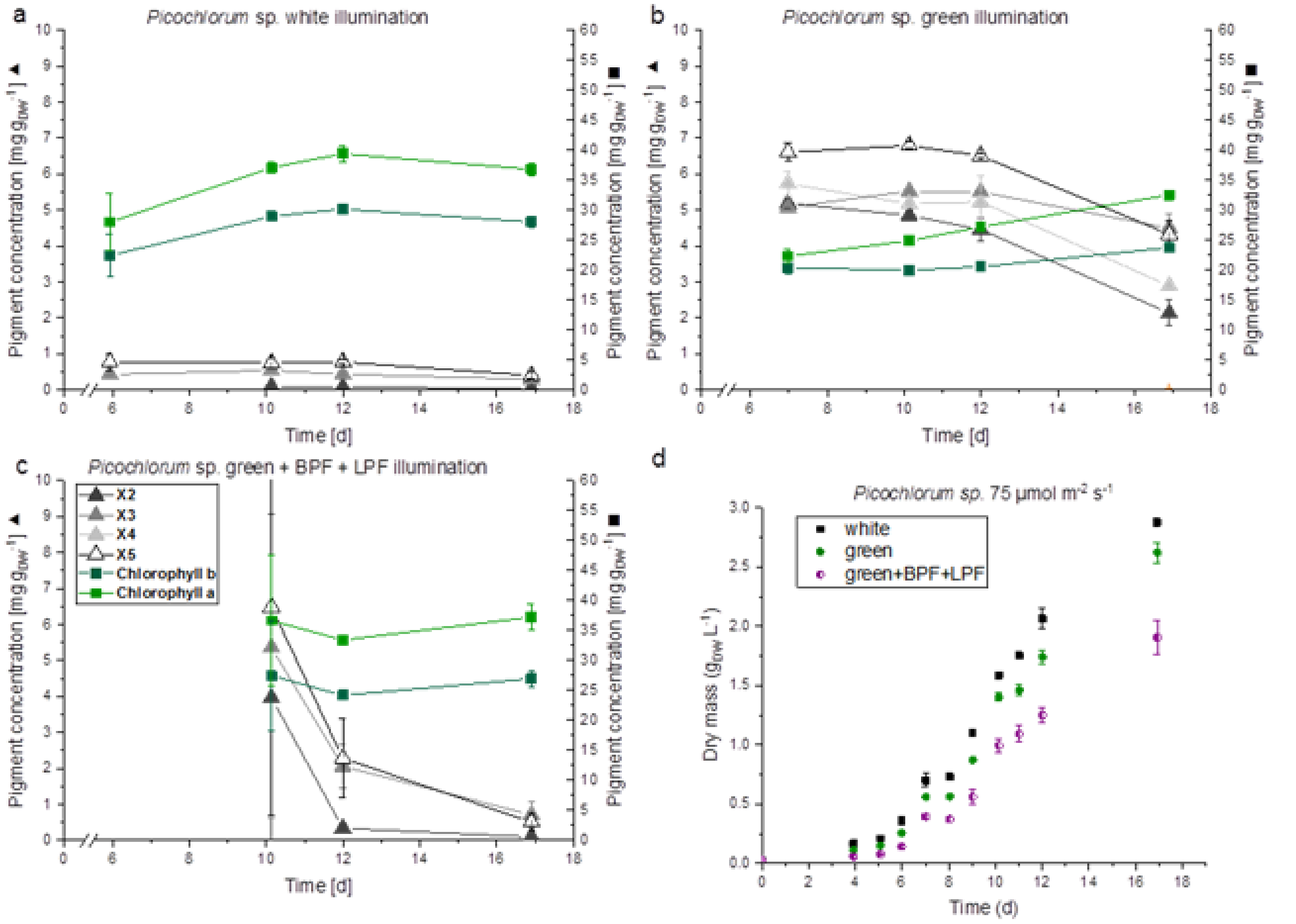

Supplementary Figure 4: HPLC analysis of *Picochlorum sp.* pigments grown at 75 µmol m^{-2} s^{-1} irradiation with white (a), green (b) and double-bandwidth diminishment green+BPF (band-pass filter B 13) +LPF (long-pass filter O 540), (c) illumination. The right side axes show concentrations for chlorophyll *a* and *b*, while the left side axes show the concentrations of the unknown pigments. X1-X4: unknown pigment generated during green light illumination. Experiments were carried out in triplicates (n=3). The biomass growth is displayed in (d).

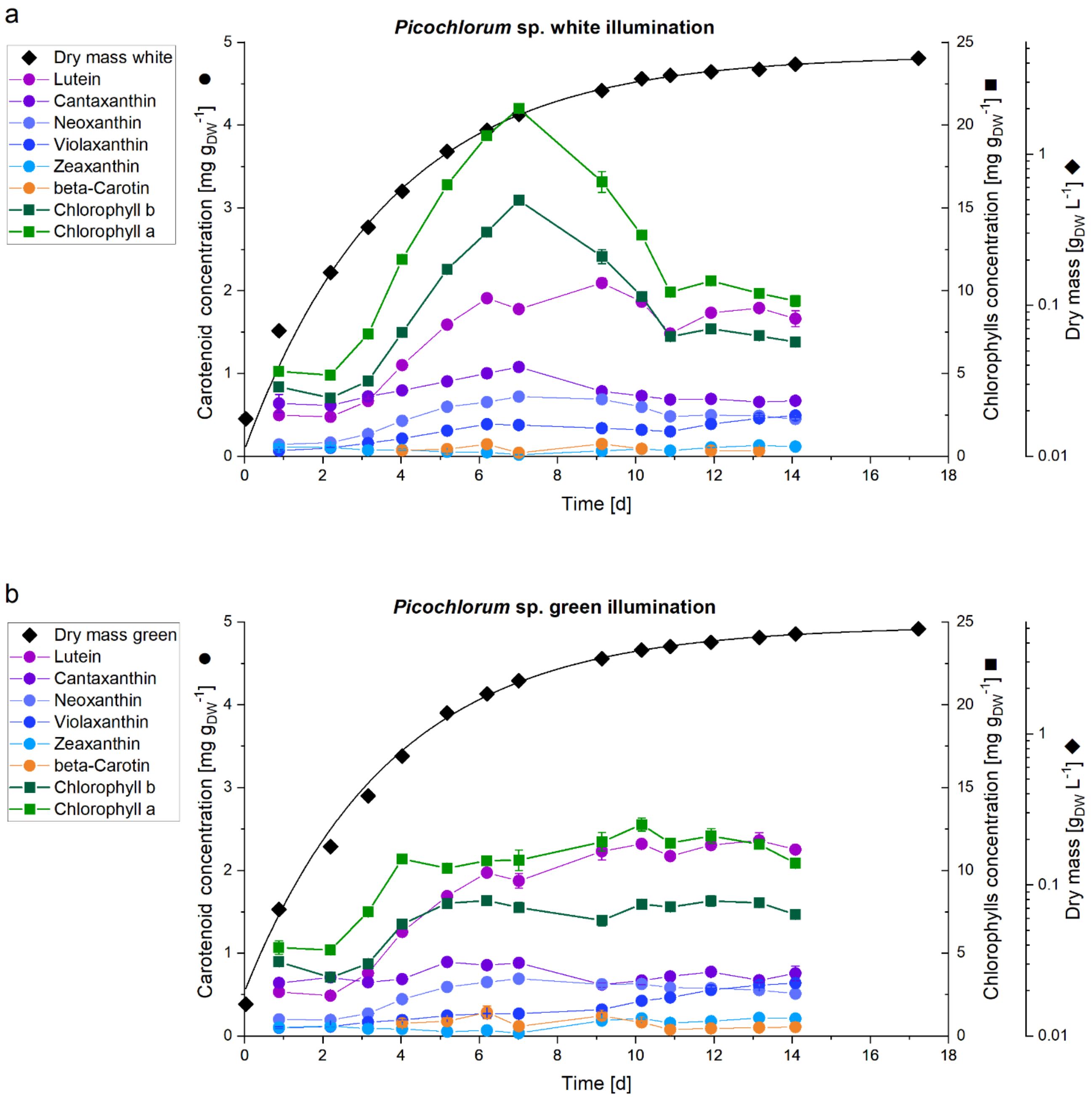

Supplementary Figure 5: HPLC analysis of pigments of a) white and b) green illuminated *Picochlorum sp.* cultivation. Dry mass values (diamond) on the far right logarithmic axis, known carotenoids (circles) development on left side axis, chlorophyll *a* and *b* (square) on the right side pigment axis.

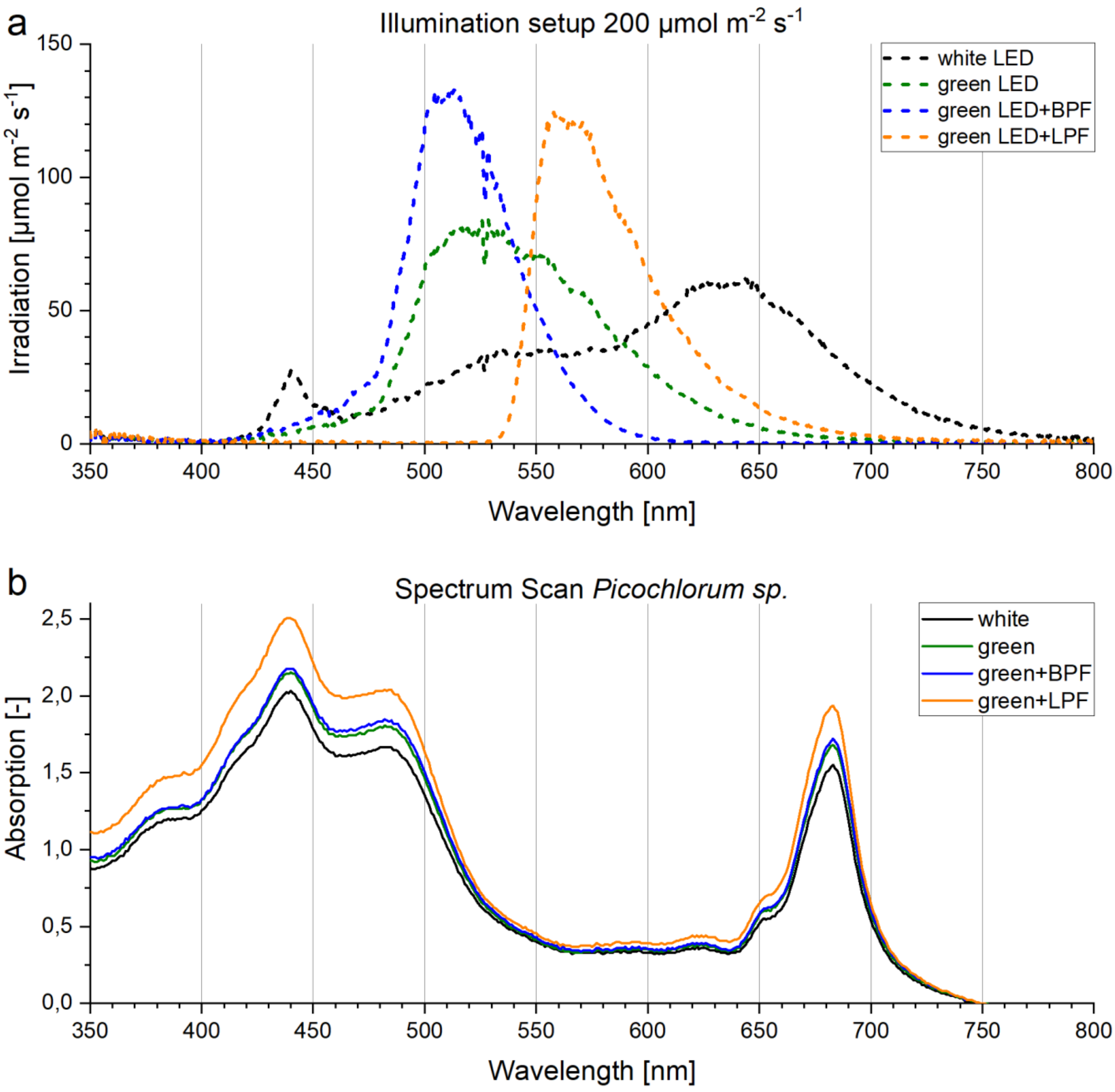

Supplementary Figure 6: a) Illumination setup at 200 µmol m^{-2} s^{-1} with glass filter plates installed on the LED-shaker platform. b) Spectrum scan 350 – 800 nm of vital *Picochlorum sp.* grown at white and green light illumination as well as green+LPF and green+BPF modified light illumination with 200 µmol m^{-2} s^{-1} irradiation, scan at day 8, absorption normalized to 750 nm value. Measurement performed via plate reader.

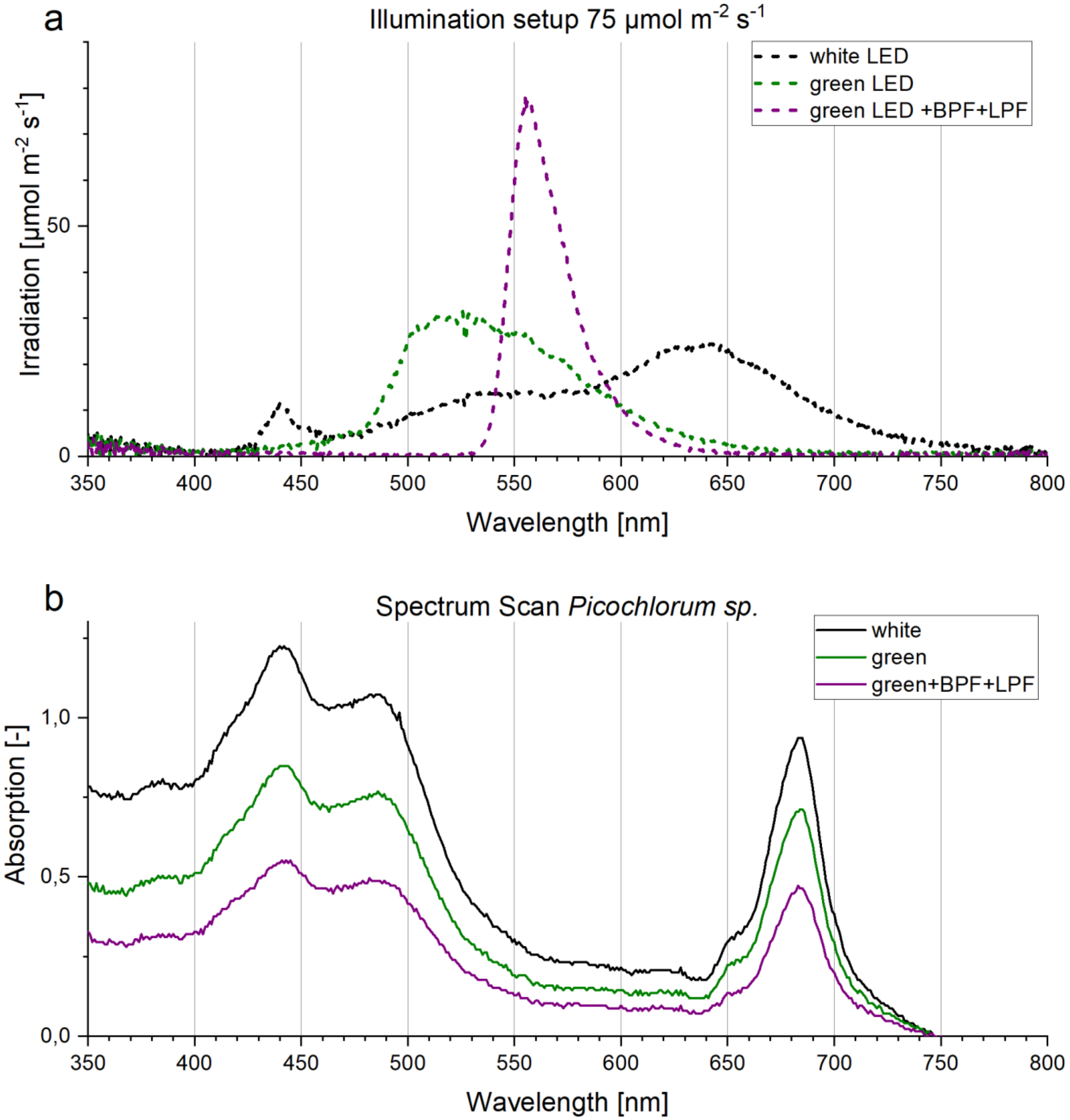

Supplementary Figure 7: a) Illumination setup at 75 µmol m^{-2} s^{-1} with glass filter plates installed on the LED-shaker platform. b) Spectrum scan 350 – 800 nm of *Picochlorum sp.*, scan at day 10, absorption normalized to 750 nm value. Measurement of live cells performed via plate reader.

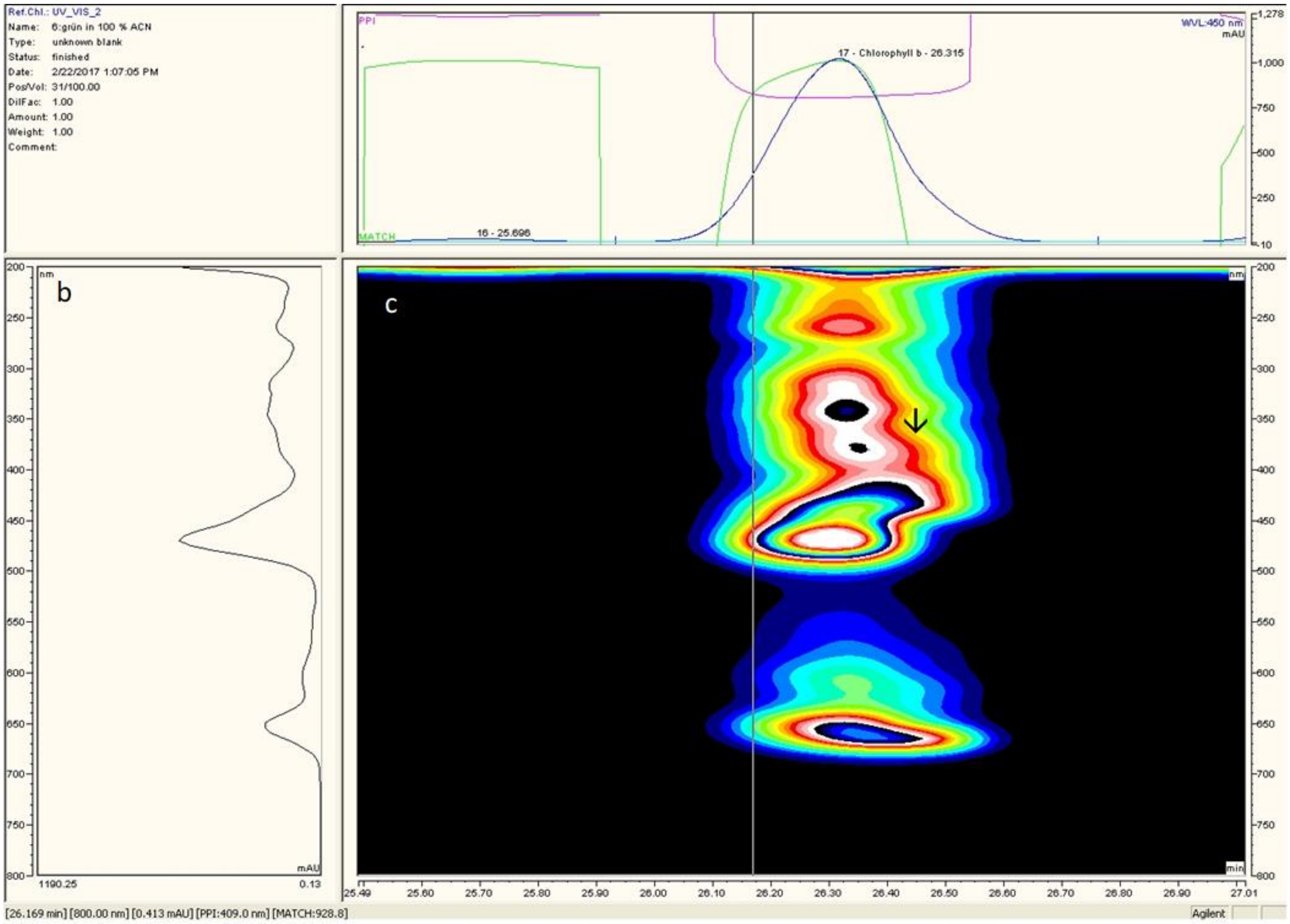

Supplementary Figure 8: UV-VIS Spectra of chlorophyll b extracted from *Picochlorum sp.* grown at green light. a) chromatogram b) spectrograph c) 3D display. Black arrow signaling occurrence of unknown pigment in the bulge of the 3d display of chlorophyll *b.*

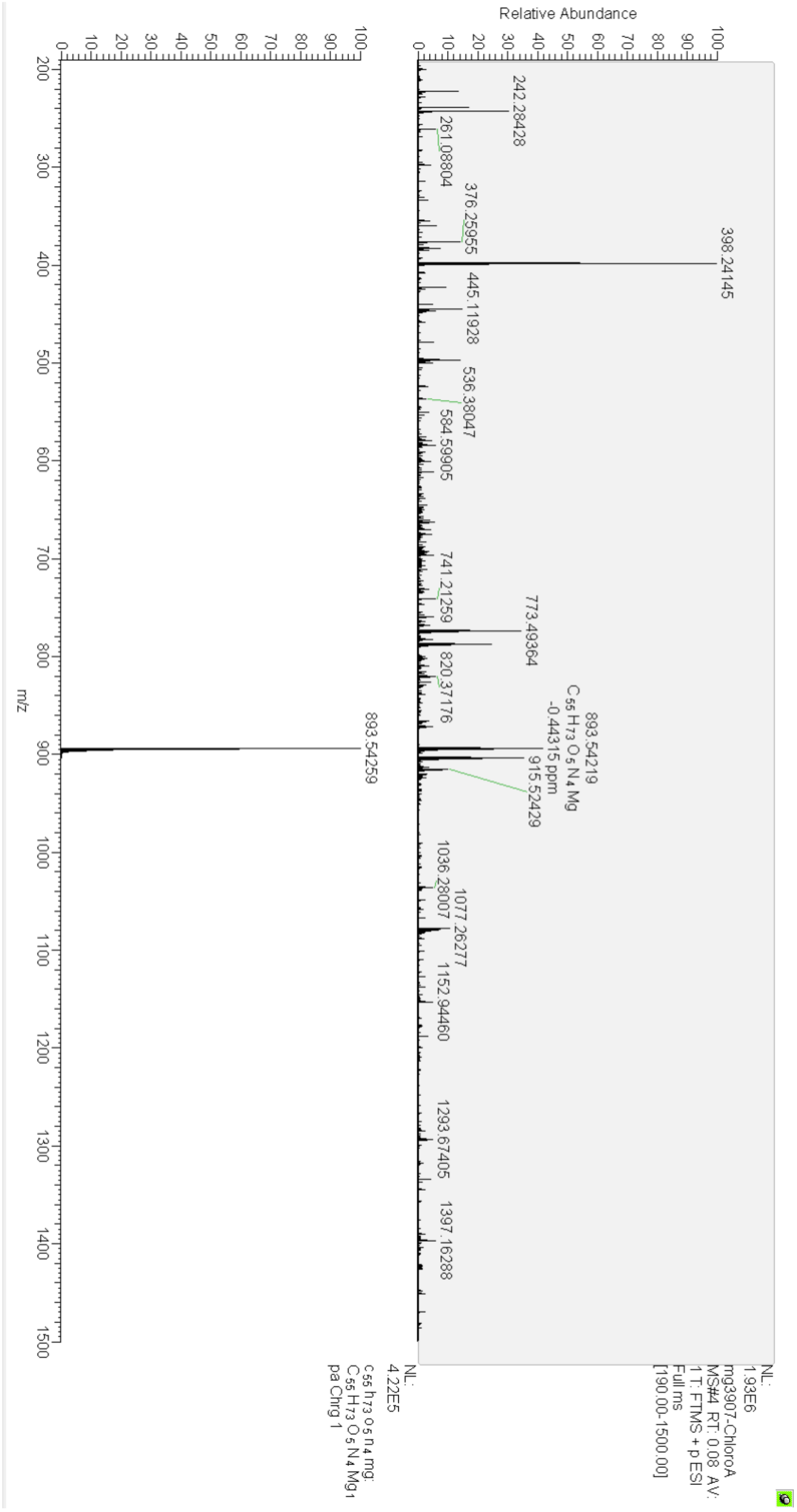

Supplementary Figure 9: High resolution MS full scan of Chl *a* in positive mode. a) MS-data relative abundance over m/z distribution. b) Simulated mass distribution of $C_{55}H_{73}O_5N_4Mg$

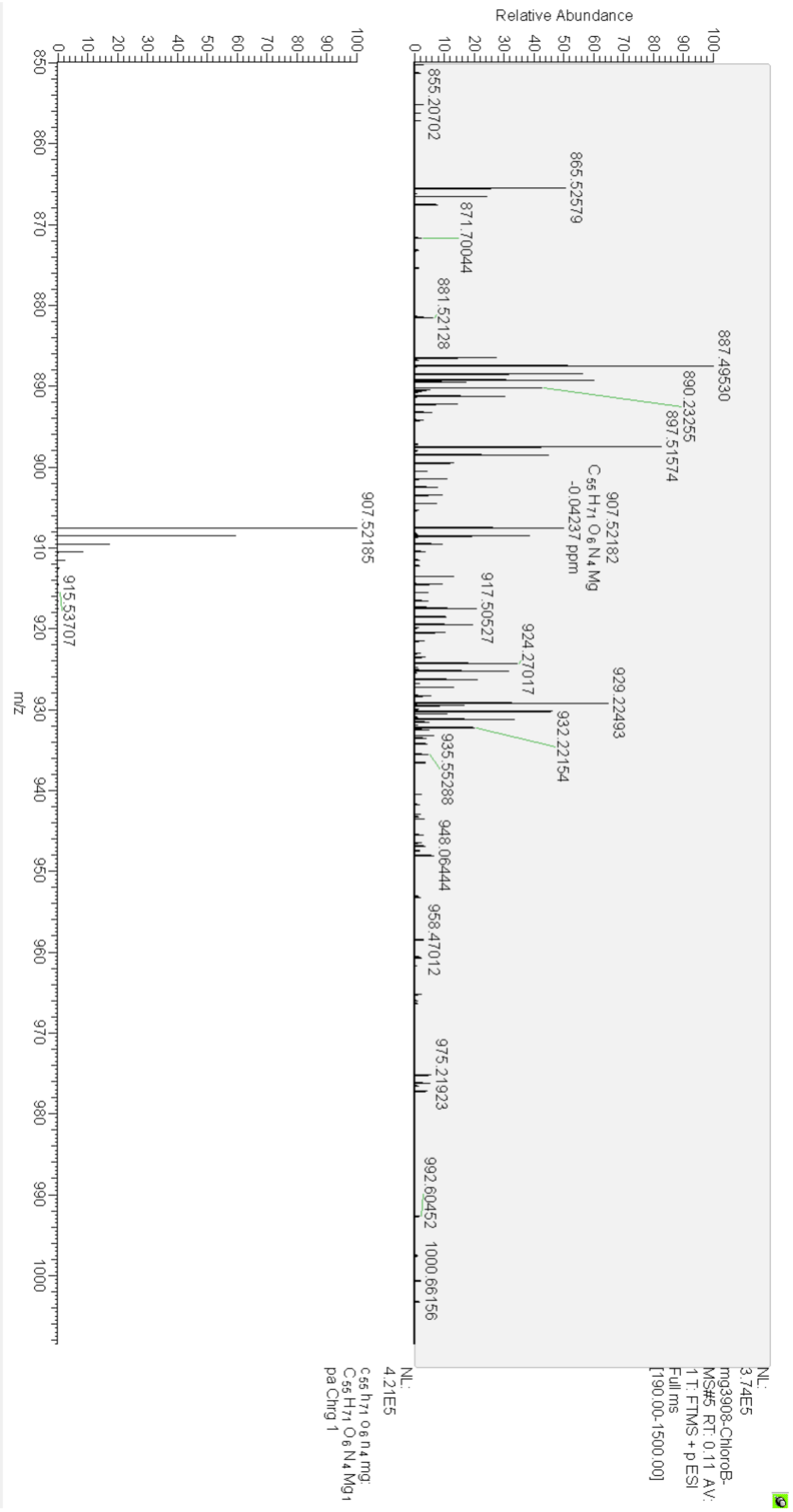

Supplementary Figure 10: High resolution MS full scan of Chl *b* (including X3) in positive mode. a) MS-data relative abundance over m/z distribution. b) Simulated mass distribution of $C_{55}H_{71}O_6N_4Mg$

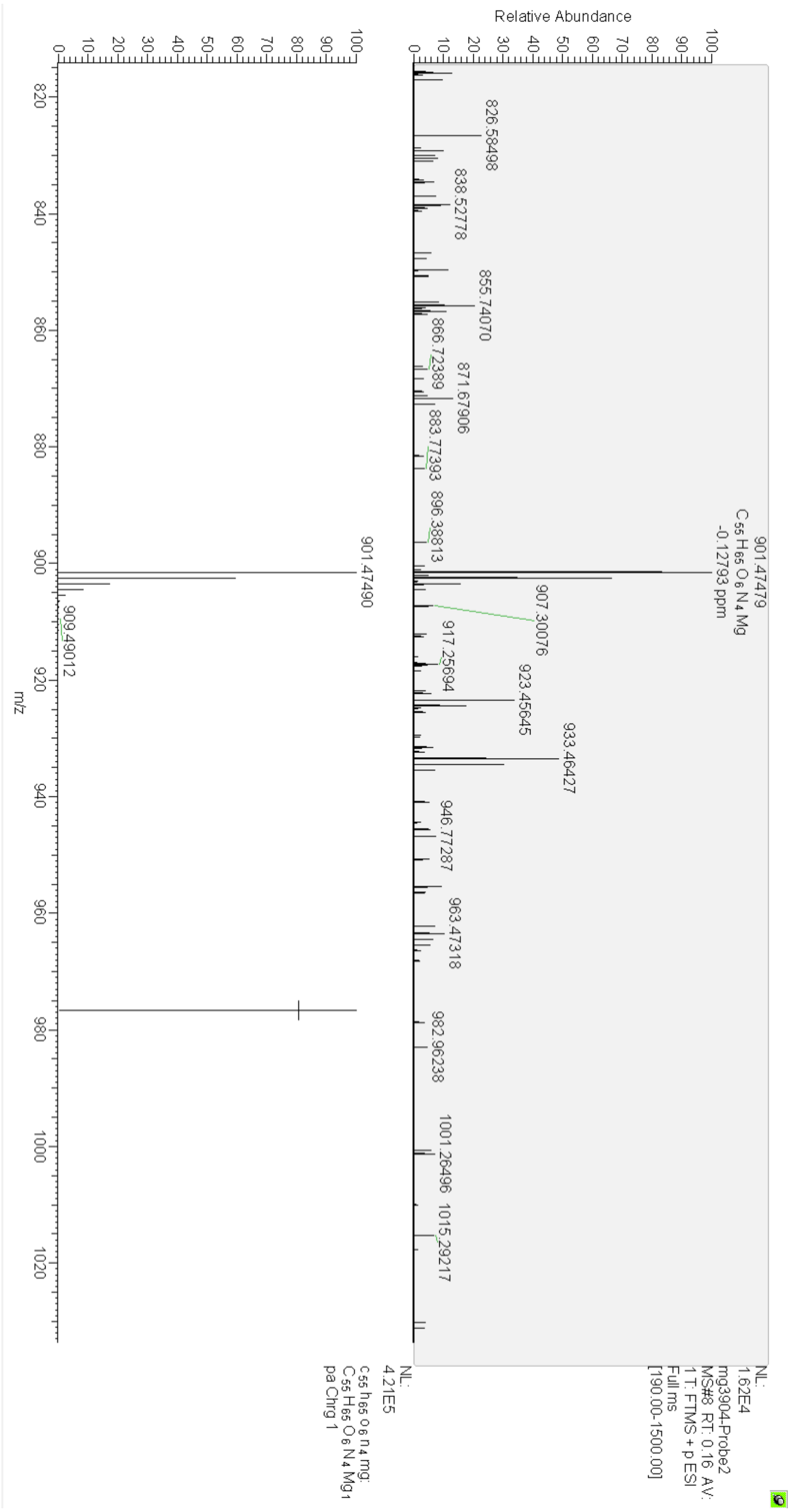

Supplementary Figure 11: High resolution MS full scan of unknown pigment X1 in positive mode. a) MS-data relative abundance over m/z distribution. b) Simulated mass distribution of $C_{55}H_{65}O_6N_4Mg$

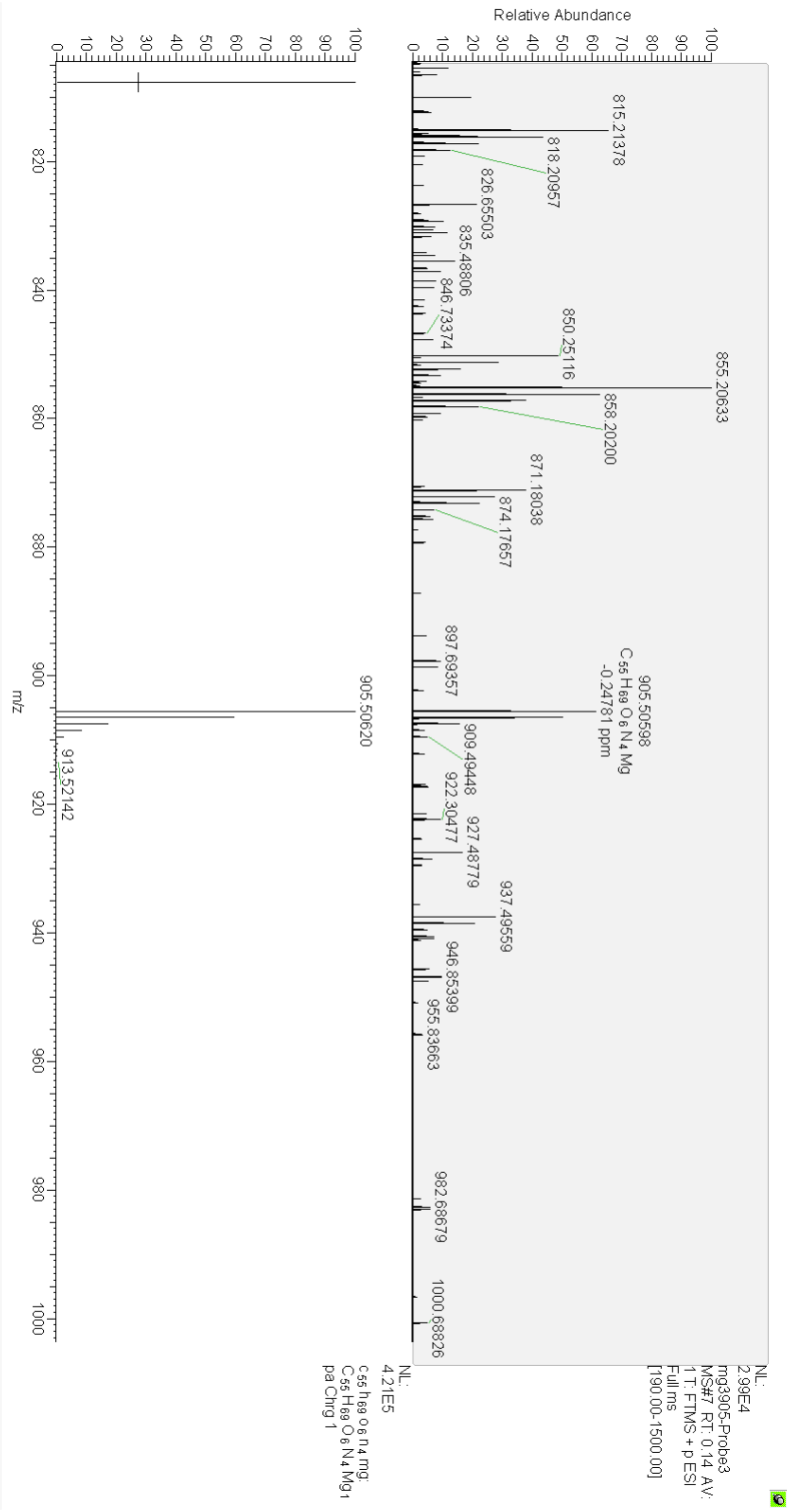

Supplementary Figure 12: High resolution MS full scan of unknown pigment X2 in positive mode. a) MS-data relative abundance over m/z distribution. b) Simulated mass distribution of $C_{55}H_{69}O_6N_4Mg$

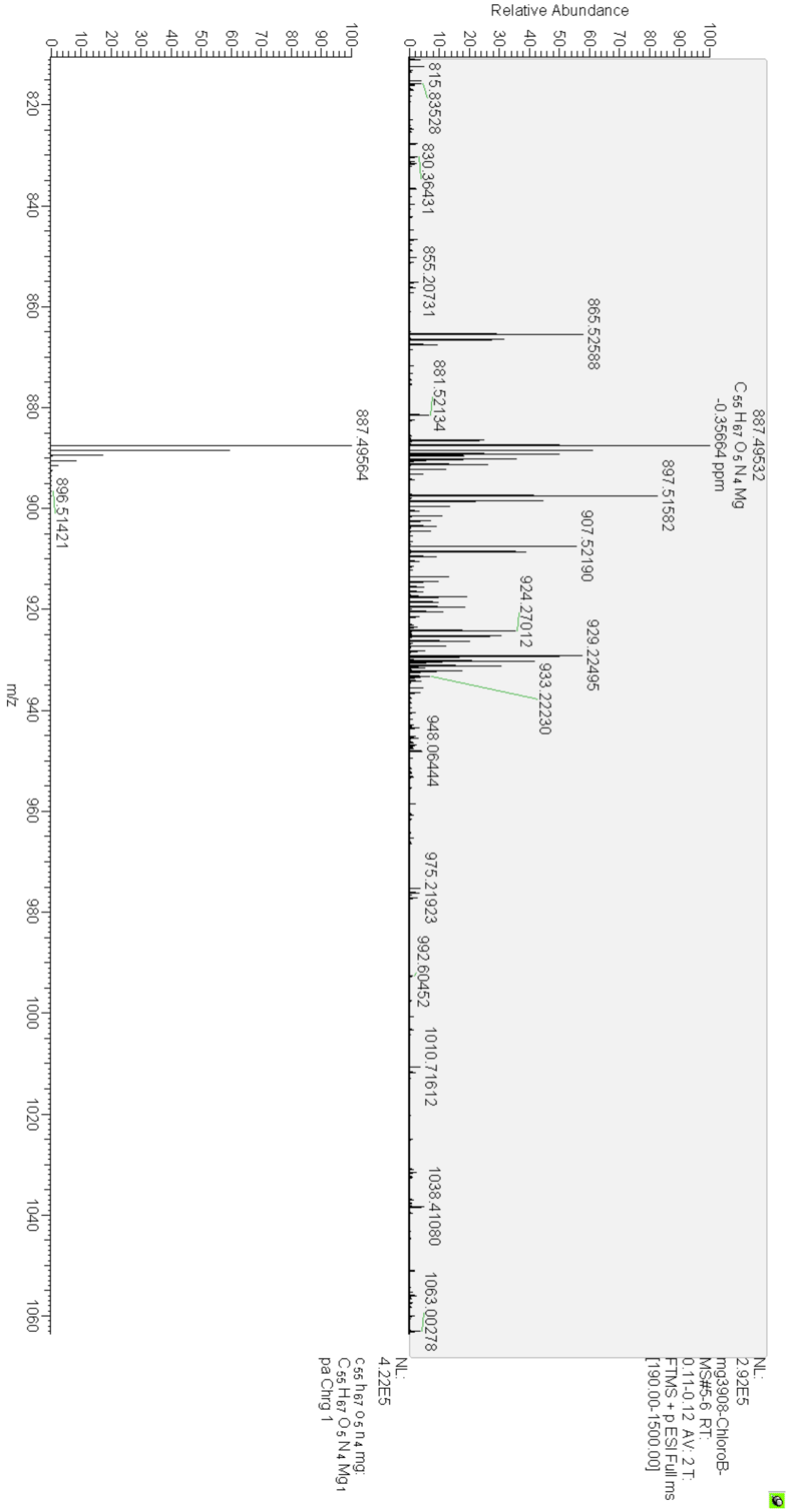

Supplementary Figure 13: High resolution MS full scan of pigment X3 in positive mode. a) MS-data relative abundance over m/z distribution. b) Simulated mass distribution of $C_{55}H_{67}O_5N_4Mg$

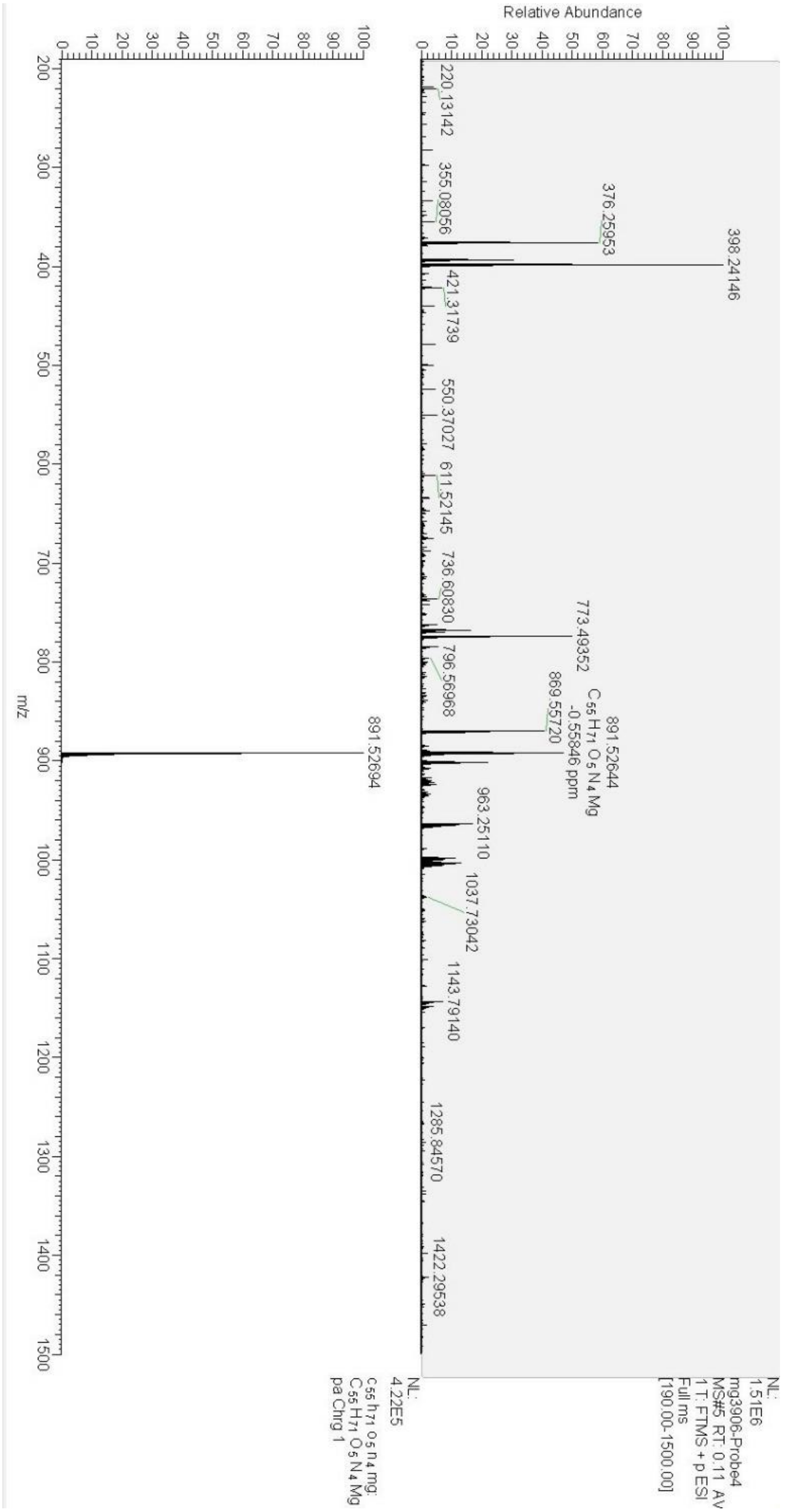

Supplementary Figure 14: High resolution MS full scan of pigment X4 in positive mode. a) MS-data relative abundance over m/z distribution. b) Simulated mass distribution of $C_{55}H_{71}O_5N_4Mg$.

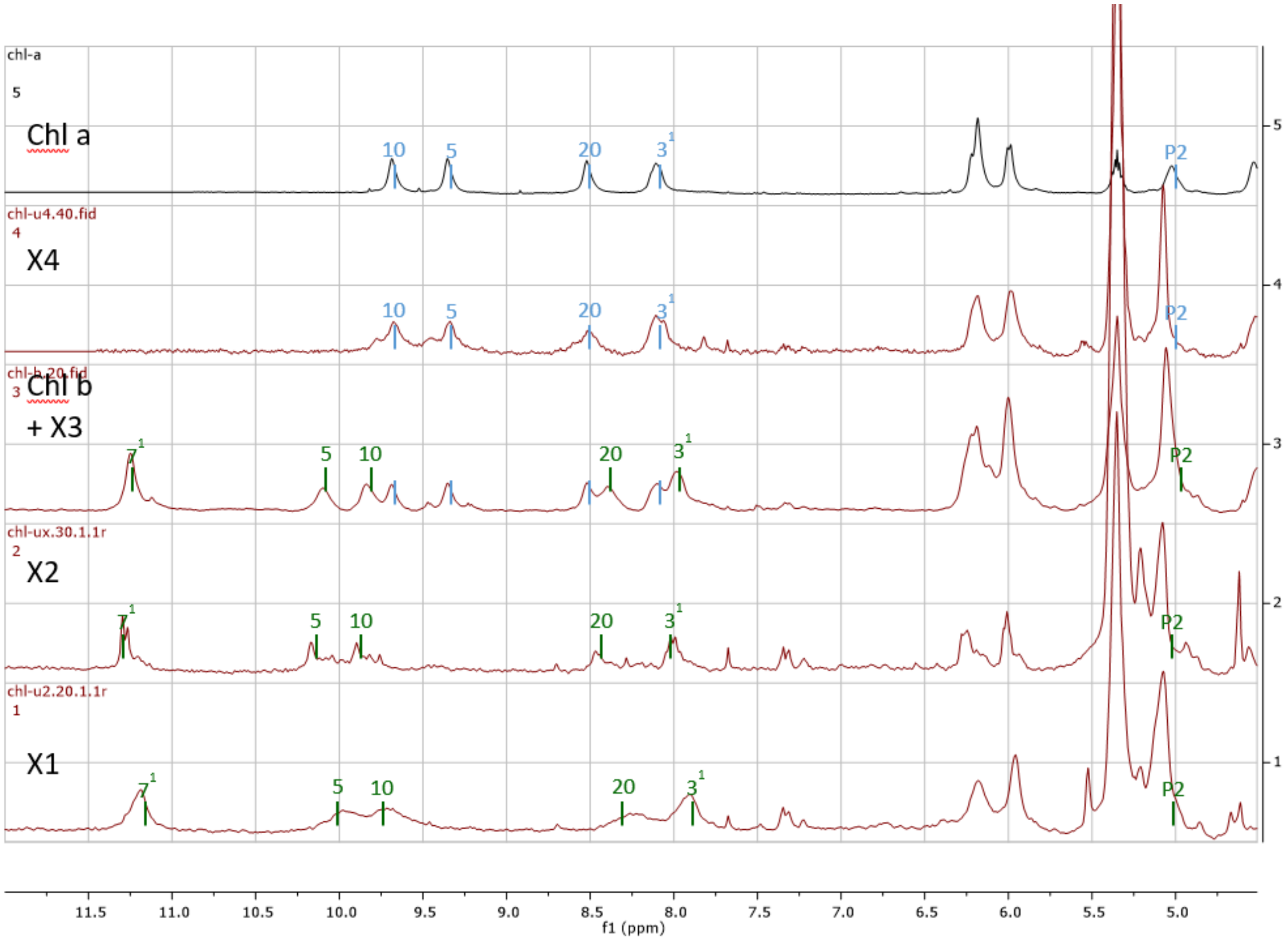

Supplementary Figure 15: Low-field ^{1}H-NMR spectra and signals of IUPAC atoms 10, 5, 20, 3^1 and P2 of Chl *a*, X4 and X3, Chl *b* with signals of IUPAC atoms 7^1, 5, 10, 20, 3^1, and P2.

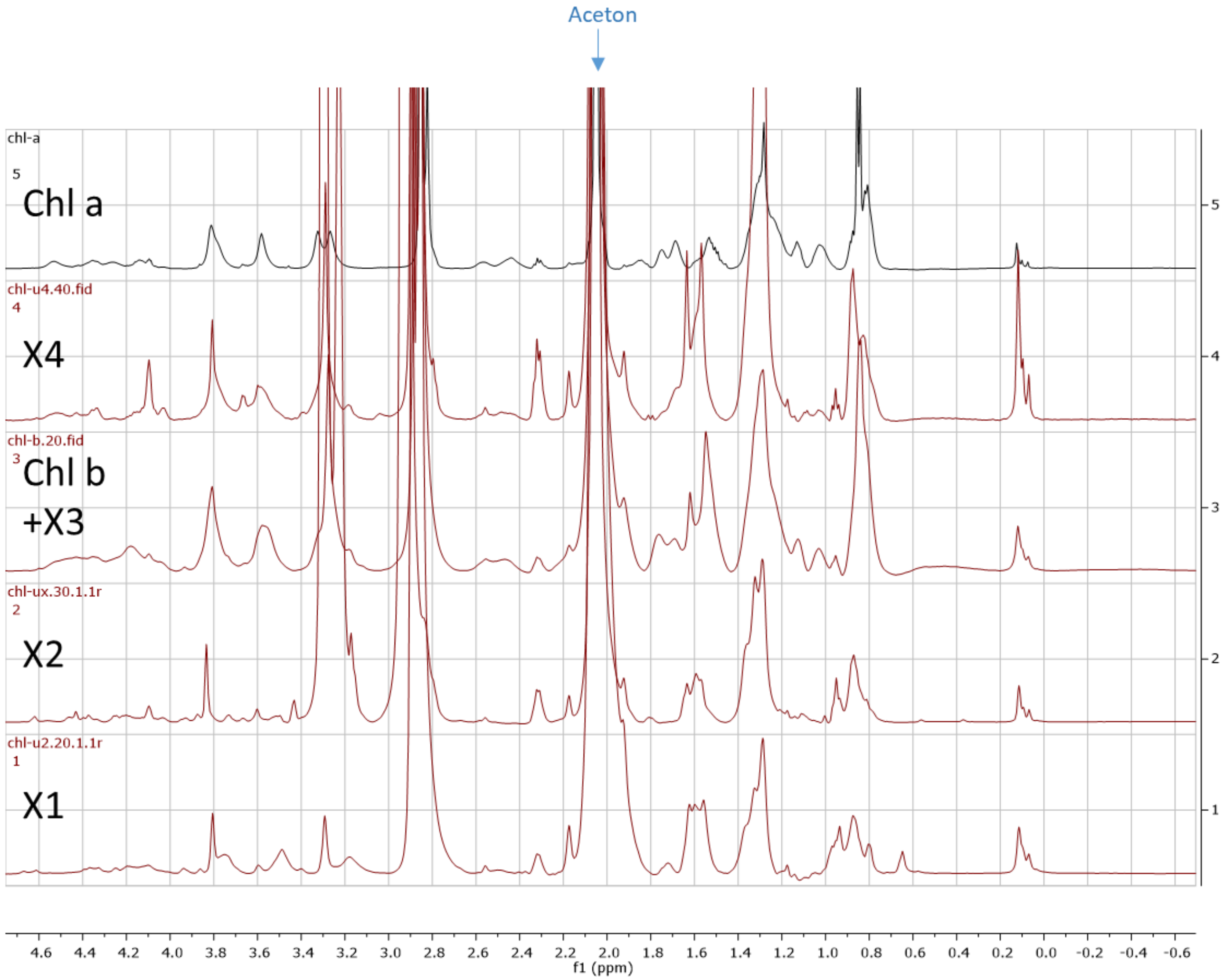

Supplementary Figure 16: High-field ^{1}H-NMR spectra of pigments Chl *a*, X4, Chl *b* and X3, X2 and X1

Supplementary Table 1: Comparison of ^{1}H- shifts of chlorophyll (Chl) *a* with X1, X2 and Chl *b* with X3, X4 in acetone-d_6 at 293 K. (Kobayashi, Akutsu et al. 2013).

IUPAC no. of carbon atom	Chl *a* #	Chl *b* #	X1	X2	Chl *a*	X3	X4	Chl *b*
2^1	3.343	3.316	3.29	3.30	3.32	3.32	3.39	3.32
3	-	-	-	-	-	-	-	-
3^1	8.162	8.043	7.91	8.02	8.11	8.10	8.11	7.98
3^2	6.242	6.302	6.18, 5.96	6.25, 6.01	6.22, 6.01	6.19	6.21, 5.98	6.22, 6.00
4	-	-	-	-	-	-	-	-
5	9.41	10.192	9.98	10.17	9.35	9.35	9.34	10.09
7^1	3.3	11.305	11.19	11.29	3.27	3.28	3.29	11.25
8	-	-	-	-	-	-	-	-
8^1	3.817	4.243	4.20	4.24	3.80		3.79	4.18
8^2	1.696	1.815		1.81	1.69	1.69	1.64	1.82
10	9.749	9.934	9.72	9.90	9.69	9.69	9.68	9.84
11	-	-	-	-	-	-	-	-
12	-	-	-	-	-	-	-	-
12^1	3.619	3.606	3.49	3.60	3.58		3.59	3.58
13	-	-	-	-	-	-	-	-
13^2	6.234	6.189		6.19	6.19		6.18	6.11
13^3	-	-	-	-	-	-	-	-
13^4	3.829	3.842	3.81	3.83	3.81		3.81	3.81
17	4.175	4.128	4.10	4.10	n/a		4.13	4.10
17^1	2.589	2.430		2.08	2.57	2.55	2.56	2.46
17^2	2.431	2.080			2.44	2.46	2.48	2.08
18	4.572	4.524	4.37	4.43	4.53		4.51	4.43
18^1	1.772	1.768	1.72		1.75			1.76
20	8.582	8.480	8.26	8.47	8.52	8.52	8.51	8.40
P1	4.342	4.364	4.33	4.37	4.34, 4.14		4.36, 4.25	4.35
P2	4.955	4.980	5.07	5.08	5.02	5.06	5.07	5.06
P3	-	-	-	-	-	-	-	-
$P3^1$	1.509	1.519	1.56		1.58	1.50	1.57	1.55
P4	1.822	1.845			1.85			
P5	1.31	1.330	1.32	1.36	1.30	1.30	1.31	1.32
P6	0.97	0.980	n/a	1.00	1.03			0.98
P7	1.31	1.330	1.32	1.36	1.31	1.30	1.33	1.32
$P7^1$	0.811	0.785	0.80	0.79	0.82, 0.81			0.78

P8	1.01	1.020			1.03			1.03
P9	1.28	1.280	1.29	1.29	1.28		1.28	1.28
P10	1.01	1.020			1.03			1.03
P11	1.31	1.320	1.32	1.32	1.31	1.30	1.33	1.32
P11[1]	0.783	0.809	0.80	0.81	0.82, 0.81			0.81
P12	1.01	1.020			1.03			1.03
P13	1.28	1.280	1.29	1.29	1.28		1.28	1.28
P14	1.12	1.120	1.18		1.13			1.13
P15	1.5	1.489			1.53			1.49
P15[1]	0.854	0.851	0.87	0.86, 0.84	0.85, 0.84			0.85, 0.84
P16	0.854	0.851	0.87	0.86, 0.84	0.85, 0.84			0.85, 0.84

Kobayashi, M., S. Akutsu, D. Fujinuma, H. Furukawa, H. Komatsu, Y. Hotota, Y. Kato, Y. Kuroiwa, T. Watanabe and M. Ohnishi-Kameyama (2013). "Physicochemical properties of chlorophylls in oxygenic photosynthesis—succession of co-factors from anoxygenic to oxygenic photosynthesis." Photosynthesis: 47-90.

Supplementary Table 2: Data used for a two-tailed t-test to determine significant differences in Chlorophyll *a* concentration for biomass under white illumination (black) and green illumination (green).

Time [d]	Concentration [mg pigment/ g_{DW}]					P-value of two-tailed t-test
	Replicate			Mean value	Standard deviation	
	1	2	3			
0.88	4.94<br>5.15	5.28<br>4.98	5.14<br>5.89	5.12<br>5.34	0.17<br>0.48	0.50
2.20	5.00<br>5.22	4.94<br>5.39	4.81<br>4.97	4.91<br>5.19	0.10<br>0.21	0.06
3.16	7.33<br>7.27	7.49<br>7.72	7.30<br>7.51	7.37<br>7.50	0.10<br>0.23	0.43
4.03	12.12<br>10.66	11.95<br>10.84	11.62<br>10.57	11.90<br>10.69	0.25<br>0.14	0.1.91E-03
5.18	16.30<br>10.25	16.26<br>10.12	16.62<br>10.02	16.39<br>10.13	0.20<br>0.11	1.11E-06
6.20	19.23<br>10.81	19.14<br>10.59	19.72<br>10.35	19.37<br>10.58	0.31<br>0.23	2.50E-06
7.01	20.96<br>11.36	21.21<br>9.85	20.81<br>10.64	20.99<br>10.62	0.20<br>0.75	2.10E-05
9.14	16.93<br>10.97	17.10<br>12.34	15.69<br>11.85	16.57<br>11.72	0.77<br>0.69	1.25E-03
10.15	13.63<br>12.23	13.36<br>12.90	13.09<br>13.17	13.36<br>12.77	0.27<br>0.49	0.14
10.89	9.64<br>11.72	9.88<br>11.85	10.21<br>11.37	9.91<br>11.65	0.28<br>0.25	1.30E-03
11.93	10.82<br>11.58	10.45<br>11.98	10.50<br>12.67	10.59<br>12.08	0.20<br>0.55	0.01
13.16	10.09<br>11.56	9.51<br>11.32	9.91<br>11.84	9.84<br>11.57	0.30<br>0.26	1.60E-03
14.08	9.01<br>10.04	9.82<br>10.67	9.31<br>10.57	9.38<br>10.43	0.41<br>0.34	0.03

Supplementary Table 3: Data used for a two-tailed t-test to determine significant differences in Chlorophyll *b* concentration for biomass under white illumination (black) and green illumination (green).

**Time [d]**	**Concentration [mg pigment/ g_{DW}]**					**P-value of two-tailed t-test**
	**Replicate**			**Mean value**	**Standard deviation**	
	**1**	**2**	**3**			
0.88	4.00<br>4.83	4.19<br>4.35	4.40<br>4.25	4.20<br>4.48	0.20<br>0.31	0.27
2.20	3.46<br>3.49	3.43<br>3.62	3.68<br>3.49	3.52<br>3.53	0.14<br>0.07	0.93
3.16	4.54<br>4.32	4.61<br>4.40	4.50<br>4.37	4.55<br>4.36	0.06<br>0.04	0.01
4.03	7.52<br>6.74	7.53<br>6.80	7.40<br>6.69	7.48<br>6.74	0.08<br>0.05	1.55E-04
5.18	11.24<br>8.04	11.21<br>7.97	11.41<br>8.03	11.29<br>8.01	0.11<br>0.04	9.85E-07
6.20	13.36<br>8.46	13.39<br>8.15	13.86<br>7.94	13.54<br>8.18	0.28<br>0.26	1.76E-05
7.01	15.46<br>8.09	15.59<br>7.35	15.35<br>7.01	15.47<br>7.76	0.12<br>0.37	4.34E-06
9.14	12.27<br>6.56	12.46<br>7.35	11.46<br>7.01	12.06<br>6.98	0.53<br>0.39	1.84E-04
10.15	9.86<br>7.64	9.65<br>8.03	9.42<br>8.21	9.64<br>7.96	0.22<br>0.29	1.33E-03
10.89	7.02<br>7.91	7.25<br>7.88	7.42<br>7.58	7.23<br>7.79	0.20<br>0.18	0.02
11.93	7.81<br>7.82	7.60<br>8.08	7.68<br>8.56	7.70<br>8.15	0.11<br>0.38	0.12
13.16	7.44<br>7.94	7.03<br>7.88	7.34<br>8.30	7.27<br>8.04	0.21<br>0.23	0.01
14.08	6.59<br>7.02	7.26<br>7.52	6.84<br>7.49	6.90<br>7.34	0.34<br>0.28	0.16

Supplementary Table 4: Data used for a two-tailed t-test to determine significant differences in concentration of pigment X1 for biomass under white illumination (black) and green illumination (green).

Time [d]	Concentration [mg pigment/ g_{DW}]					P-value of two-tailed t-test
	Replicate			Mean value	Standard deviation	
	1	2	3			
0.88	/	/	/	/	/	/
2.20	/	/	/	/	/	/
3.16	/	/	/	/	/	/
4.03	/<br>0.27	/<br>0.26	/<br>0.25	/<br>0.26	/<br>0.01	/
5.18	0.31<br>2.42	0.31<br>2.45	0.32<br>2.36	0.31<br>2.41	0.01<br>0.05	1.52E-07
6.20	0.36<br>3.15	0.33<br>2.91	0.36<br>2.85	0.35<br>2.97	0.02<br>0.16	9.36E-06
7.01	0.22<br>2.83	0.25<br>2.47	0.24<br>2.66	0.23<br>2.65	0.02<br>0.18	2.07E-05
9.14	/<br>0.70	/<br>0.79	/<br>0.76	/<br>0.75	/<br>0.05	/
10.15	/<br>0.35	/<br>0.45	/<br>0.43	/<br>0.41	/<br>0.05	/
10.89	/<br>0.26	/<br>0.27	/<br>0.26	/<br>0.26	/<br>0.01	/
11.93	/<br>0.18	/<br>0.18	/<br>0.20	/<br>0.19	/<br>0.01	/
13.16	/<br>0.13	/<br>0.13	/<br>0.13	/<br>0.13	/<br>0.00	/
14.08	/	/	/	/	/	/

Supplementary Table 5: Data used for a two-tailed t-test to determine significant differences in concentration of pigment X2 for biomass under white illumination (black) and green illumination (green).

Time [d]	Concentration [mg pigment/ g_{DW}]					P-value of two-tailed t-test
	Replicate			Mean value	Standard deviation	
	1	2	3			
0.88	/	/	/	/	/	/
2.20	/	/	/	/	/	/
3.16	/	/	/	/	/	/
4.03	/<br>0.39	/<br>0.35	/<br>0.36	/<br>0.36	/<br>0.02	/
5.18	0.78<br>1.52	0.76<br>1.56	0.82<br>1.51	0.79<br>1.53	0.03<br>0.03	5.25E-06
6.20	1.09<br>2.24	1.05<br>2.16	1.10<br>2.09	1.08<br>2.17	0.03<br>0.08	2.11E-05
7.01	1.02<br>2.56	1.01<br>2.26	0.96<br>2.46	1.00<br>2.43	0.03<br>0.16	9.86E-05
9.14	0.36<br>1.59	0.35<br>1.84	0.31<br>1.73	0.34<br>1.72	0.03<br>0.04	5.00E-05
10.15	0.19<br>1.12	0.19<br>1.20	0.21<br>1.27	0.19<br>1.19	0.01<br>0.02	1.75E-05
10.89	0.12<br>0.76	0.11<br>0.83	0.15<br>0.76	0.13<br>0.79	0.02<br>0.27	1.68E-05
11.93	0.10<br>0.52	0.10<br>0.54	0.11<br>0.56	0.11<br>0.54	0.01<br>0.02	3.31E-06
13.16	0.08<br>0.33	0.07<br>0.37	0.07<br>0.36	0.07<br>0.35	0.01<br>0.02	1.69E-05
14.08	0.08<br>0.24	0.08<br>0.25	0.06<br>0.24	0.08<br>0.24	0.01<br>0.01	3.34E-05

Supplementary Table 6: Data used for a two-tailed t-test to determine significant differences in concentration of pigment X3 for biomass under white illumination (black) and green illumination (green).

Time [d]	Concentration [mg pigment/ g_{DW}]					P-value of two-tailed t-test
	Replicate			Mean value	Standard deviation	
	1	2	3			
0.88	/	/	/	/	/	/
2.20	/	/	/	/	/	/
3.16	/	/	/	/	/	/
4.03	/<br>0.71	/<br>0.73	/<br>0.78	/<br>0.74	/<br>0.04	/
5.18	0.58<br>3.52	0.54<br>3.66	0.51<br>3.73	0.54<br>3.64	0.03<br>0.11	1.17E-06
6.20	1.67<br>3.55	1.57<br>3.37	1.71<br>3.27	1.65<br>3.40	0.07<br>0.10	3.87E-06
7.01	/<br>3.07	/<br>2.50	/<br>2.67	/<br>2.74	/<br>0.29	/
9.14	/<br>0.42	/<br>0.46	/<br>0.39	/<br>0.43	/<br>0.04	/
10.15	/<br>0.27	/<br>0.30	/<br>0.27	/<br>0.28	/<br>0.02	/
10.89	/	/	/	/	/	/
11.93	/	/	/	/	/	/
13.16	/	/	/	/	/	/
14.08	/	/	/	/	/	/

Supplementary Table 7: Data used for a two-tailed t-test to determine significant differences in concentration of pigment X4 for biomass under white illumination (black) and green illumination (green).

Time [d]	Concentration [mg pigment/ g_{DW}]					P-value of two-tailed t-test
	Replicate			Mean value	Standard deviation	
	1	2	3			
0.88	/	/	/	/	/	/
2.20	/	/	/	/	/	/
3.16	/	/	/	/	/	/
4.03	0.34<br>1.16	0.31<br>1.11	0.22<br>1.13	0.29<br>1.13	0.06<br>0.03	2.74E-05
5.18	1.57<br>2.93	1.54<br>2.97	1.61<br>2.95	1.57<br>2.95	0.03<br>0.02	3.63E-07
6.20	1.67<br>3.85	1.57<br>3.72	1.71<br>3.65	1.65<br>3.74	0.07<br>0.10	8.56E-06
7.01	1.38<br>4.13	1.38<br>3.51	1.34<br>3.85	1.37<br>3.83	0.03<br>0.31	1.67E-04
9.14	0.44<br>1.41	0.42<br>1.61	0.38<br>1.60	0.41<br>1.54	0.03<br>0.12	8.02E-05
10.15	0.21<br>0.81	0.23<br>0.84	0.22<br>0.92	0.22<br>0.86	0.01<br>0.06	4.16E-05
10.89	/<br>0.52	/<br>0.52	/<br>0.51	/<br>0.52	/<br>0.01	/
11.93	0.14<br>0.38	0.16<br>0.42	0.12<br>0.42	0.14<br>0.41	0.02<br>0.02	7.39E-05
13.16	/<br>0.27	/<br>0.30	/<br>0.30	/<br>0.29	/<br>0.02	/
14.08	/<br>0.19	/<br>0.24	/<br>0.28	/<br>0.23	/<br>0.05	/
